# NaHMDS/B(C_6_F_5_)_3_-promoted diastereoselective Friedel–Crafts alkylation of indoles/pyrroles with *N-tert*-butanesulfinylimines: towards the asymmetric synthesis of bisindole alkaloid Calcicamide B

**DOI:** 10.1039/d5ra06138e

**Published:** 2025-11-07

**Authors:** Guangshuai Zhang, Xin Chen, Yan Liu, Rui Peng, Mengwei Xu, Si Yan, Jin Xiao, Zishu Liu, Qing Min, Gang Liao, Xiaoji Wang, Shuanglin Qin

**Affiliations:** a National Engineering Research Center of Personalized Diagnostic and Therapeutic Technology, TCM Precision Medicine Research Department, FuRong Laboratory, Hunan University of Chinese Medicine Changsha 410208 P.R. China shuanglin@tju.edu.cn; b Engineering Research Center of Health Food Design & Nutrition Regulation, School of Chemical Engineering and Energy Technology, Dongguan University of Technology Dongguan 523808 P.R. China; c Xiangya School of Pharmaceutical Sciences, Furong Laboratory, Central South University Changsha 410083 Hunan China; d Hubei Engineering Research Center of Traditional Chinese Medicine of South Hubei Province, School of Pharmacy, Xianning Medical College, Hubei University of Science and Technology Xianning 437100 P.R. China

## Abstract

This study proposed an innovative and pragmatic approach to the asymmetric Friedel–Crafts reaction by employing indoles/pyrroles and chiral *N-tert*-butanesulfinylimines promoted by NaHMDS/B(C_6_F_5_)_3_. This method effectively produces enantioenriched α-(3-indolyl)glycine and α-(2-pyrrolyl)glycine derivatives, which can be readily transformed into the crucial chiral diamine skeletons. The first successful asymmetric total synthesis of marine-derived anti-tumor bisindole alkaloid Calcicamide B was achieved by employing this reaction as a crucial chiral control step.

## Abstract 2

This study proposed an innovative and pragmatic approach to the asymmetric Friedel–Crafts reaction by employing indoles/pyrroles and chiral *N-tert*-butanesulfinylimines promoted by NaHMDS/B(C_6_F_5_)_3_.

## Graphical abstract

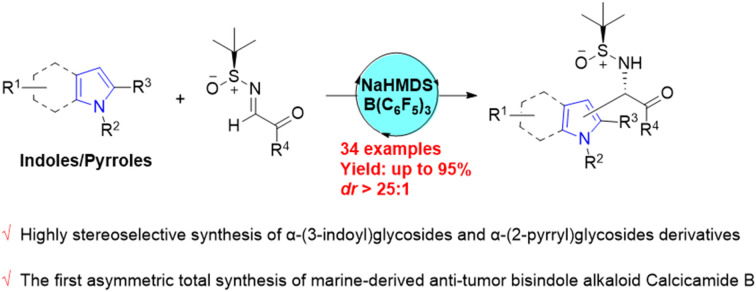

## Introduction

**Scheme 1:**
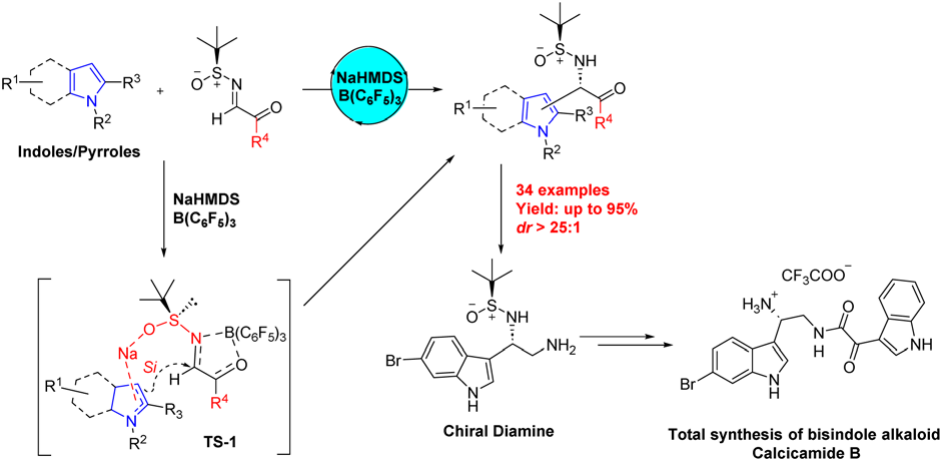
Overview.

Indoles and pyrroles, key members of the nitrogen heterocycles, have garnered considerable interest in the realms of medicinal, synthetic, and natural product chemistry due to their extensive bioactivity spectrum.^1–6^ These compounds have been integral to pharmaceutical science since its inception, especially in the realm of drug development. The current focus in this field lies on optically active non-proteinogenic amino acids, renowned for their significant biological functions and their role in organic synthesis and pioneering drug discovery. Particularly, α-(3-indolyl)glycine and α-(2-pyrrolyl)glycine, along with their variants, are prevalent in a myriad of biologically relevant natural and synthetic products, drawing substantial scientific interest.^7–9^ This has led to a focused effort towards developing stereoselective methods for their production.^10–12^ Typically, the asymmetric Friedel–Crafts interaction of indoles/pyrroles with glyoxylate imines is considered the most straightforward and accessible method, considering the ease of obtaining reactants.^13–15^ Nevertheless, there is a scarcity of techniques with wide substrate adaptability that efficiently produce these compounds in a highly enantioenriched form. The challenges in using unprotected indole substrates, controlling reaction stereoselectivity, and removing amine N-substituents are under active investigation. Boron catalysis has recently emerged as a focal point, especially since the advent of frustrated Lewis pairs (FLPs) in metal-free catalysis and the activation of small molecules.^16,17^ Among boron-based catalysts, B(C_6_F_5_)_3_ has received much attention owing to its highly electrophilic but sterically protected nature, and has the ability to reversibly bond with oxygen or nitrogen.^18–25^ In this vein, we present a novel and practical asymmetric Friedel–Crafts reaction using indoles/pyrroles and chiral *N-tert*-butanesulfinimines under NaHMDS/B(C_6_F_5_)_3_ promotion. This method efficiently yields enantioenriched α-(3-indolyl)glycines and α-(2-pyrrolyl)glycines. Utilizing this reaction as a pivotal step in chiral control, we have successfully accomplished the first asymmetric total synthesis of marine-derived anti-tumor bisindole alkaloid Calcicamide B^26,27^ (Scheme 1).

## Results and discussion

Initially, Friedel–Crafts reaction of simple indole (1a) with (*S*,*E*)-*N*-benzyl-2-((*tert*-butylsulfinyl)imino)acetamide (2a) was conducted in the presence of different Lewis acids including BF_3_·Et_2_O, Cu(OTf)_2_, AlCl_3_, and B(C_6_F_5_)_3_ at room temperature (Table 1, Entries 1–4). Despite the lack of satisfactory results, B(C_6_F_5_)_3_ served as the most effective catalyst for synthesizing product 3, yielding 79% with a diastereomeric ratio (dr) of 16 : 1 (Table 1, Entry 4). Inspired by the authors' previous research,^28,29^ bases containing alkali metal ions, such as NaHMDS, LiHMDS, KHMDS, and *t*-BuOK, were introduced into the reaction (Table 1, Entries 5–8). Fortunately, NaHMDS resulted in a higher yield (83%) and enhanced diastereoselectivity (dr > 25 : 1) (Table 1, Entry 5). However, when no catalyst is added to the reaction, the reaction cannot be carried out (Table 1, Entry 9). Various solvents were examined (Table 1, Entries 11–15), revealing that the diastereoselectivity and yields of product 3 were typically low in protic solvents (Table 1, Entries 12 and 13). Additionally, it was observed that no solvent outperformed MeCN (Table 1, Entry 8). Further testing in MeCN at different temperatures revealed that both high (Table 1, Entry 16) and low temperatures (Table 1, Entry 18) were unfavorable, with the optimal yield achieved at 0 °C (Table 1, Entry 17), maintaining high diastereoselectivity (dr > 25 : 1). Variations in the equivalents of B(C_6_F_5_)_3_ (Table 1, Entries 19–21) indicated that the most effective catalyst amount was 0.12 equivalents (Table 1, Entry 19). In summary, the conditions outlined in Entry 19 of Table 1 were optimal. Under these conditions, the reaction was completed in 3 hours, yielding 3 with an 86% yield and high diastereoselectivity (dr > 25 : 1). Other sodium salts were also used in the reaction, showing high yield and high stereoselectivity (dr > 25 : 1) (Table 1, Entries 22 and 23).

**Table 1:** Optimization of asymmetric Friedel–Crafts alkylation reaction^a^^,^^b^

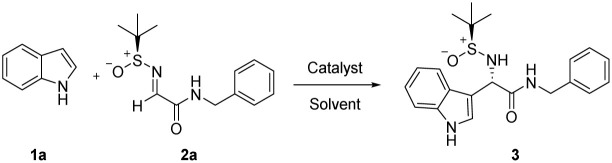						
Entry	Catalyst (mol%)	Base (mol%)	Solvent	Temp.	Yield^a^ [%]	dr^b^
1	BF_3_·Et_2_O(15)	—	MeCN	r.t.	36	5 : 1
2	Cu(OTf)_2_(15)	—	MeCN	r.t.	66	15 : 1
3	AlCl_3_(15)	—	MeCN	r.t.	0	n.d.
4	B(C_6_F_5_)_3_(15)	—	MeCN	r.t.	79	16 : 1
5	B(C_6_F_5_)_3_(15)	NaHMDS(100)	MeCN	r.t.	83	>25 : 1
6	B(C_6_F_5_)_3_(15)	LiHMDS(100)	MeCN	r.t.	80	17 : 1
7	B(C_6_F_5_)_3_(15)	KHMDS(100)	MeCN	r.t.	81	>25 : 1
8	B(C_6_F_5_)_3_(15)	*t*-BuOK(100)	MeCN	r.t.	73	>25 : 1
9	—	NaHMDS(100)	MeCN	r.t.	0	n.d.
10	B(C_6_F_5_)_3_(15)	NaHMDS(100)	DMF	r.t.	72	>25 : 1
11	B(C_6_F_5_)_3_(15)	NaHMDS(100)	THF	r.t.	65	>25 : 1
12	B(C_6_F_5_)_3_(15)	NaHMDS(100)	MeOH	r.t.	66	10 : 1
13	B(C_6_F_5_)_3_(15)	NaHMDS(100)	EtOH	r.t.	63	12 : 1
14	B(C_6_F_5_)_3_(15)	NaHMDS(100)	DMSO	r.t.	82	7 : 1
15	B(C_6_F_5_)_3_(15)	NaHMDS(100)	Acetone	r.t.	70	15 : 1
16	B(C_6_F_5_)_3_(15)	NaHMDS(100)	MeCN	50 °C	79	>25 : 1
17	B(C_6_F_5_)_3_(15)	NaHMDS(100)	MeCN	0 °C	85	>25 : 1
18	B(C_6_F_5_)_3_(15)	NaHMDS(100)	MeCN	−20 °C	80	>25 : 1
19	B(C_6_F_5_)_3_(12)	NaHMDS(100)	MeCN	0 °C	86	>25 : 1
20	B(C_6_F_5_)_3_(10)	NaHMDS(100)	MeCN	0 °C	81	>25 : 1
21	B(C_6_F_5_)_3_(5)	NaHMDS(100)	MeCN	0 °C	79	>25 : 1
22	B(C_6_F_5_)_3_(12)	NaBF_4_(100)	MeCN	0 °C	82	>25 : 1
23	B(C_6_F_5_)_3_(12)	*t*-BuONa(100)	MeCN	0 °C	80	>25 : 1

a Isolated yields.

b The values of dr were determined using ^1^H NMR. n.d. = not determined.

The studies of Friedel–Crafts reaction conditions indicated that the reaction could be further investigated in terms of substrate scope, covering various indoles/pyrroles with different *N-tert*-butanesulfinylimines.

As depicted in Table 2, we first screened the different indole substrates. We found that electron-donating group substitution on indole promoted the reaction (4–6), while electron-withdrawing groups on indole reduced the reaction yield (7, 8). Using indoles with halogens as substituents on the benzenic ring, the reaction could proceed well (9–13, yield 79–88%, dr > 25 : 1). Methyl substitution at position 2 of indole did not affect yield and stereoselectivity (14). Indeed, an electron-donating group (*e.g.* methyl, phenyl) on the nitrogen atom of indole resulted in a good reaction results (15–16, yield 82–93%, dr > 25 : 1), but when there were electron-withdrawing substitutions (*e.g.* TS, Boc) on the nitrogen atom of indole, the reaction failed to proceed (17, 18).

**Table 2:** Diastereoselective Friedel–Crafts reaction of indoles with (*S*)-*N-tert*-butanesulfinylimine 2a^a^^,^^b^^,^^c^

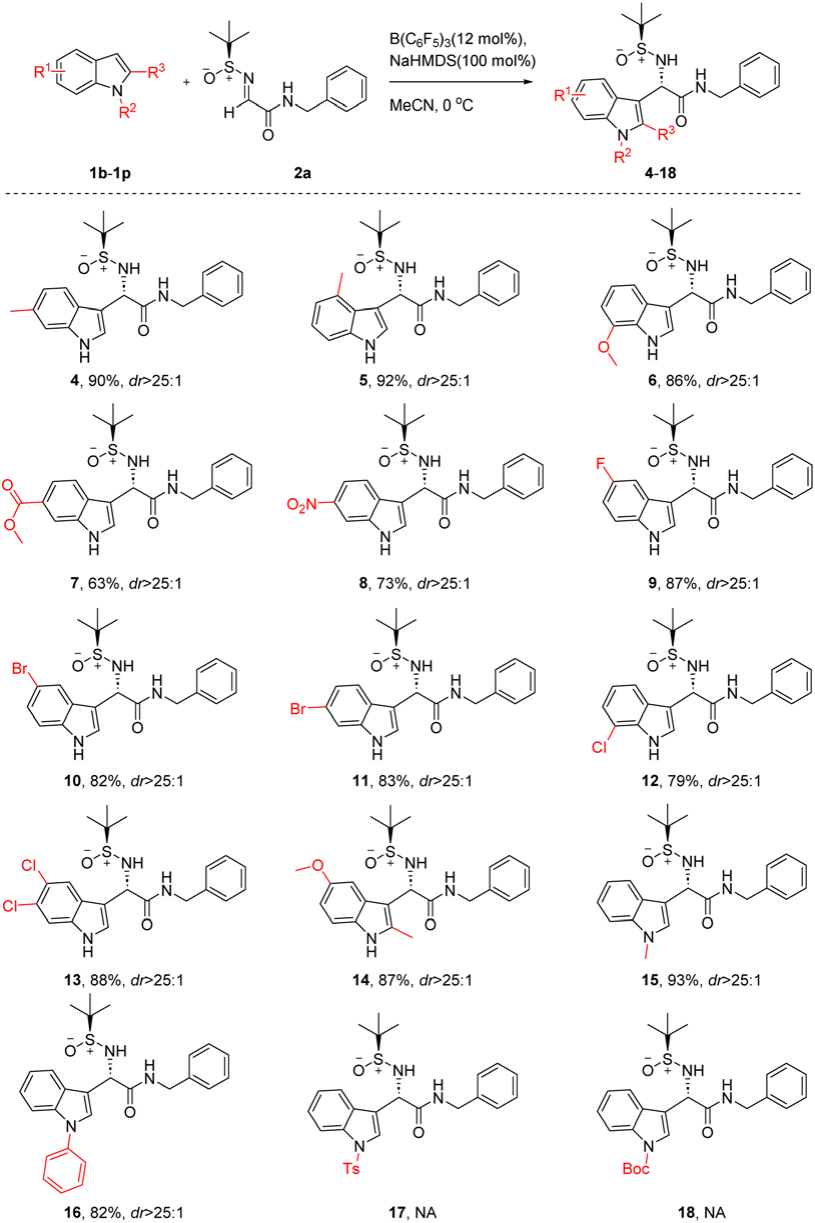

a Isolated yields.

b The dr values were determined using ^1^H NMR. NA = not available.

c When NaHMDS was not added to the reaction, the dr of compound 15 is 15 : 1, and the dr of compound 16 is 13 : 1.

Subsequently, this study focused on investigating the applicability of the novel Friedel–Crafts reaction with various imines, as detailed in Table 3. To facilitate comparative analysis, all reactions were performed under standardized conditions. The outcomes demonstrated that when the R groups of the substrates were aliphatic amines (19–24) and aromatic amines (25, 26), the reactions consistently yielded high efficiency (79–91%) and pronounced stereoselectivity (dr > 25 : 1).

**Table 3:** Diastereoselective Friedel–Crafts reaction of indole with various *N-tert*-butanesulfinylimines^a^^,^^b^

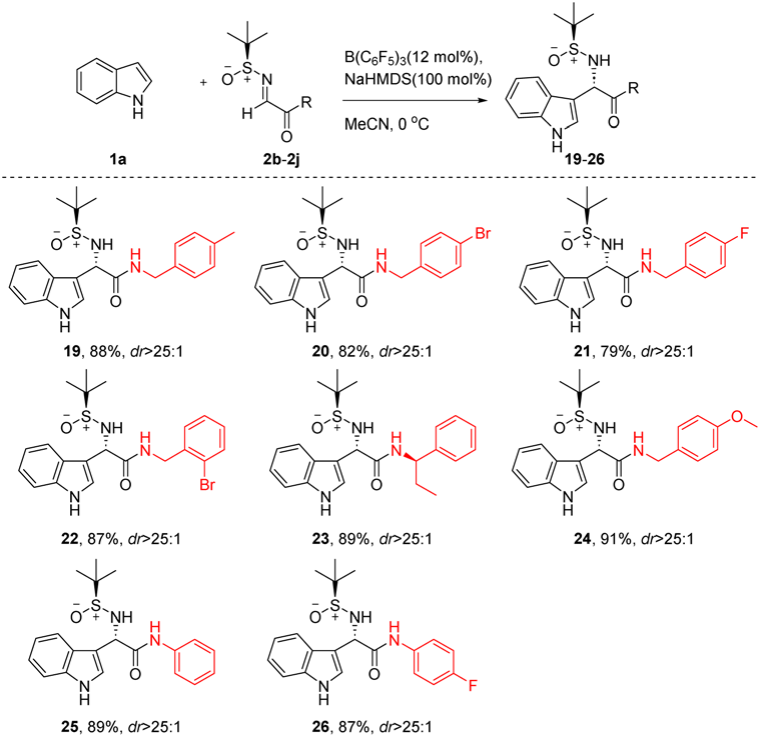

a Isolated yields.

b The values of dr were determined using ^1^H NMR.

Encouraged by above success, we extended our approach to include pyrroles as substrates (Table 4). The reactions using pyrroles as substrates demonstrated a similar trend to that observed with indole substrates (27–30). Reactions proceeded smoothly without substitution or with electron-donating groups on the nitrogen atom of pyrrole, yielding high stereoselectivity and high yield (27–29). Conversely, the presence of an electron-withdrawing group on the nitrogen atom of pyrrole hindered the reaction (30).

**Table 4:** Diastereoselective Friedel–Crafts reaction of pyrrole with various *N-tert*-butanesulfinylimines^a^^,^^b^

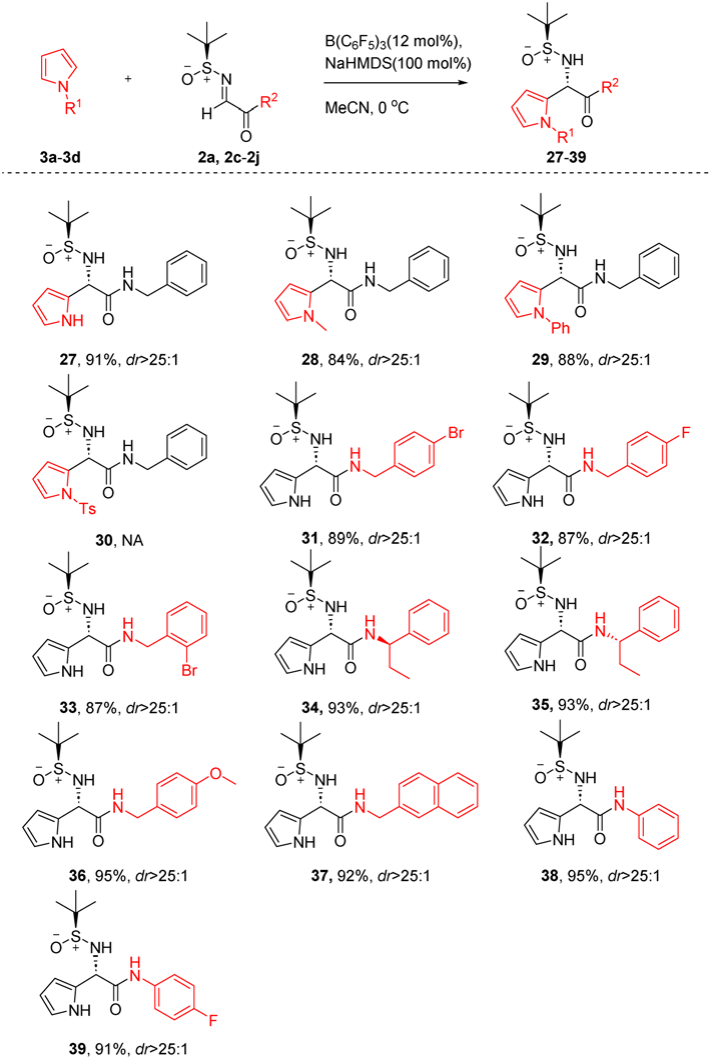

a Isolated yields.

b The values of dr were determined using ^1^H NMR. NA = not available.

Fortunately, under standard conditions, we utilized pyrrole as a substrate and reacted it with a range of chiral imines (Table 4), R groups including aliphatic amines (31–37) and aromatic amines (38–39) to synthesize a series of α-(2-pyrrolyl)glycine derivatives, achieving high yields and pronounced stereoselectivity. These outcomes demonstrate the versatility and general applicability of the reaction conditions.

**Scheme 2:**
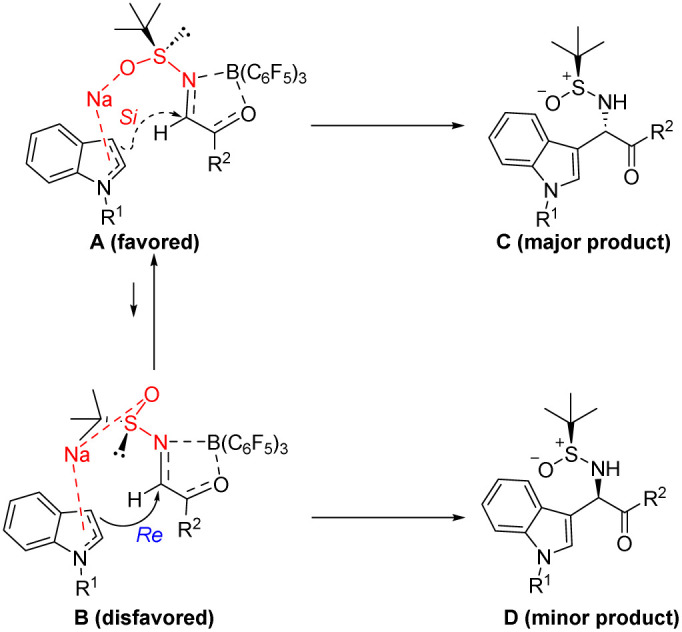
Possible mechanism.

Based on prior studies and literature,^28–31^Scheme 2 elucidates the proposed mechanism. Here, B(C_6_F_5_)_3_ coordinates with the imine nitrogen and carbonyl oxygen. This configuration promotes the transition state A, with the *N*-sulfinyl group adopting a synperiplanar (s-*cis*) conformation.^32–36^ The Na^+^ coordinate with the oxygen of the *N*-sulfinyl group, while the indole coordinate with Na^+^ by p-π activation of the delocalized π bond, thereby enhancing the reaction's stereoselectivity.^37–40^ Specifically, with (*S*)-*N-tert*-butylsulfinyl substrates, indole attacks from the less hindered *Si*-side of the C=N bond, avoiding steric hindrance with the bulky *tert*-butyl group, resulting in the (*S*)-product C formation (Scheme 2).

**Scheme 3:**
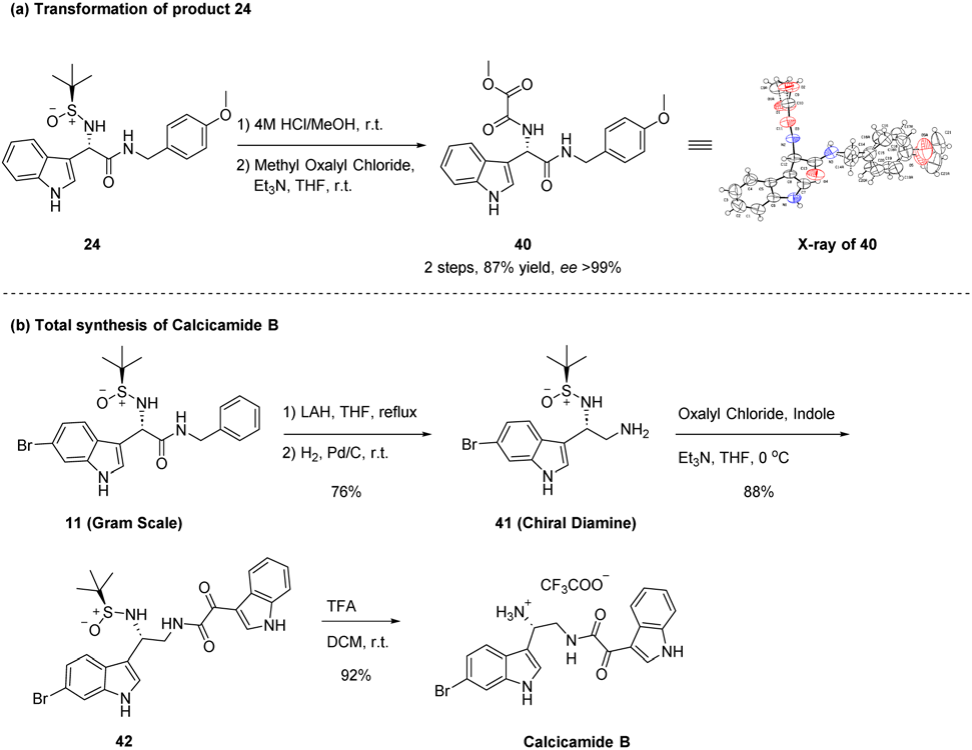
(a) Preparation of compound 40 from product 24. The ee value of 40 were determined by chiral HPLC. (b) Total synthesis of the bisindole alkaloid Calcicamide B.

All of the above products from the Friedel–Crafts reaction, as described in Tables 2–4, were not crystalline. Therefore, we derivatized these products to yield compounds that could readily form single crystals. As illustrated in Scheme 3a, compound 24 underwent removal of the *tert*-butylsulfinyl group from its chiral nitrogen atom under acidic conditions, followed by a reaction with oxalyl methyl chloride in an alkaline environment to synthesize compound 40 (ee > 99%). Fortunately, we successfully obtained the single crystal structure of compound 40 (CCDC 2322830), confirming the stereoselectivity of this Friedel–Crafts reaction (Scheme 3a).

It is noteworthy that α-(3-indolyl)glycines and α-(2-pyrrolyl)glycines synthesized by this asymmetric Friedel–Crafts reaction can be easily converted into chiral diamines, which are important intermediates in the synthesis of natural products and pharmaceuticals. To delve deeper into the feasibility of this Friedel–Crafts reaction, this study employed it as a crucial step in the chiral induction for the total synthesis of marine-derived anti-tumor bisindole alkaloid Calcicamide B. As illustrated in Scheme 3b, compound 11, which was successfully synthesized on a gram scale (Table 2), was reduced using LiAlH_4_, then benzyl group was removed to form chiral diamine 41. Indole upon reaction with oxalyl chloride and subsequent condensation with intermediate 41 could yield precursor 42, and subsequently, TFA-catalyzed hydrolysis was performed to remove the *tert*-butylsulfinyl group, resulting in anti-tumor bisindole alkaloid Calcicamide B (Scheme 3b).

## Conclusions

In summary, we have developed a highly diastereoselective Friedel–Crafts alkylation for both protected and unprotected indoles/pyrroles using *N-tert*-butanesulfinylimines, directly yielding various enantioenriched α-(3-indolyl)glycine and α-(2-pyrrolyl)glycine derivatives. This reaction proceeds easily in the presence of NaHMDS/B(C_6_F_5_)_3_, achieving high yields and exhibiting excellent functional group tolerance. This method holds great potential for future applications of optically active α-(3-indolyl)glycine and α-(2-pyrrolyl)glycine derivatives in medicinal chemistry and organic synthesis. Furthermore, the crucial chiral diamine skeleton can be obtained through a straightforward conversion of the product derived from the reaction. Utilizing this reaction as a key chiral control step, we accomplished the first asymmetric total synthesis of marine-derived anti-tumor bisindole alkaloid Calcicamide B.

## Author contributions

All authors have read and agreed to the published version of the manuscript. Conceptualization, G. Z. and X. C.; methodology, G. Z., X. C. and Y. L.; validation, R. P., M. X., S. Y., J. X., Z. L. and Q. M.; writing—original draft preparation, G. Z., X. C. and Y. L.; writing—review and editing, G. Z., G. L., X. W. and S. Q.; supervision, G. L., X. W. and S. Q.; funding acquisition, G. L., X. W. and S. Q.

## Conflicts of interest

There are no conflicts to declare.

## Supplementary Material

RA-015-D5RA06138E-s001

RA-015-D5RA06138E-s002

## Data Availability

CCDC 2322830 (40) contains the supplementary crystallographic data for this paper.^41^ The data supporting this article have been included as part of the supplementary information (SI). Supplementary information: experimental procedures and spectroscopic data. See DOI: https://doi.org/10.1039/d5ra06138e.
