# Supplementary material for: NaHMDS/B(C6F5)3-promoted diastereoselective Friedel–Crafts alkylation of indoles/pyrroles with N-tert-butanesulfinylimines: towards the asymmetric synthesis of bisindole alkaloid Calcicamide B
Source: RSC Adv. 2025 Nov 7;15(51):43421–5. doi: 10.1039/d5ra06138e (PMC12593423; doi:10.1039/d5ra06138e)

Supplementary Information (SI) for RSC Advances.
This journal is © The Royal Society of Chemistry 2025

NaHMDS/B(C_6F_5)$_3$-Promoted Diastereoselective Friedel-Crafts Alkylation of Indoles/Pyrroles with *N-tert*-butanesulfinylimines: Towards the Asymmetric Synthesis of Bisindole Alkaloid Calcicamide B

Guangshuai Zhang,[ad†] Xin Chen,[bd†] Yan Liu,[cd†] Rui Peng,[d] Mengwei Xu,[d] Si Yan,[d] Jin Xiao,[a] Zishu Liu,[a] Qing Min,[d] Gang Liao,*[c] Xiaoji Wang,*[b] and Shuanglin Qin*[ad]

[a.] *National Engineering Research Center of Personalized Diagnostic and Therapeutic Technology, TCM Precision Medicine Research Department, FuRong Laboratory, Hunan University of Chinese Medicine, Changsha 410208, P.R. China, E-mail: shuanglin@tju.edu.cn*

[b.] *Engineering Research Center of Health Food Design & Nutrition Regulation, School of Chemical Engineering and Energy Technology, Dongguan University of Technology, Dongguan 523808, P.R. China*

[c.] *Xiangya School of Pharmaceutical Sciences, Furong Laboratory, Central South University, Changsha 410083, Hunan, China*

[d.] *Hubei Engineering Research Center of Traditional Chinese Medicine of South Hubei Province, School of Pharmacy, Xianning Medical College, Hubei University of Science and Technology, Xianning 437100, P.R. China*

Contents

1. General information S1
2. Preparation of *N-tert*-butanesulfinimines **2a-2j** S2
3. NaHMDS/B(C_6F_5)$_3$-promoted diastereoselective Friedel-Crafts alkylation of indoles/pyrroles with *N-tert*-butanesulfinimines S5
4. The synthesis of derivative **40**, determination of *ee* value by HPLC, and single crystal data S20
5. Total synthesis of the bisindole alkaloid Calcicamide B S23
6. NMR spectra of compounds S25

1. General information

All reactions were carried out under an argon atmosphere with dry, freshly distilled solvents under anhydrous conditions, unless otherwise noted. Yields refer to chromatographically and spectroscopically (^{1}H NMR) homogeneous materials, unless otherwise stated. The used solvents were purified and dried according to common procedures. Other chemicals and solvents were commercially available. High-resolution mass spectra (HRMS) were obtained with a FTICR-MS (Ion spec 7.0T) spectrometer. ^{1}H NMR spectra were obtained by using a Bruker AV 400 or AV 600. Chemical shifts are reported in parts per million (ppm) relative to either a tetramethylsilane internal standard or solvent signals. Data are reported as follows: chemical shift, multiplicity (s = singlet, d = doublet, t = triplet, q = quartet, br = broad, m = multiplet), coupling constants and integration. ^{13}C

NMR spectra were recorded using a Bruker AV 400 spectrometer (100 MHz) using $CDCl_3$ as the solvent. Chemical shifts (δ) are reported in parts per million measured relative to the solvent peak. Melting points (m.p.) were obtained on a Mel-Temp capillary melting point apparatus. Enantiomeric excess(*ee*) was determined using Essentia LC-16. IR spectra were recorded with a Bio-Rad FTS 6000 Fourier infrared spectrometer.

2. Preparation of *N*-*tert*-butanesulfinimines 2a-2j

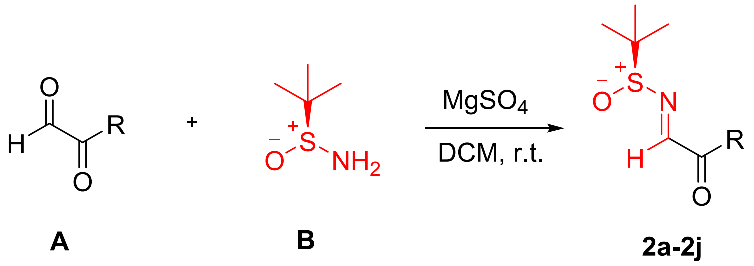

Representative Procedure:

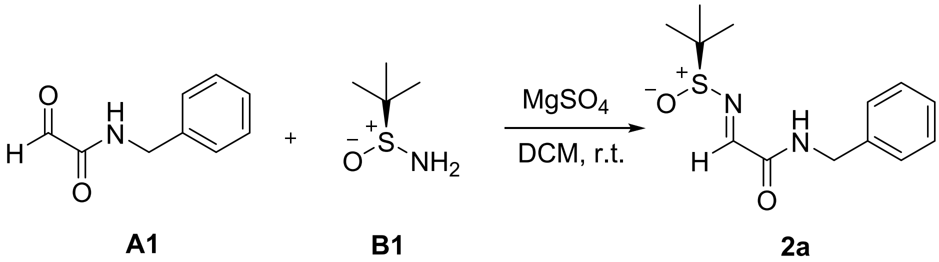

In a round bottom flask, aldehyde (**A1**) was dissolved in DCM, then *S*-*tert*-butylsulfinamide (**B1**) and anhydrous magnesium sulfate were added successively. Stirring at room temperature under the protection of argon gas, and monitor the reaction using thin layer chromatography (TLC). After the reaction was completed, filter out anhydrous magnesium sulfate with diatomaceous earth. Add $NaHCO_3$ aqueous solution to the filtrate, extract 3 times with DCM, collect the organic layer, dry with anhydrous sodium sulfate, concentrate, and purify by column chromatography to obtain a clean product **2a**.

(*S,E*)-*N*-benzyl-2-((*tert*-butylsulfinyl)imino)acetamide **2a** as a pale yellow oil: $[\alpha]_D^{20}$= + 193.28 (c 0.1, MeOH). ^{1}H NMR (400 MHz, $CDCl_3$) δ 8.00 (s, 1H), 7.39 – 7.35 (m, 1H), 7.35 (d, *J* = 0.8 Hz, 1H), 7.33 – 7.30 (m, 2H), 7.30 – 7.29 (m, 1H), 7.10 (s, 1H), 4.64 (dd, *J* = 14.9, 6.4 Hz, 1H), 4.51 (dd, *J* = 14.9, 5.7 Hz, 1H), 1.24 (s, 9H). ^{13}C NMR (100 MHz, $CDCl_3$) δ 160.7, 158.6, 137.3, 128.8, 127.7, 58.5, 43.4, 22.6. HRMS (ESI) calculated for $C_{13}H_{19}N_2O_2S^+[M+H]^+$: 267.1162, found: 267.1164.

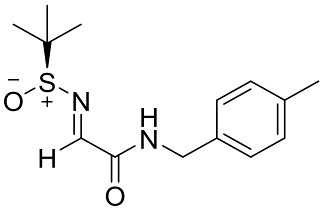

compound **2b**

(*S,E*)-2-((*tert*-butylsulfinyl)imino)-*N*-(4-methylbenzyl)acetamide **2b** as a pale yellow oil: $[\alpha]_D^{20}$=+ 173.55 (c 0.1, MeOH). ^{1}H NMR (400 MHz, $CDCl_3$) δ 7.98 (s, 1H), 7.17 (q, *J* = 8.2 Hz, 5H), 4.57 (dd, *J* = 14.8, 6.3 Hz, 1H), 4.46 (dd, *J* = 14.6, 5.9 Hz, 1H), 2.34 (s, 3H), 1.23 (s, 9H). ^{13}C NMR (100 MHz, $CDCl_3$) δ 188.3, 160.6, 158.7, 137.6, 134.2, 129.5, 127.8, 58.6, 43.3, 22.7, 21.1. HRMS (ESI) calculated for $C_{14}H_{21}N_2O_2S^+[M+H]^+$: 281.1318, found: 281.1319.

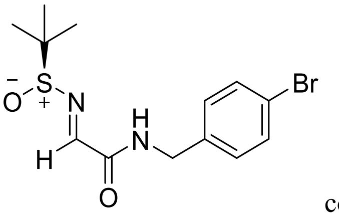

compound **2c**

(*S,E*)-*N*-(4-bromobenzyl)-2-((*tert*-butylsulfinyl)imino)acetamide **2c** as a pale yellow oil: $[\alpha]_D^{20}$=+ 159.88 (c 0.1, MeOH). ^{1}H NMR (400 MHz, $CDCl_3$) δ 7.98 (s, 1H), 7.46 (d, *J* = 8.4 Hz, 2H), 7.18 (d, *J* = 8.2 Hz, 2H), 4.56 (dd, *J* = 15.1, 6.5 Hz, 1H), 4.45 (dd, *J* = 15.1, 5.9 Hz, 1H), 1.23 (s, 9H). ^{13}C NMR (100 MHz, $CDCl_3$) δ 160.7, 158.5, 136.4, 131.9, 129.5, 121.7, 58.6, 42.8, 22.7. HRMS (ESI) calculated for $C_{13}H_{18}BrN_2O_2S^+[M+H]^+$: 345.0627, found: 345.0629.

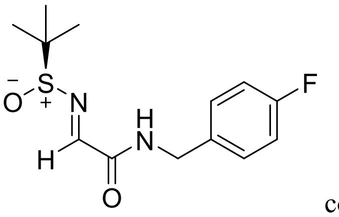

compound **2d**

(*S,E*)-2-((*tert*-butylsulfinyl)imino)-*N*-(4-fluorobenzyl)acetamide **2d** as a pale yellow oil: $[\alpha]_D^{20}$= + 199.72 (c 0.1, MeOH). ^{1}H NMR (400 MHz, $CDCl_3$) δ 7.96 (s, 1H), 7.60 (t, *J* = 6.2 Hz, 1H), 7.27 (dd, *J* = 8.5, 5.4 Hz, 2H), 7.00 (t, *J* = 8.7 Hz, 2H), 4.57 (dd, *J* = 14.9, 6.4 Hz, 1H), 4.46 (dd, *J* = 14.9, 5.9 Hz, 1H), 1.22 (s, 9H). ^{13}C NMR (100 MHz, $CDCl_3$) δ 163.4, 161.0, 160.7, 158.5, 133.3, 133.3, 129.5, 129.4, 115.7, 115.4, 58.5, 42.7, 22.6. ^{19}F NMR (376 MHz, $CDCl_3$) δ -117.34. HRMS (ESI) calculated for $C_{13}H_{18}FN_2O_2S^+[M+H]^+$: 285.1068, found: 285.1068.

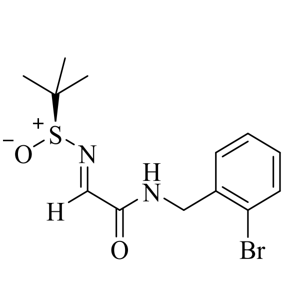

compound **2e**

(*S,E*)-*N*-(2-bromobenzyl)-2-((*tert*-butylsulfinyl)imino)acetamide **2e** as a pale yellow oil: $[\alpha]_D^{20}$= + 163.77 (c 0.1, MeOH). ^{1}H NMR (400 MHz, $CDCl_3$) δ 7.97 (s, 1H), 7.58 (dd, *J* = 8.0, 1.3 Hz, 1H), 7.40 (dd, *J* = 7.6, 1.7 Hz, 1H), 7.31 (td, *J* = 7.5, 1.3 Hz, 2H), 7.18 (td, *J* = 7.7, 1.8 Hz, 1H), 4.71 – 4.55 (m, 2H), 1.25 (s, 9H). ^{13}C NMR (100 MHz, $CDCl_3$) δ 160.6, 158.5, 136.4, 133.0, 130.5, 129.6, 127.9, 123.8, 58.6, 43.8, 22.7. HRMS (ESI) calculated for $C_{13}H_{18}BrN_2O_2S^+[M+H]^+$: 345.0267,

found: 345.0269.

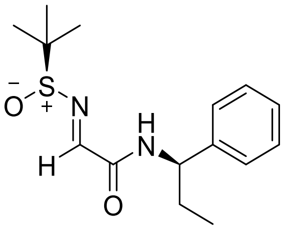

compound **2f**

(*E*)-2-(((*S*)-*tert*-butylsulfinyl)imino)-*N*-((*R*)-1-phenylpropyl)acetamide **2f** as a pale yellow oil: $[\alpha]_D^{20}$= + 99.34 (c 0.1, MeOH). ^{1}H NMR (400 MHz, $CDCl_3$) δ 7.95 (s, 1H), 7.33 (dtd, *J* = 16.5, 7.7, 6.3 Hz, 2H), 7.17 (d, *J* = 8.6 Hz, 1H), 4.95 (q, *J* = 7.7 Hz, 1H), 1.92 (p, *J* = 7.4 Hz, 2H), 1.26 (s, 9H), 0.92 (t, *J* = 7.4 Hz, 3H). ^{13}C NMR (100 MHz, $CDCl_3$) δ 160.0, 158.9, 141.2, 128.8, 127.7, 126.6, 58.6, 55.1, 29.0, 22.7, 10.7. HRMS (ESI) calculated for $C_{15}H_{23}N_2O_2S^+[M+H]^+$: 295.1475, found: 295.1476.

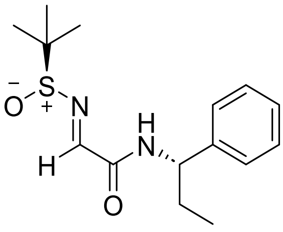

compound **2g**

(*E*)-2-(((*S*)-*tert*-butylsulfinyl)imino)-*N*-((*S*)-1-phenylpropyl)acetamide **2g** as a pale yellow oil: $[\alpha]_D^{20}$= + 93.47 (c 0.1, MeOH). ^{1}H NMR (400 MHz, $CDCl_3$) δ 7.95 (s, 1H), 7.34 (d, *J* = 7.2 Hz, 2H), 7.31 – 7.25 (m, 3H), 7.01 (s, 1H), 4.95 (q, *J* = 7.7 Hz, 1H), 1.91 (dq, *J* = 14.2, 7.0 Hz, 2H), 1.23 (s, 9H), 0.92 (t, *J* = 7.3 Hz, 3H). ^{13}C NMR (100 MHz, $CDCl_3$) δ 160.0, 158.8, 141.2, 128.7, 127.6, 126.3, 58.5, 55.0, 29.2, 22.6, 10.6. HRMS (ESI) calculated for $C_{15}H_{23}N_2O_2S^+[M+H]^+$: 295.1475, found: 295.1477.

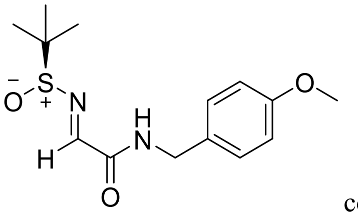

compound **2h**

(*S,E*)-2-((*tert*-butylsulfinyl)imino)-*N*-(4-methoxybenzyl)acetamide **2h** as a pale yellow oil: $[\alpha]_D^{20}$= + 138.82 (c 0.1, MeOH). ^{1}H NMR (400 MHz, $CDCl_3$) δ 7.98 (s, 1H), 7.28 – 7.21 (m, 2H), 7.10 (d, *J* = 6.2 Hz, 1H), 6.91 – 6.83 (m, 2H), 4.56 (dd, *J* = 14.7, 6.3 Hz, 1H), 4.48 – 4.37 (m, 1H), 3.80 (s, 3H), 1.23 (s, 9H). ^{13}C NMR (100 MHz, $CDCl_3$) δ 188.3, 160.6, 159.3, 158.7, 129.3, 129.2, 114.2, 58.6, 55.3, 43.0, 22.7. HRMS (ESI) calculated for $C_{14}H_{21}N_2O_3S^+[M+H]^+$: 297.1267, found: 297.1269.

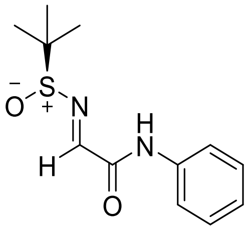

compound **2i**

(*S,E*)-2-((*tert*-butylsulfinyl)imino)-*N*-phenylacetamide **2i** as a pale yellow oil: $[\alpha]_D^{20}$= + 87.06 (c 0.2, MeOH). ^{1}H NMR (400 MHz, $CDCl_3$) δ 8.60 (s, 1H), 8.07 (s, 1H), 7.74 – 7.62 (m, 2H), 7.40 (t, J = 7.9 Hz, 2H), 7.20 (t, J = 7.4 Hz, 1H), 1.32 (s, 9H). ^{13}C NMR (100 MHz, $CDCl_3$) δ 159.2, 158.2, 136.6, 129.2, 125.3, 119.8, 58.9, 22.8. HRMS (ESI) calculated for $C_{12}H_{17}N_2O_2S^+[M+H]^+$: 253.1005, found: 253.1008.

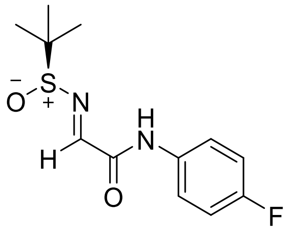

compound **2j**

(*S,E*)-2-((*tert*-butylsulfinyl)imino)-*N*-(4-fluorophenyl)acetamide **2j** as a pale yellow oil: $[\alpha]_D^{20}$= + 173.68 (c 0.2, MeOH). ^{1}H NMR (400 MHz, $CDCl_3$) δ 8.46 (s, 1H), 7.97 (s, 1H), 7.60 – 7.49 (m, 2H), 7.00 (t, *J* = 8.6 Hz, 2H), 1.23 (s, 9H). ^{13}C NMR (100 MHz, $CDCl_3$) δ 161.1, 159.0, 158.6, 158.2, 132.6, 121.6, 121.5, 116.1, 115.9, 58.9, 22.8. ^{19}F NMR (376 MHz, $CDCl_3$) δ -116.37. HRMS (ESI) calculated for $C_{12}H_{16}FN_2O_2S^+[M+H]^+$: 271.0911, found: 271.0914.

3. NaHMDS/B(C_6F_5)$_3$-promoted diastereoselective Friedel-Crafts alkylation of indoles/pyrroles with *N-tert*-butanesulfinimines

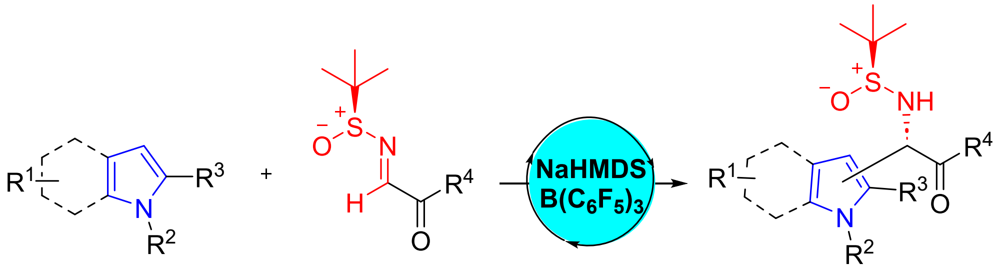

Representative Procedure:

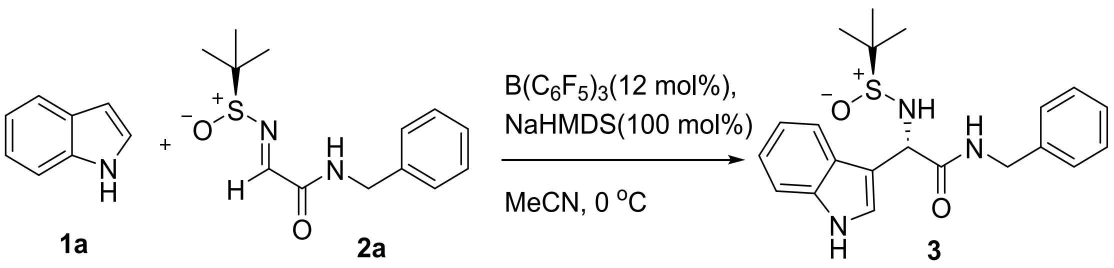

In a round bottom flask, to a mixture of compound **1a** (0.14g, 1.20 mmol), B(C_6F_5)$_3$ (0.06g, 0.12

mmol) and NaHMDS (1.2 mL, 1 mol/L, 1.20 mmol) in MeCN (5 mL) being coolen to 0 °C was added the **2a** (0.27g, 1.00 mmol). After the substrate was completely consumed (monitored by TLC analysis), washed the reaction mixture with water (20 mL), The mixture was extracted by ethyl acetate (3 × 20 mL). The combined organic phase was dried over Na_2SO_4, and concentrated in *vacuo*. The residue was purified by flash column chromatography on silica gel (petroleum ether:ethyl acetate = 1:3) to furnish the desired compound **3**.

(S)-N-benzyl-2-(((*S*)-*tert*-butylsulfinyl)amino)-2-(1*H*-indol-3-yl)acetamide **3** (329 mg, 86% yield) as a reddish-brown oil: $[\alpha]_D^{20}$= +127.5 (c 0.5, MeOH). IR (KBr) Vmax:3441, 3281, 3052, 2962, 2914, 2878, 1645, 1573, 1537, 1452, 1362, 1259, 1061, 929, 748, 682 cm^{-1}. ^1H NMR (400 MHz, $CDCl_3$) δ 9.67 (s, 1H), 7.52 (d, J = 7.9 Hz, 1H), 7.33 (d, J = 8.1 Hz, 1H), 7.13 (q, J = 6.6, 6.0 Hz, 5H), 7.08 – 6.98 (m, 3H), 6.59 (t, J = 6.1 Hz, 1H), 5.19 – 5.15 (m, 1H), 5.05 (s, 1H), 4.30 (qd, J = 15.1, 5.8 Hz, 2H), 1.11 (s, 9H). ^{13}C NMR (100 MHz, $CDCl_3$) δ 171.2, 137.7, 137.2, 128.6, 127.4, 126.4, 125.2, 122.6, 119.9, 112.0, 110.0, 55.6, 54.7, 43.7, 22.7. HRMS (ESI) calculated for $C_{21}H_{26}N_3O_2S^+[M+H]^+$: 384.1740, found: 384.1742.

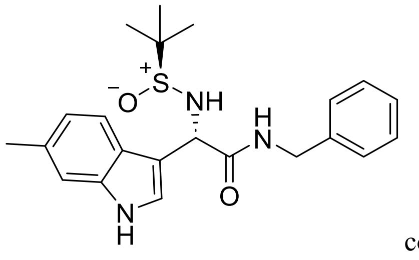

compound **4**

(*S*)-*N*-benzyl-2-(((*S*)-*tert*-butylsulfinyl)amino)-2-(6-methyl-1*H*-indol-3-yl)acetamide compound **4:** Prepared by using the representative procedure above from compound **2a** (0.10 g, 0.38 mmol) and 6-methylindole (**1b**). Purified by column chromatography on silica gel (petroleum ether:ethyl acetate=3:1) to afford compound **4** (134 mg, 90% yield) as a golden oil: $[\alpha]_D^{20}$= +74.30 (c 0.2, MeOH)**.** IR (KBr) Vmax: 3552, 3287, 2950, 2908, 2866, 1783, 1722, 1705, 1638, 1548, 1500, 1440, 1422, 1368, 1319, 1254, 1037, 905, 802, 748, 700, 598 cm^{-1}. ^1H NMR (400 MHz, $CDCl_3$) δ 8.49 – 8.40 (m, 1H), 7.45 (d, J = 8.1 Hz, 1H), 7.23 – 7.15 (m, 5H), 7.07 (dd, J = 7.2, 2.4 Hz, 2H), 6.93 (dd, J = 8.2, 1.4 Hz, 1H), 6.24 – 6.13 (m, 1H), 5.22 (d, J = 1.7 Hz, 1H), 5.02 (d, J = 1.8 Hz, 1H), 4.48 (dd, J = 15.0, 6.3 Hz, 1H), 4.30 (dd, J = 15.1, 5.7 Hz, 1H), 2.45 (s, 3H), 1.18 (s, 9H). ^{13}C NMR (100 MHz, $CDCl_3$) δ 170.8, 137.7, 137.5, 132.8, 128.6, 127.4, 125.4, 122.9, 122.0, 119.8, 111.6, 110.5, 55.4, 54.3, 43.8, 22.7, 21.7. HRMS (ESI) calculated for $C_{22}H_{28}N_3O_2S^+[M+H]^+$: 398.1897, found: 398.1899.

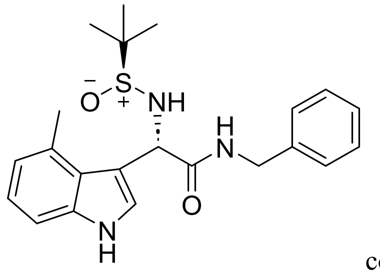

compound **5**

(*S*)-*N*-benzyl-2-(((*S*)-*tert*-butylsulfinyl)amino)-2-(4-methyl-1*H*-indol-3-yl)acetamide compound **5:** Prepared by using the representative procedure above from compound **2a** (0.10 g, 0.38 mmol) and 4-methylindole (**1c**). Purified by column chromatography on silica gel (petroleum ether:ethyl acetate=3:1) to afford compound **5** (137 mg, 92% yield) as a pale yellow oil: $[\alpha]_D^{20}$= +62.90 (c 0.1

MeOH). IR (KBr) Vmax: 3552, 3245, 3112, 3058, 2950, 2920, 2860, 1855, 1711, 1645, 1513, 1458, 1362, 1344, 1265, 1103, 1025, 905, 748, 694, 598 cm^{-1}. ^{1}H NMR (400 MHz, $CDCl_3$) δ 8.78 (s, 1H), 7.31 (d, J = 2.6 Hz, 1H), 7.23 (td, J = 5.0, 1.9 Hz, 3H), 7.16 – 7.04 (m, 3H), 6.89 (d, J = 7.1 Hz, 1H), 6.19 (s, 1H), 5.27 (s, 1H), 5.16 (s, 1H), 4.56 (dd, J = 14.9, 6.5 Hz, 1H), 4.26 (dd, J = 14.9, 5.5 Hz, 1H), 2.50 (s, 3H), 1.19 (s, 9H). ^{13}C NMR (100 MHz, $CDCl_3$) δ 171.7, 137.6, 131.0, 128.6, 127.6, 127.5, 124.4, 123.0, 122.1, 109.3, 55.3, 54.5, 43.8, 22.7, 20.8. HRMS (ESI) calculated for $C_{22}H_{28}N_3O_2S^+$[M+H]$^+$: 398.1898, found: 398.1897.

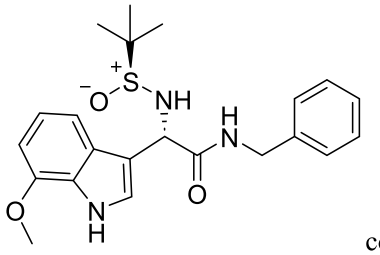

compound **6**

(*S*)-*N*-benzyl-2-(((*S*)-*tert*-butylsulfinyl)amino)-2-(7-methoxy-1*H*-indol-3-yl)acetamide compound **6:** Prepared by using the representative procedure above from compound **2a** (0.10 g, 0.38 mmol) and 7-Methoxy-1*H*-indole (**1d**). Purified by column chromatography on silica gel (petroleum ether:ethyl acetate=3:1) to afford compound **6** (134 mg, 86% yield) as a white solid: $[\alpha]_D^{20}$= + 96.67(c 0.3 MeOH). m.p. 115.6-117.4 °C. IR (KBr) Vmax: 3554, 3292, 2947, 2918, 2857, 2820, 1788, 1720, 1639, 1550, 1502, 1444, 1424, 1369, 1323, 1257, 1039, 903, 700, 598 cm^{-1}. ^{1}H NMR (400 MHz, $CDCl_3$) δ 8.77 (s, 1H), 7.29 (s, 1H), 7.25 – 7.18 (m, 4H), 7.12 – 7.08 (m, 2H), 7.04 (t, *J* = 7.9 Hz, 1H), 6.68 (d, *J* = 7.7 Hz, 1H), 6.25 (t, *J* = 6.0 Hz, 1H), 5.25 (d, *J* = 1.8 Hz, 1H), 5.05 (d, *J* = 1.8 Hz, 1H), 4.48 (dd, *J* = 15.1, 6.1 Hz, 1H), 4.34 (dd, *J* = 15.1, 5.8 Hz, 1H), 3.95 (s, 3H), 1.20 (s, 9H). ^{13}C NMR (100 MHz, $CDCl_3$) δ 170.8, 146.3, 137.7, 128.6, 127.6, 127.4, 126.5, 125.3, 120.7, 112.7, 111.2, 102.6, 55.4, 55.3, 54.2, 43.7, 22.7. HRMS (ESI) calculated for $C_{22}H_{28}N_3O_3S^+$[M+H]$^+$: 414.1846, found: 414.1847.

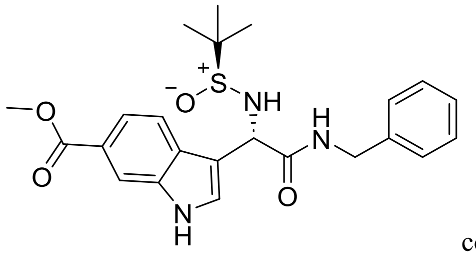

compound **7**

Methyl 3-((*S*)-2-(benzylamino)-1-(((*S*)-*tert*-butylsulfinyl)amino)-2-oxoethyl)-1*H*-indole-6-carboxy-late compound **7:** Prepared by using the representative procedure above from compound **2a** (0.10 g, 0.38 mmol) and methyl indole-6-carboxylate (**1e**). Purified by column chromatography on silica gel (petroleum ether:ethyl acetate=3:1) to afford compound **7** (104 mg, 63% yield) as a pale yellow oil: $[\alpha]_D^{20}$= - 1102.94 (c 0.1, MeOH). IR (KBr) Vmax: 3438, 2956, 2842, 1782, 1627, 1513, 1446, 1079, 1013, 872, 795 cm^{-1}. ^{1}H NMR (400 MHz, $CDCl_3$) δ 9.87 – 9.77 (m, 1H), 8.11 (d, J = 1.4 Hz, 1H), 7.73 (dd, J = 8.5, 1.4 Hz, 1H), 7.55 (d, J = 8.4 Hz, 1H), 7.37 (d, J = 2.6 Hz, 1H), 7.16 (dd, J = 5.2, 1.9 Hz, 3H), 7.04 (dd, J = 6.9, 2.7 Hz, 2H), 6.55 (t, J = 6.1 Hz, 1H), 5.21 (d, J = 2.7 Hz, 1H), 5.04 (d, J = 2.8 Hz, 1H), 4.35 (d, J = 6.0 Hz, 2H), 3.89 (s, 3H), 1.13 (s, 9H). ^{13}C NMR (100 MHz, $CDCl_3$) δ 170.7, 168.0, 137.5, 136.4, 129.4, 128.7, 128.6, 127.5, 127.4, 124.3, 120.9, 119.4, 114.4, 110.8, 55.7, 54.6, 52.0, 43.8, 22.7. HRMS (ESI) calculated for $C_{23}H_{28}N_3O_4S^+$[M+H]$^+$: 442.1795,

found: 442.1798.

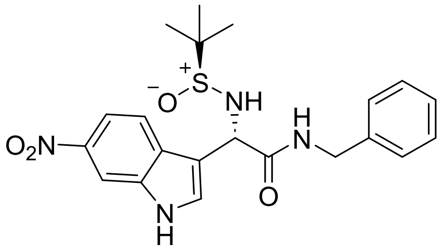

compound **8**

(*S*)-*N*-benzyl-2-(((*S*)-*tert*-butylsulfinyl)amino)-2-(6-nitro-1*H*-indol-3-yl)acetamide compound **8:** Prepared by using the representative procedure above from compound **2a** (0.10 g, 0.38 mmol) and 6-nitroindole (**1f**). Purified by column chromatography on silica gel (petroleum ether:ethyl acetate=3:1) to afford compound **8** (117 mg, 73% yield) as a yellow green solid powder: $[\alpha]_D^{20}$=+ 81.60(c 0.25, MeOH). m.p. 123.3-125.1 °C. IR (KBr) Vmax: 3264, 3070, 2955, 2345, 1659, 1528, 1366, 1242, 1173, 1042, 934, 849, 802, 702, 610 cm^{-1}. ^1H NMR (400 MHz, $CDCl_3$) δ 9.50 (s, 1H), 8.33 (d, *J* = 2.0 Hz, 1H), 7.97 (dd, *J* = 8.9, 2.0 Hz, 1H), 7.61 (d, *J* = 8.8 Hz, 1H), 7.53 (d, *J* = 2.6 Hz, 1H), 7.23 – 7.14 (m, 3H), 7.14 – 6.97 (m, 3H), 5.00 (s, 1H), 4.42 (dd, *J* = 9.6, 5.9 Hz, 2H), 1.19 (s, 9H). ^{13}C NMR (100 MHz, $CDCl_3$) δ 169.9, 143.9, 137.3, 135.5, 131.2, 129.8, 128.7, 127.7, 127.5, 120.0, 115.6, 108.7, 55.8, 54.1, 44.0, 22.7. HRMS (ESI) calculated for $C_{21}H_{25}N_4O_4S^+[M+H]^+$: 429.1591, found: 429.1593.

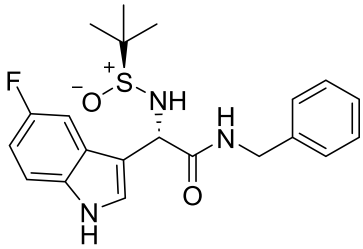

compound **9**

(*S*)-*N*-benzyl-2-(((*S*)-*tert*-butylsulfinyl)amino)-2-(5-fluoro-1*H*-indol-3-yl)acetamide compound **9:** Prepared by using the representative procedure above from compound **2a** (0.10 g, 0.38 mmol) and 5-Fluoroindole (**1g**). Purified by column chromatography on silica gel (petroleum ether:ethyl acetate=3:1) to afford compound **9** (131 mg, 87% yield) as a pale yellow oil: $[\alpha]_D^{20}$=+ 76.17(c 0.6, MeOH). IR (KBr) Vmax: 3279, 3063, 2955, 2924, 2345, 1659, 1528, 1489, 1458, 1358, 1242, 1173, 1049, 941, 794, 702 cm^{-1}. ^1H NMR (400 MHz, $CDCl_3$) δ 9.51 – 9.42 (m, 1H), 7.37 – 7.31 (m, 3H), 7.28 (dd, *J* = 4.7, 2.1 Hz, 2H), 7.24 (t, *J* = 2.4 Hz, 1H), 7.14 (dd, *J* = 7.4, 2.1 Hz, 2H), 6.99 (td, *J* = 9.0, 2.5 Hz, 1H), 6.46 (t, *J* = 6.0 Hz, 1H), 5.22 (d, *J* = 2.1 Hz, 1H), 5.07 (d, *J* = 2.1 Hz, 1H), 4.44 (dd, *J* = 8.1, 6.0 Hz, 2H), 1.23 (s, 9H). ^{13}C NMR (100 MHz, $CDCl_3$) δ 170.8, 146.3, 137.7, 128.6, 127.6, 127.4, 126.5, 125.3, 120.7, 112.7, 111.2, 102.6, 55.4, 55.3, 54.2, 43.7, 22.7. ^{19}F NMR (376 MHz, $CDCl_3$) δ -123.5. HRMS (ESI) calculated for $C_{21}H_{24}FN_3O_2S^+[M+H]^+$: 402.1647, found: 402.1648.

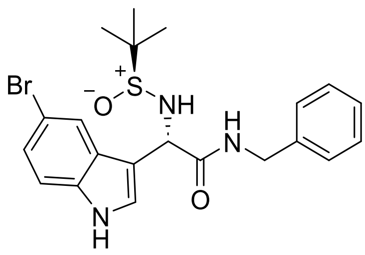

compound **10**

(*S*)-*N*-benzyl-2-(5-bromo-1*H*-indol-3-yl)-2-(((*S*)-*tert*-butylsulfinyl)amino)acetamide compound **10:**

Prepared by using the representative procedure above from compound **2a** (0.10 g, 0.38 mmol) and 5-bromoindole (**1h**). Purified by column chromatography on silica gel (petroleum ether:ethyl acetate=3:1) to afford compound **10** (142 mg, 82% yield) as a reddish-brown oil: $[\alpha]_D^{20}$=+77.25 (c 0.4, MeOH). IR (KBr) Vmax: 3286, 3256, 2932, 1666, 1535, 1458, 1366, 1250, 1042, 903, 810, 702, 594 cm^{-1}. ^1H NMR (400 MHz, $CDCl_3$) δ 9.23 (s, 1H), 7.71 (d, *J* = 1.8 Hz, 1H), 7.29 (d, *J* = 1.7 Hz, 1H), 7.25 – 7.19 (m, 4H), 7.16 (d, *J* = 2.5 Hz, 1H), 7.11 – 7.07 (m, 2H), 6.33 (t, *J* = 6.0 Hz, 1H), 5.14 (d, *J* = 2.1 Hz, 1H), 4.99 (s, 1H), 4.47 – 4.25 (m, 2H), 1.18 (s, 9H). ^{13}C NMR (100 MHz, $CDCl_3$) δ 170.4, 137.5, 135.7, 128.7, 127.5, 127.4, 127.3, 126.7, 125.8, 122.7, 113.5, 113.3, 109.9, 55.7, 54.4, 43.8, 22.7. HRMS (ESI) calculated for $C_{21}H_{25}BrN_3O_2S^+[M+H]^+$: 462.0845, found: 462.0847.

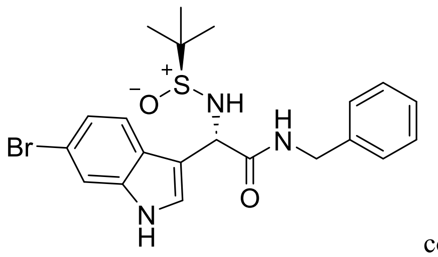

compound **11**

(*S*)-*N*-benzyl-2-(6-bromo-1*H*-indol-3-yl)-2-(((*S*)-*tert*-butylsulfinyl)amino)acetamide compound **11:**

Prepared by using the representative procedure above from compound **2a** (0.10 g, 0.38 mmol) and 6-bromoindole (**1i**). Purified by column chromatography on silica gel (petroleum ether:ethyl acetate=3:1) to afford compound **11** (144 mg, 83% yield) as a pale yellow oil: $[\alpha]_D^{20}$= -1096.23 (c 0.1, MeOH). IR (KBr) Vmax: 3452, 3263, 2956, 2920, 2842, 1657, 1645, 1614, 1554, 1524, 1458, 1326, 1235, 1025, 886, 802, 754, 688, 580 cm^{-1}. ^1H NMR (400 MHz, $CDCl_3$) δ 9.11 (s, 1H), 7.53 (d, J = 1.7 Hz, 1H), 7.40 (d, J = 8.5 Hz, 1H), 7.24 – 7.13 (m, 5H), 7.06 (dd, J = 7.2, 2.4 Hz, 2H), 6.29 (t, J = 5.9 Hz, 1H), 5.17 (d, J = 2.0 Hz, 1H), 5.00 (d, J = 2.1 Hz, 1H), 4.38 (qd, J = 15.0, 6.0 Hz, 2H), 1.17 (s, 9H). ^{13}C NMR (100 MHz, $CDCl_3$) δ 170.5, 137.9, 137.5, 128.6, 127.5, 127.4, 126.7, 123.9, 123.5, 121.2, 116.5, 114.8, 110.7, 55.6, 54.3, 43.8, 22.7. HRMS (ESI) calculated for $C_{21}H_{25}BrN_3O_2S^+[M+H]^+$: 462.0845, found: 462.0848.

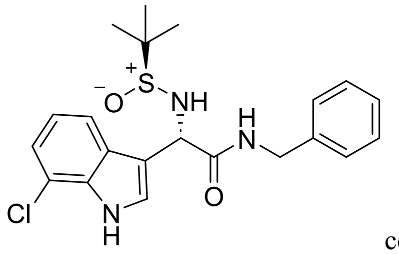

compound **12**

(*S*)-*N*-benzyl-2-(((*S*)-*tert*-butylsulfinyl)amino)-2-(7-chloro-1*H*-indol-3-yl)acetamide compound **12:**

Prepared by using the representative procedure above from compound **2a** (0.10 g, 0.38 mmol) and 7-Chloroindole (**1j**). Purified by column chromatography on silica gel (petroleum ether:ethyl acetate=3:1) to afford compound **12** (124 mg, 79% yield) as a pale yellow solid: $[\alpha]_D^{20}$= +258.25 (c 0.1, MeOH). m.p. 105.2-107.1 °C. IR (KBr) Vmax: 3287, 3256, 2924, 1659, 1543, 1458, 1412, 1250, 1049, 903, 802, 702, 594 cm^{-1}. ^1H NMR (400 MHz, $CDCl_3$) δ 8.97 (s, 1H), 7.39 (d, *J* = 7.9

Hz, 1H), 7.26 (d, *J* = 2.5 Hz, 1H), 7.15 – 7.10 (m, 4H), 6.99 (dd, *J* = 6.9, 2.5 Hz, 2H), 6.94 (t, *J* = 7.8 Hz, 1H), 6.24 (t, *J* = 5.9 Hz, 1H), 5.14 (d, *J* = 2.0 Hz, 1H), 4.95 (d, *J* = 2.0 Hz, 1H), 4.30 (qd, *J* = 15.0, 5.9 Hz, 2H), 1.08 (s, 9H). ^{13}C NMR (100 MHz, $CDCl_3$) δ 170.3, 137.5, 134.2, 128.5, 127.4, 127.3, 126.5, 126.5, 122.2, 120.9, 118.7, 117.0, 111.9, 55.5, 54.1, 43.7, 22.6. HRMS (ESI) calculated for $C_{21}H_{25}ClN_3O_2S^+$[M+H]$^+$: 418.1351, found: 418.1354.

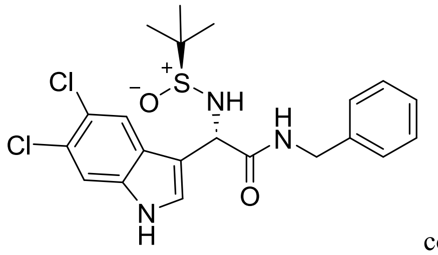

compound **13**

(*S*)-*N*-benzyl-2-(((*S*)-*tert*-butylsulfinyl)amino)-2-(5,6-dichloro-1*H*-indol-3-yl)acetamide

compound **13:** Prepared by using the representative procedure above from compound **2a** (0.10 g, 0.38 mmol) and 5,6-dichloroindole (**1k**). Purified by column chromatography on silica gel (petroleum ether:ethyl acetate=3:1) to afford compound **13** (124 mg, 79% yield) as a pale yellow solid: $[\alpha]_D^{20}$= +51.00 (c 0.4, MeOH). m.p. 102.9-104.1 °C. IR (KBr) Vmax: 3742, 3672, 3611, 3318, 3233, 1666, 1520, 1450, 1312, 1042, 702 cm^{-1}. ^{1}H NMR (400 MHz, $CDCl_3$) δ 9.31 (s, 1H), 7.54 (s, 1H), 7.36 (s, 1H), 7.20 – 7.12 (m, 3H), 7.06 (s, 1H), 7.03 – 6.99 (m, 2H), 6.31 (t, *J* = 6.0 Hz, 1H), 5.03 (s, 1H), 4.91 (s, 1H), 4.32 – 4.28 (m, 2H), 1.10 (s, 9H). ^{13}C NMR (100 MHz, $CDCl_3$) δ 170.3, 137.4, 135.8, 128.7, 128.1, 127.4, 126.8, 124.7, 120.9, 113.4, 109.9, 55.8, 54.3, 43.9, 22.7. HRMS (ESI) calculated for $C_{20}H_{22}Cl_2N_3O_2S^+$[M+H]$^+$: 451.0888, found: 451.0890.

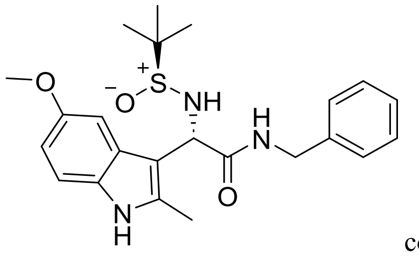

compound **14**

(*S*)-*N*-benzyl-2-(((*S*)-*tert*-butylsulfinyl)amino)-2-(5-methoxy-2-methyl-1*H*-indol-3-yl)acetamide

compound **14:** Prepared by using the representative procedure above from compound **2a** (0.10 g, 0.38 mmol) and 5-methoxy-2-methylindole (**1l**). Purified by column chromatography on silica gel (petroleum ether:ethyl acetate=3:1) to afford compound **14** (193 mg, 87% yield) as a pale yellow oil: $[\alpha]_D^{20}$= +82.22 (c 0.2, MeOH). IR (KBr) Vmax: 3317, 3209, 3041, 2944, 2848, 2757, 1645, 1524, 1446, 1416, 1410, 1362, 1265, 1194, 1103, 1079, 1013, 965, 941, 869, 778, 736, 712, 646, 586, 550 cm^{-1}. ^{1}H NMR (400 MHz, $CDCl_3$) δ 9.01 (s, 1H), 7.14 (d, J = 8.7 Hz, 1H), 7.09 – 7.04 (m, 2H), 6.87 (d, J = 2.6 Hz, 1H), 6.74 (dd, J = 8.8, 2.4 Hz, 1H), 6.35 (t, J = 6.1 Hz, 1H), 5.18 (s, 1H), 5.00 (s, 1H), 4.52 – 4.20 (m, 2H), 3.67 (s, 3H), 2.31 (s, 3H), 1.16 (s, 9H). ^{13}C NMR (100 MHz, $CDCl_3$) δ 171.1, 154.1, 137.8, 137.0, 130.7, 128.6, 127.4, 127.4, 126.9, 111.7, 111.6, 105.1, 100.9, 55.7, 55.2, 52.8, 43.7, 22.7, 22.1, 11.7. HRMS (ESI) calculated for $C_{23}H_{30}N_3O_3S^+$[M+H]$^+$: 428.2002, found: 428.2004.

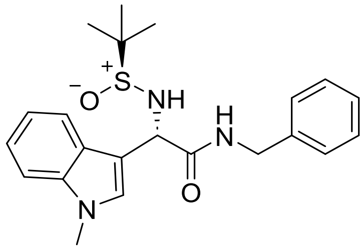

compound **15**

(*S*)-*N*-benzyl-2-(((*S*)-*tert*-butylsulfinyl)amino)-2-(1-methyl-1*H*-indol-3-yl)acetamide compound **15:**

Prepared by using the representative procedure above from compound **2a** (0.10 g, 0.38 mmol) and 1-methylindole (**1m**). Purified by column chromatography on silica gel (petroleum ether:ethyl acetate=3:1) to afford compound **15** (139 mg, 93% yield) as a pale yellow oil: $[\alpha]_D^{20}$= -511 (c 0.2, MeOH). IR (KBr) Vmax: 3419, 3293, 3058, 2968, 2908, 2854, 1651, 1560, 1518, 1464, 1404, 1344, 1326, 1235, 1151, 1049, 892, 808, 736, 694, 586 cm^{-1}. ^{1}H NMR (400 MHz, $CDCl_3$) δ 7.57 (d, J = 8.0 Hz, 1H), 7.31 (d, J = 8.2 Hz, 1H), 7.25 (d, J = 2.3 Hz, 1H), 7.23 – 7.17 (m, 3H), 7.16 (s, 1H), 7.13 – 7.05 (m, 3H), 6.30 (t, J = 6.0 Hz, 1H), 5.22 (d, J = 2.1 Hz, 1H), 5.02 (d, J = 2.1 Hz, 1H), 4.45 (dd, J = 15.0, 6.1 Hz, 1H), 4.31 (dd, J = 15.0, 5.9 Hz, 1H), 3.75 (s, 3H), 1.16 (s, 9H). ^{13}C NMR (100 MHz, $CDCl_3$) δ 170.9, 137.8, 137.7, 130.3, 128.6, 127.4, 127.4, 125.7, 122.5, 120.3, 119.9, 109.7, 109.2, 55.4, 54.2, 43.7, 32.9, 22.7. HRMS (ESI) calculated for $C_{22}H_{28}N_3O_2S^+[M+H]^+$: 398.1897, found: 398.1899.

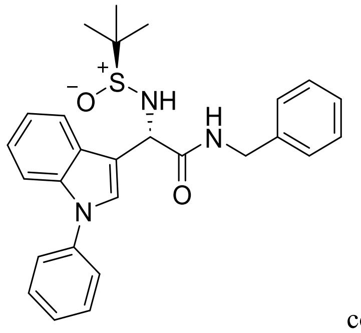

compound **16**

(*S*)-*N*-benzyl-2-(((*S*)-*tert*-butylsulfinyl)amino)-2-(1-phenyl-1*H*-indol-3-yl)acetamide compound **16:**

Prepared by using the representative procedure above from compound **2a** (0.10 g, 0.38 mmol) and 1-phenyl-1*H*-indole (**1n**). Purified by column chromatography on silica gel (petroleum ether:ethyl acetate=3:1) to afford compound **16** (141 mg, 82% yield) as a white solid: $[\alpha]_D^{20}$= +47.0 (c 0.2, MeOH). m.p. 123.3-124.2 °C. IR (KBr) Vmax: 2971, 2854, 1651, 1560, 1518, 1464, 1404, 1344, 1326, 1235, 1151, 1049, 892, 808, 736, 694, 586 cm^{-1}. ^{1}H NMR (400 MHz, $CDCl_3$) δ 7.55 (d, *J* = 7.9 Hz, 1H), 7.47 (d, *J* = 8.2 Hz, 1H), 7.41 – 7.37 (m, 4H), 7.17 – 7.11 (m, 6H), 7.07 (d, *J* = 7.6 Hz, 1H), 7.02 (dd, *J* = 7.4, 1.9 Hz, 2H), 6.24 (t, *J* = 6.1 Hz, 1H), 5.22 (d, *J* = 2.1 Hz, 1H), 4.96 (d, *J* = 2.2 Hz, 1H), 4.38 (d, *J* = 5.9 Hz, 1H), 4.29 (d, *J* = 5.8 Hz, 1H), 1.10 (s, 9H). ^{13}C NMR (100 MHz, $CDCl_3$) δ 170.4, 139.0, 137.7, 136.9, 129.7, 129.0, 128.6, 127.5, 126.9, 126.5, 124.3, 123.3, 120.9, 120.4, 112.2, 111.0, 55.6, 54.2, 43.8, 22.7. HRMS (ESI) calculated for $C_{27}H_{30}N_3O_2S^+[M+H]^+$: 460.2053, found: 460.2055.

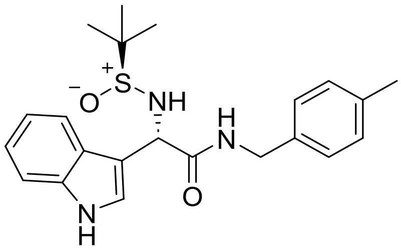

compound **19**

(*S*)-2-(((*S*)-*tert*-butylsulfinyl)amino)-2-(1*H*-indol-3-yl)-*N*-(4-methylbenzyl)acetamide compound **19** (282 mg, 88% yield) as a reddish-brown oil: $[\alpha]_D^{20}$= +46.7 (c 0.2, MeOH). IR (KBr) Vmax: 3453, 3122, 2920, 1855, 1711, 1645, 1458, 1362, 1265, 1103, 1025, 901, 751, 598 cm^{-1}. ^{1}H NMR (400 MHz, $CDCl_3$) δ 8.78 – 8.69 (m, 1H), 7.57 (d, J = 8.0 Hz, 1H), 7.38 (d, J = 8.2 Hz, 1H), 7.25 (d, J = 3.2 Hz, 1H), 7.21 (t, J = 7.6 Hz, 1H), 7.09 (t, J = 7.5 Hz, 1H), 7.02 (d, J = 7.9 Hz, 2H), 6.95 (d, J = 7.9 Hz, 2H), 6.19 (t, J = 6.0 Hz, 1H), 5.22 (d, J = 1.9 Hz, 1H), 5.02 (s, 1H), 4.42 (dd, J = 14.9, 6.0 Hz, 1H), 4.28 (dd, J = 14.9, 5.7 Hz, 1H), 2.27 (s, 3H), 1.17 (s, 9H). ^{13}C NMR (100 MHz, $CDCl_3$) δ 170.7, 137.1, 137.0, 134.6, 129.2, 127.4, 125.9, 125.2, 122.8, 120.2, 120.1, 111.7, 110.7, 55.5, 54.3, 43.6, 22.7, 22.1. HRMS (ESI) calculated for $C_{22}H_{28}N_3O_2S^+[M+H]^+$: 398.1897, found: 398.1899.

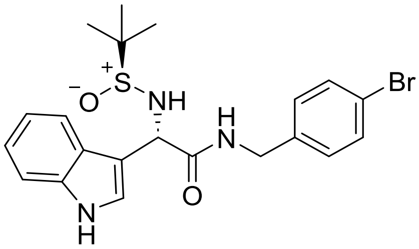

compound **20**

(*S*)-*N*-(4-bromobenzyl)-2-(((*S*)-*tert*-butylsulfinyl)amino)-2-(1*H*-indol-3-yl)acetamide compound **20:** Prepared by using the representative procedure above from compound **1a** (0.10 g, 0.85 mmol) and (*S,E*)-*N*-(4-bromobenzyl)-2-((*tert*-butylsulfinyl)imino)acetamide (**2c**). Purified by column chromatography on silica gel (petroleum ether:ethyl acetate=3:1) to afford compound **20** (270 mg, 82% yield) as a reddish-brown oil: $[\alpha]_D^{20}$= +67.8 (c 0.2, MeOH). IR(KBr)Vmax: 3252, 2981, 2928, 1649, 1509, 1455, 1362, 1224, 1158, 1049, 1012, 821, 736, 568 cm^{-1}. ^{1}H NMR (400 MHz, $CDCl_3$) δ 9.39 – 9.27 (m, 1H), 7.51 (d, J = 8.0 Hz, 1H), 7.36 (d, J = 8.2 Hz, 1H), 7.29 (d, J = 8.2 Hz, 2H), 7.21 – 7.15 (m, 2H), 7.07 (t, J = 7.5 Hz, 1H), 6.91 (d, J = 8.2 Hz, 2H), 6.54 (t, J = 6.1 Hz, 1H), 5.19 (d, J = 2.3 Hz, 1H), 4.99 (d, J = 2.3 Hz, 1H), 4.28 (t, J = 5.9 Hz, 2H), 1.15 (s, 9H). ^{13}C NMR (100 MHz, $CDCl_3$) δ 171.1, 137.1, 136.8, 131.6, 129.1, 126.1, 125.1, 122.7, 121.2, 120.1, 119.9, 111.9, 110.3, 55.6, 54.6, 43.1, 22.7, 22.1. HRMS (ESI) calculated for $C_{21}H_{25}BrN_3O_2S^+[M+H]^+$: 462.0845, found: 462.0848.

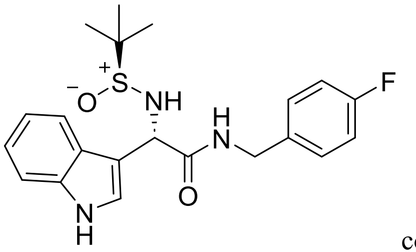

compound **21**

(*S*)-2-(((*S*)-*tert*-butylsulfinyl)amino)-*N*-(4-fluorobenzyl)-2-(1*H*-indol-3-yl)acetamide compound

21:

Prepared by using the representative procedure above from compound **1a** (0.10 g, 0.85 mmol) and (*S,E*)-2-((*tert*-butylsulfinyl)imino)-*N*-(4-fluorobenzyl)acetamide (**2d**). Purified by column chromatography on silica gel (petroleum ether:ethyl acetate=3:1) to afford compound **21** (225 mg, 79% yield) as a reddish-brown oil: $[\alpha]_D^{20}$= +72.5 (c 0.2, MeOH). IR (KBr) Vmax: 3251, 3065, 2981, 2926, 1657, 1627, 1537, 1506, 1452, 1410, 1362, 1338, 1224, 1157, 1091, 1049, 1007, 821, 736, 568 cm^{-1}. ^1H NMR (400 MHz, $CDCl_3$) δ 9.72 (s, 1H), 7.50 (d, J = 8.0 Hz, 1H), 7.34 (d, J = 8.2 Hz, 1H), 7.19 – 7.10 (m, 2H), 7.03 (ddd, J = 8.0, 7.0, 1.0 Hz, 1H), 7.00 – 6.93 (m, 2H), 6.83 – 6.77 (m, 2H), 6.76 – 6.70 (m, 1H), 5.17 (d, J = 2.7 Hz, 1H), 5.02 (d, J = 2.7 Hz, 1H), 4.39 – 4.08 (m, 2H), 1.11 (s, 9H). ^{13}C NMR (100 MHz, $CDCl_3$) δ 171.2, 163.2, 160.8, 137.2, 133.6, 133.5, 129.1, 129.0, 126.3, 125.1, 122.6, 119.9, 119.8, 115.4, 115.2, 112.0, 110.1, 55.6, 54.8, 43.0, 22.7. HRMS (ESI) calculated for $C_{21}H_{25}FN_3O_2S^+[M+H]^+$: 402.1646, found: 402.1648.

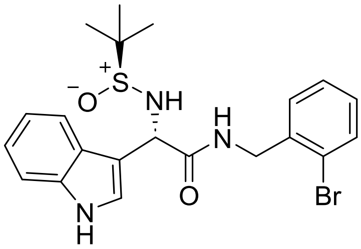

compound **22**

(*S*)-*N*-(2-bromobenzyl)-2-(((*S*)-*tert*-butylsulfinyl)amino)-2-(1*H*-indol-3-yl)acetamide compound **22:** Prepared by using the representative procedure above from compound **1a** (0.10 g, 0.85 mmol) and (*S,E*)-*N*-(2-bromobenzyl)-2-((*tert*-butylsulfinyl)imino)acetamide (**2e**). Purified by column chromatography on silica gel (petroleum ether:ethyl acetate=3:1) to afford compound **22** (286 mg, 87% yield) as a reddish-brown oil: $[\alpha]_D^{20}$= +120.5 (c 0.2, MeOH). IR (KBr) Vmax: 3287, 3065, 2963, 2908, 2848, 1663, 1543, 1500, 1434, 1404, 1326, 1265, 1067, 1019, 718, 652 cm^{-1}. ^1H NMR (400 MHz, $CDCl_3$) δ 9.01 (s, 1H), 7.50 (dd, J = 8.0, 2.6 Hz, 1H), 7.40 (ddd, J = 18.7, 8.0, 2.7 Hz, 2H), 7.23 – 7.16 (m, 2H), 7.14 (t, J = 3.6 Hz, 2H), 7.05 (tq, J = 7.3, 3.4, 2.8 Hz, 2H), 6.52 – 6.41 (m, 1H), 5.22 (d, J = 2.5 Hz, 1H), 4.98 (d, J = 2.6 Hz, 1H), 4.43 (dd, J = 6.2, 2.6 Hz, 2H), 1.17 (d, J = 2.7 Hz, 9H). ^{13}C NMR (100 MHz, $CDCl_3$) δ 171.0, 137.1, 136.6, 132.7, 129.6, 129.1, 127.5, 126.2, 125.1, 123.4, 122.7, 120.1, 120.0, 111.8, 110.2, 55.5, 54.4, 44.1, 29.7, 22.7. HRMS (ESI) calculated for $C_{21}H_{25}BrN_3O_2S^+[M+H]^+$: 462.0845, found: 462.0848.

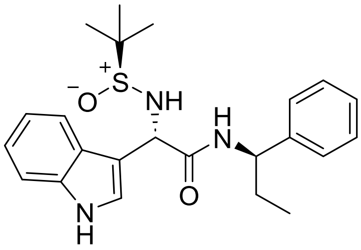

compound **23**

(*S*)-2-(((*S*)-*tert*-butylsulfinyl)amino)-2-(1*H*-indol-3-yl)-*N*-((*R*)-1-phenylpropyl)acetamide compound **23:** Prepared by using the representative procedure above from compound **1a** (0.10 g, 0.85 mmol) and (*E*)-2-(((*S*)-*tert*-butylsulfinyl)imino)-*N*-((*R*)-1-phenylpropyl)acetamide (**2f**). Purified by column chromatography on silica gel (petroleum ether:ethyl acetate=3:1) to afford compound **23** (260 mg, 89% yield) as a milky white oil: $[\alpha]_D^{20}$= +115 (c 0.2, MeOH). IR (KBr) Vmax: 3317, 3263, 3197, 3079, 2962, 2872, 1651, 1560, 1452, 1350, 1205, 1049, 754, 688 cm^{-1}.

^{1}H NMR (400 MHz, $CDCl_3$) δ 8.77 (s, 1H), 7.56 (d, J = 8.0 Hz, 1H), 7.40 (d, J = 8.2 Hz, 1H), 7.29 (d, J = 6.8 Hz, 1H), 7.25 – 7.19 (m, 3H), 7.18 – 7.13 (m, 2H), 7.09 (t, J = 7.5 Hz, 1H), 6.22 (d, J = 8.2 Hz, 1H), 5.16 (d, J = 2.1 Hz, 1H), 4.95 (s, 1H), 4.84 (q, J = 7.6 Hz, 1H), 1.67 – 1.53 (m, 2H), 1.17 (s, 9H), 0.62 (t, J = 7.4 Hz, 3H). ^{13}C NMR (100 MHz, $CDCl_3$) δ 170.1, 141.7, 137.0, 128.6, 127.4, 126.5, 126.0, 125.1, 122.8, 120.2, 120.0, 111.7, 110.8, 55.5, 54.5, 28.8, 22.7, 10.4. HRMS (ESI) calculated for $C_{23}H_{30}N_3O_2S^+[M+H]^+$: 412.2053, found: 412.2056.

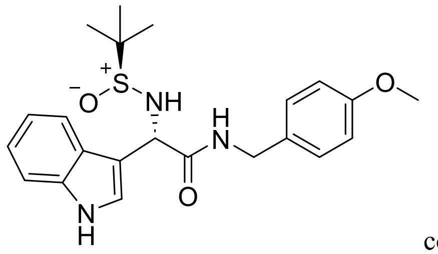

compound **24**

(*S*)-2-(((*S*)-*tert*-butylsulfinyl)amino)-2-(1*H*-indol-3-yl)-*N*-(4-methoxybenzyl)acetamide compound **24:** Prepared by using the representative procedure above from compound **1a** (0.10 g, 0.85 mmol) and (*S,E*)-2-((*tert*-butylsulfinyl)imino)-*N*-(4-methoxybenzyl)acetamide (**2g**). Purified by column chromatography on silica gel (petroleum ether:ethyl acetate=3:1) to afford compound **24** (267 mg, 91% yield) as a yellow-brown solid: $[\alpha]_D^{20}$= +131 (c 0.2, MeOH). m.p. 107.1-109.0 °C. IR (KBr) Vmax: 3414, 3275, 3071, 2981, 2914, 2842, 1711, 1687, 1638, 1543, 1513, 1428, 1368, 1272, 1175, 1043, 808, 730 cm^{-1}. ^{1}H NMR (400 MHz, $CDCl_3$) δ 8.84 (s, 1H), 7.55 (d, J = 8.0 Hz, 1H), 7.38 (d, J = 8.2 Hz, 1H), 7.24 – 7.14 (m, 2H), 7.09 (t, J = 7.6 Hz, 1H), 7.02 – 6.96 (m, 2H), 6.78 – 6.69 (m, 2H), 6.22 (t, J = 5.9 Hz, 1H), 5.20 (d, J = 1.9 Hz, 1H), 5.03 (d, J = 2.0 Hz, 1H), 4.39 (dd, J = 14.8, 6.0 Hz, 1H), 4.26 (dd, J = 14.8, 5.7 Hz, 1H), 3.74 (s, 3H), 1.17 (s, 9H). ^{13}C NMR (100 MHz, $CDCl_3$) δ 170.6, 158.9, 137.0, 129.8, 128.8, 126.0, 125.1, 122.8, 120.1, 114.0, 111.7, 110.6, 55.3, 54.4, 43.3, 22.7. HRMS (ESI) calculated for $C_{22}H_{28}N_3O_3S^+[M+H]^+$: 414.1846, found: 414.1850.

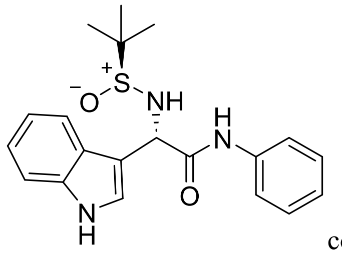

compound **25**

(*S*)-2-(((*S*)-*tert*-butylsulfinyl)amino)-2-(1*H*-indol-3-yl)-*N*-phenylacetamide compound **25:** Prepared by using the representative procedure above from compound **1a** (0.10 g, 0.85 mmol) and (*S,E*)-2-((*tert*-butylsulfinyl)imino)-*N*-phenylacetamide (**2h**). Purified by column chromatography on silica gel (petroleum ether:ethyl acetate=3:1) to afford compound **25** (234 mg, 89% yield) as a yellow-brown oil: $[\alpha]_D^{20}$= +180.91 (c 0.1, MeOH). IR (KBr) Vmax: 3428, 3251, 3071, 2932, 2848, 1692, 1603, 1548, 1518, 1358, 1319, 1254, 1164, 1049, 899, 760, 694 cm^{-1}. ^{1}H NMR (400 MHz, $CDCl_3$) δ 9.35 (s, 1H), 8.30 (dd, J = 7.2, 4.0 Hz, 1H), 7.59 (d, J = 8.0 Hz, 1H), 7.36 (t, J = 7.4 Hz, 3H), 7.22 – 7.12 (m, 4H), 7.08 – 6.95 (m, 2H), 5.32 (d, J = 3.3 Hz, 1H), 5.02 (s, 1H), 1.18 (s, 9H). ^{13}C NMR (100 MHz, $CDCl_3$) δ 169.4, 137.3, 137.1, 128.9, 126.1, 125.2, 124.7, 122.7, 120.3, 120.2, 119.7, 112.0, 110.3, 60.5, 55.8, 22.7. HRMS (ESI) calculated for $C_{20}H_{24}N_3O_2S^+[M+H]^+$: 370.1584, found: 370.1587.

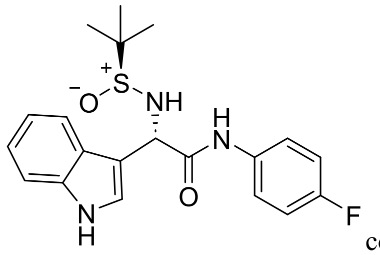
compound **26**

(*S*)-2-(((*S*)-*tert*-butylsulfinyl)amino)-*N*-(4-fluorophenyl)-2-(1*H*-indol-3-yl)acetamide compound **26:**

Prepared by using the representative procedure above from compound **1a** (0.10 g, 0.85 mmol) and (*S,E*)-2-((*tert*-butylsulfinyl)imino)-*N*-(4-fluorophenyl)acetamide (**2i**). Purified by column chromatography on silica gel (petroleum ether:ethyl acetate=3:1) to afford compound **26** (234 mg, 87% yield) as a reddish-brown oil: $[\alpha]_D^{20}$= +86.70 (c 0.2, MeOH). IR (KBr) Vmax: 2981, 1767, 1685, 1643, 1547, 1368, 1052, 667 cm^{-1}. ^1H NMR (400 MHz, $CDCl_3$) δ 9.21 (t, J = 8.8 Hz, 1H), 8.57 (t, J = 7.9 Hz, 1H), 7.61 (d, J = 8.0 Hz, 1H), 7.35 (d, J = 8.2 Hz, 1H), 7.33 – 7.29 (m, 2H), 7.20 – 7.15 (m, 2H), 7.08 (t, J = 7.5 Hz, 1H), 6.84 (t, J = 8.5 Hz, 2H), 5.35 (d, J = 4.0 Hz, 1H), 5.02 (d, J = 3.7 Hz, 1H), 1.18 (s, 9H). ^{13}C NMR (100 MHz, $CDCl_3$) δ 169.4, 160.7, 158.3, 137.0, 133.4, 125.7, 125.3, 122.8, 122.0, 121.9, 120.3, 119.6, 115.5, 115.3, 111.9, 110.7, 56.0, 55.8, 22.7. ^{19}F NMR (376 MHz, $CDCl_3$) δ -117.34. HRMS (ESI) calculated for $C_{20}H_{23}FN_3O_2S^+[M+H]^+$: 388.1490, found: 388.1494.

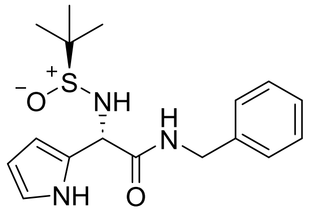
compound **27**

(*S*)-*N*-benzyl-2-(((*S*)-*tert*-butylsulfinyl)amino)-2-(1*H*-pyrrol-2-yl)acetamide compound **27** (377 mg, 91% yield) as a white solid: $[\alpha]_D^{20}$= +66.33 (c 0.2 MeOH). m.p. 131.8-133.1 °C. IR (KBr) Vmax: 3575, 3365, 3287, 3076, 2981, 2926, 1651, 1560, 1446, 1422, 1350, 1326, 1259, 1211, 1115, 1047, 916, 808, 730, 622, 598 cm^{-1}. ^1H NMR (400 MHz, $CDCl_3$) δ 9.42 (s, 1H), 7.29 (d, J = 8.1 Hz, 1H), 7.23 (t, J = 7.3 Hz, 1H), 7.18 – 7.12 (m, 3H), 6.75 (q, J = 2.2 Hz, 1H), 6.17 (dt, J = 14.4, 3.1 Hz, 2H), 5.14 (d, J = 4.6 Hz, 1H), 4.81 (d, J = 4.6 Hz, 1H), 4.38 (q, J = 9.1, 7.6 Hz, 2H), 1.97 (s, 1H), 1.23 (s, 9H). ^{13}C NMR (100 MHz, $CDCl_3$) δ 170.2, 137.6, 128.7, 127.5, 127.1, 119.0, 108.6, 107.9, 56.3, 55.7, 43.8, 22.6. HRMS (ESI) calculated for $C_{17}H_{24}N_3O_2S^+[M+H]^+$: 334.1584, found: 334.1585.

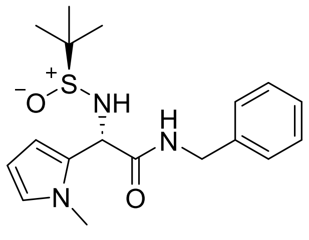
compound **28**

(*S*)-*N*-benzyl-2-(((*S*)-*tert*-butylsulfinyl)amino)-2-(1-methyl-1*H*-pyrrol-2-yl)acetamide compound **28:** Prepared by using the representative procedure above from 1-methyl-1*H*-pyrrole **3b** and

compound **2a** (0.10 g, 0.37mmol). Purified by column chromatography on silica gel (petroleum ether:ethyl acetate=3:1) to afford compound **28** (109 mg, 84% yield) as a white solid: $[\alpha]_D^{20}$= +46.33 (c 0.2, MeOH). m.p. 142.7-144.3 °C. IR (KBr) Vmax: 3257, 3071, 2956, 2901, 2842, 1657, 1548, 1446, 1380, 1302, 1229, 1181, 1037, 754, 706, 598 cm^{-1}. ^1H NMR (400 MHz, $CDCl_3$) δ 7.31 (d, *J* = 7.1 Hz, 2H), 7.29 (s, 1H), 7.25 – 7.23 (m, 1H), 7.18 (s, 1H), 7.16 (s, 1H), 6.63 (dt, *J* = 4.5, 2.1 Hz, 1H), 6.26 (dd, *J* = 3.5, 1.8 Hz, 1H), 5.10 (d, *J* = 1.7 Hz, 1H), 4.93 – 4.90 (m, 1H), 4.42 (t, *J* = 5.5 Hz, 2H), 3.62 (s, 1H), 3.47 (s, 3H), 1.22 (s, 9H). ^{13}C NMR (100 MHz, $CDCl_3$) δ 169.6, 137.7, 128.8, 127.7, 127.6, 125.3, 124.3, 112.6, 107.4, 55.6, 54.0, 43.9, 34.3, 22.6. HRMS (ESI) calculated for $C_{18}H_{26}N_3O_2S^+[M+H]^+$: 348.1740, found: 348.1745.

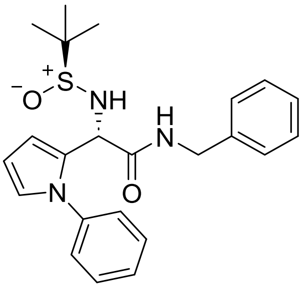

compound **29**

(*S*)-*N*-benzyl-2-(((*S*)-*tert*-butylsulfinyl)amino)-2-(1-phenyl-1*H*-pyrrol-2-yl)acetamide compound **29:** Prepared by using the representative procedure above from 1-pheny-1*H*-pyrrole **3c** and compound **2a** (0.10g, 0.37mmol). Purified by column chromatography on silica gel (petroleum ether:ethyl acetate=3:1) to afford compound **29** (134 mg, 88% yield) as a white solid: $[\alpha]_D^{20}$=+47.0 (c 0.2, MeOH). m.p. 136.8-137.6 °C. IR (KBr) Vmax: 3252, 3078, 2962, 2904, 2844, 1658, 1555, 1469, 1390, 1352, 1329, 1302, 1232, 1188, 1042, 754, 706, 598 cm^{-1}. ^1H NMR (400 MHz, $CDCl_3$) δ 7.45 – 7.27 (m, 8H), 7.25 (d, *J* = 7.4 Hz, 3H), 6.31 (s, 1H), 4.81 (d, *J* = 3.5 Hz, 1H), 4.39 (d, *J* = 5.8 Hz, 2H), 4.05 (d, *J* = 3.6 Hz, 1H), 1.82 (s, 1H), 1.10 (s, 9H). ^{13}C NMR (100 MHz, $CDCl_3$) δ 169.6, 137.8, 135.7, 130.7, 129.6, 129.5, 129.3, 128.7, 127.9, 127.5, 109.8, 56.4, 55.5, 43.8, 22.6. HRMS (ESI) calculated for $C_{23}H_{28}N_3O_2S^+[M+H]^+$: 410.1897, found: 410.1899.

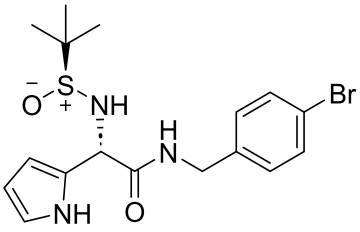

compound **31**

(*S*)-*N*-(4-bromobenzyl)-2-(((*S*)-*tert*-butylsulfinyl)amino)-2-(1*H*-pyrrol-2-yl)acetamide compound **31:** Prepared by using the representative procedure above from (*S,E*)-*N*-(4-bromobenzyl)-2-((*tert*-butylsulfinyl)imino)acetamide **2c** and compound **3a** (0.10g, 1.5mmol). Purified by column chromatography on silica gel (DCM:MeOH =20:1) to afford compound **31** (444 mg, 89% yield) as a white solid: $[\alpha]_D^{20}$= +62.5 (c 0.1, MeOH). m.p. 116.2-117.5 °C. IR (KBr) Vmax: 3733, 3636, 3558, 2981, 1705, 1663, 1608, 1567, 1530, 1513, 1452, 1410. 1362, 1037, 664 cm^{-1}. ^1H NMR (400 MHz, $CDCl_3$) δ 9.48 (s, 1H), 7.39 (d, J = 8.0 Hz, 2H), 7.02 (d, J = 8.0 Hz, 2H), 6.76 (s, 1H), 6.19 – 6.09 (m, 2H), 5.29 (s, 1H), 5.14 (d, J = 4.9 Hz, 1H), 4.82 (d, J = 5.0 Hz, 1H), 4.32 (q, J = 7.4, 5.6 Hz, 2H), 1.23 (s, 9H). ^{13}C NMR (100 MHz, $CDCl_3$) δ 170.4, 136.7, 131.8, 129.2, 127.1, 121.3, 119.0, 108.7, 107.8, 56.4, 55.9, 53.5, 31.9, 29.7, 22.6. HRMS (ESI) calculated for $C_{17}H_{23}BrN_3O_2S^+[M+H]^+$: 412.0689, found: 412.0692.

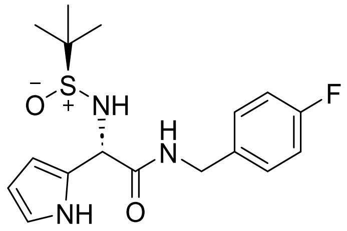 compound **32**

(*S*)-2-(((*S*)-*tert*-butylsulfinyl)amino)-*N*-(4-fluorobenzyl)-2-(1*H*-pyrrol-2-yl)acetamide compound **32:** Prepared by using the representative procedure above from (*S,E*)-2-((*tert*-butylsulfinyl)imino)-*N*-(4-fluorobenzyl)acetamide **2d** and compound **3a** (0.10g, 1.5mmol). Purified by column chromatography on silica gel (DCM:MeOH =20:1) to afford compound **32** (381 mg, 87% yield) as a white solid: $[\alpha]_D^{20}$=+49.0 (c 0.1, MeOH). m.p. 109.9-111.3 °C. IR (KBr) Vmax: 3323, 3245, 3058, 2902, 2842, 1645, 1500, 1446, 1368, 1319, 1224, 1157, 1037, 929, 808, 712 cm^{-1}. ^1H NMR (400 MHz, $CDCl_3$) δ 9.35 (s, 1H), 7.22 – 7.11 (m, 2H), 7.02 – 6.89 (m, 3H), 6.78 (q, J = 2.3 Hz, 1H), 6.20 – 6.12 (m, 2H), 5.13 (d, J = 5.1 Hz, 1H), 4.73 (t, J = 4.1 Hz, 1H), 4.36 (dd, J = 5.7, 2.6 Hz, 2H), 1.25 (s, 9H). ^{13}C NMR (100 MHz, $CDCl_3$) δ 170.3, 160.9, 129.3, 129.2, 127.0, 119.1, 115.7, 115.4, 108.7, 107.9, 56.4, 56.0, 43.2, 29.7, 22.6. ^{19}F NMR (376 MHz, $CDCl_3$) δ -114.90. HRMS (ESI) calculated for $C_{17}H_{23}FN_3O_2S^+[M+H]^+$: 352.1490, found: 352.1493.

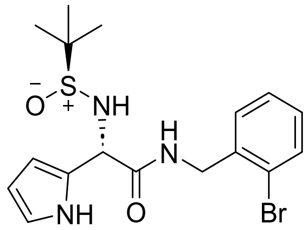 compound **33**

(*S*)-*N*-(2-bromobenzyl)-2-(((*S*)-*tert*-butylsulfinyl)amino)-2-(1*H*-pyrrol-2-yl)acetamide compound **33:** Prepared by using the representative procedure above from (*S,E*)-*N*-(2-bromobenzyl)-2-((*tert*-butylsulfinyl)imino)acetamide **2e** and compound **3a** (0.10g, 1.5mmol). Purified by column chromatography on silica gel (DCM:MeOH =20:1) to afford compound **33** (446 mg, 87% yield) as a white solid: $[\alpha]_D^{20}$= +120.5 (c 0.2, MeOH). m.p. 112.5-114.3 °C. IR (KBr) Vmax: 3733, 3636, 3558, 2981, 1705, 1663, 1608, 1567, 1530, 1513, 1452, 1410, 1362, 1037, 664 cm^{-1}. ^1H NMR (400 MHz, $CDCl_3$) δ 9.48 (s, 1H), 7.39 (d, J = 8.0 Hz, 2H), 7.02 (d, J = 8.0 Hz, 2H), 6.76 (s, 1H), 6.19 – 6.09 (m, 2H), 5.29 (s, 1H), 5.14 (d, J = 4.9 Hz, 1H), 4.82 (d, J = 5.0 Hz, 1H), 4.32 (q, J = 7.4, 5.6 Hz, 2H), 1.23 (s, 9H). ^{13}C NMR (100 MHz, $CDCl_3$) δ 170.4, 136.7, 131.8, 129.2, 127.1, 121.3, 119.0, 108.7, 107.8, 56.4, 55.9, 53.4, 31.9, 29.7, 22.6. HRMS (ESI) calculated for $C_{17}H_{23}BrN_3O_2S^+[M+H]^+$: 412.0689, found: 412.0691.

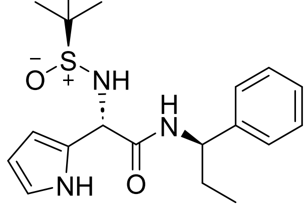 compound **34**

(*S*)-2-(((*S*)-*tert*-butylsulfinyl)amino)-*N*-((*R*)-1-phenylpropyl)-2-(1*H*-pyrrol-2-yl)acetamide compound **34:** Prepared by using the representative procedure above from (*E*)-2-(((*S*)-*tert*-butylsulfinyl)imino)-*N*-((*R*)-1-phenylpropyl)acetamide **2f** and compound **3a** (0.10g, 1.5mmol). Purified by column chromatography on silica gel (DCM:MeOH =20:1) to afford compound **34** (418

mg, 93% yield) as a white solid: $[\alpha]_D^{20}$= + 34.21 (c 0.15, MeOH). m.p. 121.5-123.1 °C. IR (KBr) Vmax: 3305, 3058, 2994, 2736, 1633, 1539, 1468, 1368, 1178, 1176, 1053, 1013, 996, 824, 694 cm^{-1}. ^1H NMR (400 MHz, $CDCl_3$) δ 9.43 (s, 1H), 7.32 － 7.27 (m, 2H), 7.22 (td, J = 7.1, 1.3 Hz,3H), 6.77 (q, J = 1.1 Hz, 1H), 6.18 (dt, J = 3.0, 1.7 Hz, 1H), 6.16 (q, J = 2.9 Hz, 1H), 5.11 (d, J = 5.5 Hz, 1H), 4.79 (q, J = 7.5 Hz, 1H), 4.57 (d, J = 5.6 Hz, 1H), 1.75 (p, J = 7.4 Hz, 2H), 1.27 (s, 9H), 0.80 (t, J = 7.4 Hz, 3H). ^{13}C NMR (100 MHz, $CDCl_3$) δ 169.6, 141.8, 128.7, 127.4, 127.2, 126.5, 119.0, 108.5, 107.7, 56.5, 56.2, 55.7, 29.3, 22.6, 10.6. HRMS (ESI) calculated for $C_{19}H_{28}N_3O_2S^+[M+H]^+$: 362.1897, found: 362.1899.

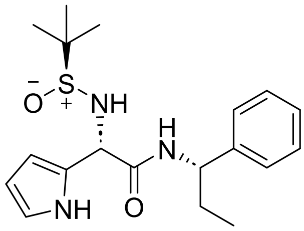

compound **35**

(*S*)-2-(((*S*)-*tert*-butylsulfinyl)amino)-*N*-((*S*)-1-phenylpropyl)-2-(1*H*-pyrrol-2-yl)acetamide

compound **35:** Prepared by using the representative procedure above from (*E*)-2-(((*S*)-*tert*-butylsulfinyl)imino)-*N*-((*S*)-1-phenylpropyl)acetamide **2g** and compound **3a** (0.10g, 1.5mmol). Purified by column chromatography on silica gel (DCM:MeOH =20:1) to afford compound **34** (416 mg, 93% yield) as a white solid: $[\alpha]_D^{20}$= + 35.42 (c 0.15, MeOH). m.p. 123.6-125.8 ℃. IR (KBr) Vmax: 3296, 3065, 2959, 2860, 1660, 1522, 1456, 1364, 1130, 1078, 1040, 885, 728, 698 cm^{-1}. ^1H NMR (400 MHz, $CDCl_3$) δ 9.24 (s, 1H), 7.28 (d, *J* = 7.3 Hz, 2H), 7.22 (s, 1H), 7.18 (d, *J* = 7.9 Hz, 1H), 7.14 (d, *J* = 7.5 Hz, 2H), 6.77 (s, 1H), 6.21 (s, 1H), 6.18 (s, 1H), 5.10 (d, *J* = 5.6 Hz, 1H), 4.74 (d, *J* = 7.5 Hz, 1H), 4.61 (d, *J* = 5.5 Hz, 1H), 1.78 – 1.73 (m, 2H), 1.28 (s, 9H), 0.87 (d, *J* = 7.4 Hz, 3H). ^{13}C NMR (100 MHz, $CDCl_3$) δ 169.6, 141.9, 128.5, 127.2, 127.1, 126.2, 119.0, 107.7, 56.4, 56.0, 55.8, 31.5, 29.5, 22.6, 22.6, 22.5, 14.0, 10.6. HRMS (ESI) calculated for $C_{19}H_{28}N_3O_2S^+[M+H]^+$: 362.1897, found: 362.1898.

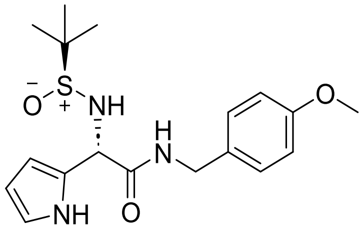

compound **36**

(*S*)-2-(((*S*)-*tert*-butylsulfinyl)amino)-*N*-(4-methoxybenzyl)-2-(1*H*-pyrrol-2-yl)acetamide

compound **36:** Prepared by using the representative procedure above from (*S,E*)-2-((*tert*-butylsulfinyl)imino)-*N*-(4-methoxybenzyl)acetamide **2h** and compound **3a** (0.10g, 1.5mmol). Purified by column chromatography on silica gel (DCM:MeOH =20:1) to afford compound **35** (435 mg, 95% yield) as a white solid: $[\alpha]_D^{20}$= + 48.96 (c 0.2, MeOH). m.p. 125.4-127.0 °C. IR (KBr) Vmax: 3305, 3287, 3058, 2944, 2908, 2836, 1633, 1567, 1530, 1464, 1440, 1368, 1241, 1170, 1121, 1013, 1055, 911, 808, 730 cm^{-1}. ^1H NMR (400 MHz, $CDCl_3$) δ 9.35 (s, 1H), 7.10 (d, J = 8.6 Hz, 2H), 7.05 (t, J = 5.6 Hz, 1H), 6.85 － 6.78 (m, 2H), 6.76 (q, J = 2.3 Hz, 1H), 6.16 (dd, J = 12.1, 2.6 Hz, 2H), 5.11 (d, J = 4.8 Hz, 1H), 4.76 (d, J = 4.8 Hz, 1H), 4.32 (qd, J = 14.7, 5.7 Hz, 2H), 3.77 (s, 3H), 1.24 (s, 9H). ^{13}C NMR (100 MHz, $CDCl_3$) δ 170.0, 159.0, 129.7, 128.9, 127.1, 119.0, 114.1, 108.6, 107.9, 60.4, 56.3, 55.8, 55.3, 43.4, 29.7, 22.6, 14.2. HRMS (ESI) calculated for

$C_{18}H_{26}N_3O_3S^+$[M+H]$^+$: 364.1689, found: 364.1693.

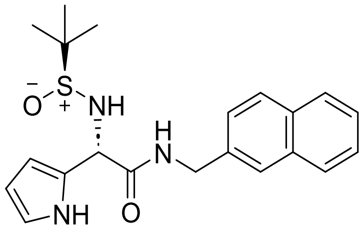

compound **37**

(*S*)-2-(((*S*)-*tert*-butylsulfinyl)amino)-*N*-(naphthalen-2-ylmethyl)-2-(1*H*-pyrrol-2-yl)acetamide compound **37:** Prepared by using the representative procedure above from (*S,E*)-2-((*tert*-butylsulfinyl)imino)-*N*-(naphthalen-2-ylmethyl)acetamide **2k** and compound **3a** (0.10g, 1.5mmol). Purified by column chromatography on silica gel (DCM:MeOH =20:1) to afford compound **36** (445 mg, 92% yield) as a white solid: $[\alpha]_D^{20}$= + 65.1 (c 0.2, MeOH). m.p. 138.1-139.3 °C. IR (KBr) Vmax: 3678, 3576, 2962, 2890,1843, 1735, 1687, 1651, 1560, 1506, 1464, 1368, 1061, 670 cm^{-1}. ^{1}H NMR (400 MHz, $CDCl_3$) δ 9.47 (s, 1H), 7.80 – 7.68 (m, 3H), 7.51 (d, J = 1.8 Hz, 1H), 7.43 (tt, J = 5.6, 4.6 Hz, 2H), 7.29 (t, J = 5.8 Hz, 1H), 7.25 – 7.23 (m, 1H), 6.76 (td, J = 2.6, 1.5 Hz, 1H), 6.20 (dt, J = 4.0, 1.9 Hz, 1H), 6.17 (q, J = 2.9 Hz, 1H), 5.18 (d, J = 4.7 Hz, 1H), 4.88 (d, J = 4.7 Hz, 1H), 4.59 – 4.42 (m, 2H), 1.18 (s, 9H). ^{13}C NMR (100 MHz, $CDCl_3$) δ 170.3, 135.0, 133.3, 132.7, 128.5, 127.8, 127.6, 127.3, 126.2, 126.0, 125.9, 125.6, 119.0, 108.7, 107.9, 56.3, 55.8, 43.9, 32.0, 29.7, 22.6. HRMS (ESI) calculated for $C_{21}H_{26}N_3O_2S^+$[M+H]$^+$: 384.1740, found: 384.1742.

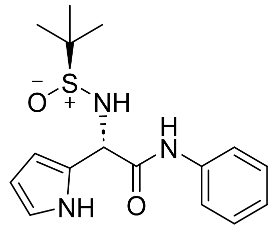

compound **38**

(*S*)-2-(((*S*)-*tert*-butylsulfinyl)amino)-*N*-phenyl-2-(1*H*-pyrrol-2-yl)acetamide compound **38:** Prepared by using the representative procedure above from (*S,E*)-2-((*tert*-butylsulfinyl)imino)-*N*-phenylacetamide **2i** and compound **3a** (0.10g, 1.5mmol). Purified by column chromatography on silica gel (DCM:MeOH =20:1) to afford compound **37** (369 mg, 95% yield) as a white solid: $[\alpha]_D^{20}$= + 78.76 (c 0.2, MeOH). m.p. 121-122 °C. IR (KBr) Vmax: 3255, 3058, 2892, 1645, 1456, 1378, 1319, 1254, 1158, 1137, 924, 808, 688, 579 cm^{-1}. ^{1}H NMR (400 MHz, $CDCl_3$) δ 9.39 (s, 1H), 8.81 (s, 1H), 7.45 (d, *J* = 8.1 Hz, 2H), 7.32 – 7.20 (m, 2H), 7.08 (t, *J* = 7.2 Hz, 1H), 6.80 (s, 1H), 6.26 (s, 1H), 6.19 (d, *J* = 2.8 Hz, 1H), 5.26 (s, 1H), 4.80 (s, 1H), 1.31 (s, 9H). ^{13}C NMR (100 MHz, $CDCl_3$) δ 168.4, 137.3, 128.9, 126.8, 124.8, 120.0, 119.3, 108.7, 108.1, 56.7, 56.6, 22.7. HRMS (ESI) calculated for $C_{16}H_{22}N_3O_2S^+$[M+H]$^+$: 320.1427, found: 320.1430.

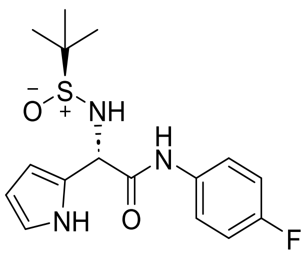

compound **39**

(*S*)-2-(((*S*)-*tert*-butylsulfinyl)amino)-*N*-(4-fluorophenyl)-2-(1*H*-pyrrol-2-yl)acetamide compound **39:** Prepared by using the representative procedure above from (*S,E*)-2-((*tert*-butylsulfinyl)imino)-

N-(4-fluorophenyl)acetamide **2j** and compound **3a** (0.10g, 1.5 mmol). Purified by column chromatography on silica gel (DCM:MeOH =20:1) to afford compound **38** (398 mg, 95% yield) as a white solid: $[\alpha]_D^{20}$= + 42.5 (c 0.2, MeOH). m.p. 115.6-117.4 °C. IR (KBr) Vmax: 3245, 3058, 2842, 1645, 1446, 1368, 1319, 1224, 1157, 1037, 929, 808, 712, 685, 529 cm^{-1}. ^1H NMR (400 MHz, $CDCl_3$) δ 9.47 (s, 1H), 9.17 (s, 1H), 7.37 (dd, *J* = 9.0, 4.7 Hz, 2H), 6.89 (t, *J* = 8.7 Hz, 2H), 6.76 (dd, *J* = 2.9, 1.7 Hz, 1H), 6.23 (q, *J* = 2.4, 1.8 Hz, 1H), 6.17 (d, *J* = 3.0 Hz, 1H), 5.27 (d, *J* = 5.8 Hz, 1H), 5.00 (d, *J* = 6.1 Hz, 1H), 1.32 (s, 9H). ^{13}C NMR (100 MHz, $CDCl_3$) δ 168.4, 160.7, 158.3, 133.5, 126.9, 121.7, 119.1, 115.6, 115.3, 108.7, 107.8, 56.8, 56.6, 29.7, 22.7. ^{19}F NMR (376 MHz, $CDCl_3$) δ -117.61. HRMS (ESI) calculated for $C_{16}H_{21}FN_3O_2S^+[M+H]^+$: 338.1333, found: 338.1335.

4. The synthesis of derivative 40, determination of *ee* value by HPLC, and single crystal data

4.1 The synthesis of derivative **40**

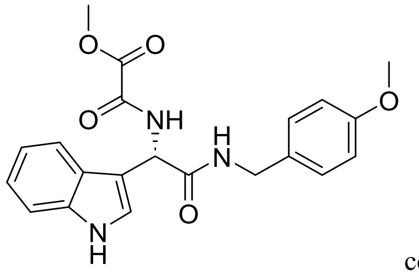

compound **40**

Methyl (*S*)-2-((1-(1*H*-indol-3-yl)-2-((4-methoxybenzyl)amino)-2-oxoethyl)amino)-2-oxoacetate compound **40:** In a round bottom flask, a solution of compound **24** (0.10 g, 0.24 mmol) in anhydrous methanol (1.2 mL) mixed with HCl (0.3 mL, 4M in MeOH)in a nitrogen atmosphere at room temeperature for 0.5 hours. After the substrate was completely consumed (monitored by TLC analysis), after removed the solvent, the reaction mixture was quenched with 10% of NaOH (20 mL).The mixture was extracted by ethyl acetate (3 × 20 mL).The combined organic phase was dried over Na_2SO_4, and concentrated in *vacuo*. The residue was purified by flash column chromatography on silica gel (dichloromethane: methanol=20:1) to furnish the desired compound **40** (67.9 mg, 71% yield) as a light pink solid**:** $[\alpha]_D^{20}$= + 58.8 (c 0. 1, MeOH). m.p. 182.4-184.1 °C. IR (KBr) Vmax: 3395, 3333, 3210, 3063, 3001, 2955, 2839, 2338, 1751, 1690, 1651, 1520, 1458, 1435, 1335, 1288, 1250, 1227, 1173, 1103, 1034, 980, 926, 826, 741, 640, 571, 532, 424cm^{-1}. ^1H NMR (400 MHz, DMSO-d_6) δ 11.13 (d, *J* = 2.6 Hz, 1H), 8.89 (d, *J* = 8.0 Hz, 1H), 8.69 (t, *J* = 5.8 Hz, 1H), 7.61 (d, *J* = 7.9 Hz, 1H), 7.39 (d, *J* = 8.1 Hz, 1H), 7.29 (d, *J* = 2.6 Hz, 1H), 7.16 – 7.09 (m, 3H), 7.01 (td, *J* = 7.4, 7.0, 1.0 Hz, 1H), 6.85 – 6.81 (m, 2H), 5.76 (d, *J* = 8.0 Hz, 1H), 4.31 – 4.19 (m, 2H), 3.76 (s, 3H), 3.72 (s, 3H). ^{13}C NMR (100 MHz, DMSO-d_6) δ 169.6, 161.7, 158.7, 157.2, 136.6, 131.4, 129.1, 126.1, 125.0, 121.9, 119.4, 119.4, 114.1, 111.3, 55.5, 53.3, 50.3, 42.2. HRMS (ESI) calculated for $C_{21}H_{22}N_3O_5^+[M+H]^+$: 396.1554, found: 396.1556.

4.2 HPLC analysis for determination of the *ee* value of compound **40**

The crude racemic-**40** and compound **40** were analyzed directly by HPLC (Essentia LC-16) using Chiral AD-H column at 220 nm. Eluted by Hexane/EtOH/DEA (40:60:0.1 V:V:V).

(1) Racemic-**40**

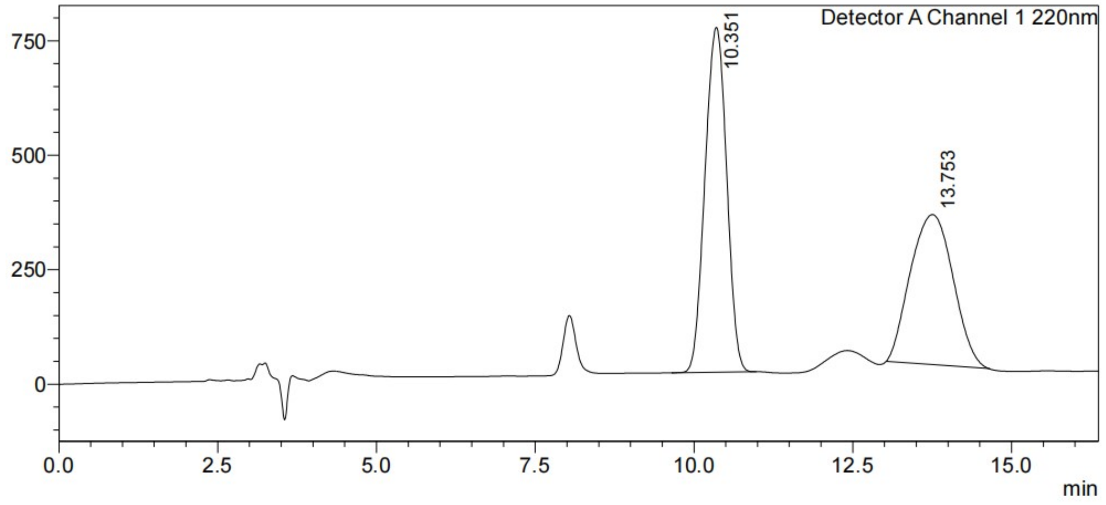

Peak#	Ret. Time	Area	Height	Conc.	Unit	Mark	Name
1	10.351	17885306	752888	53.831		M	
2	13.753	15339475	327751	46.169		M	
Total		33224781	1080639				

(2) Compound **40**

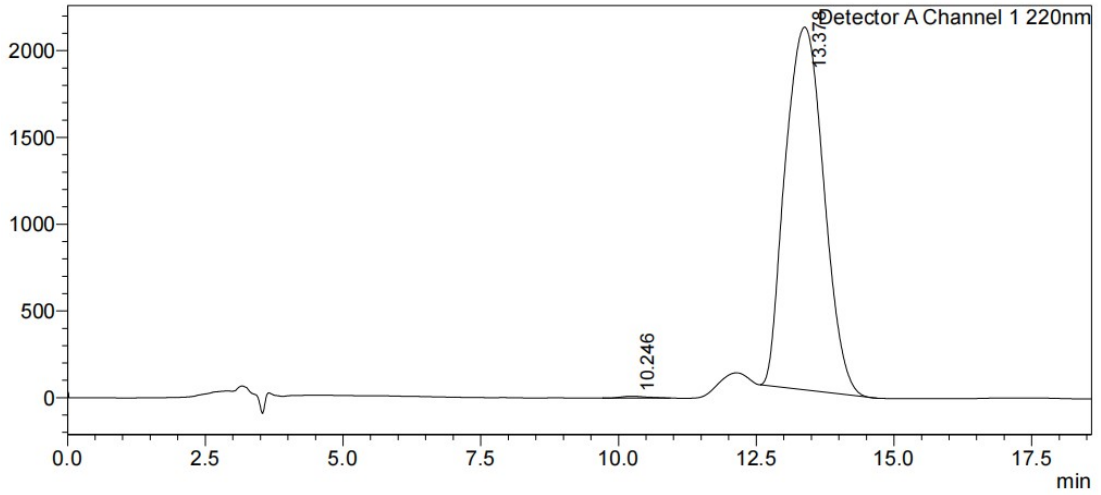

Peak#	Ret. Time	Area	Height	Conc.	Unit	Mark	Name
1	10.246	355549	11003	0.347		M	
2	13.378	102071629	2090303	99.653		M	
Total		102427178	2101306				

4.3 Crystal data of compound **40**

(1) Single crystal growth

A solution of compound **40** (10 mg) in methanol (MeOH, 1 mL) was prepared in a 10 mL vial. After complete dissolution, n-hexane (3.0 mL) was carefully layered along the vial wall, forming a biphasic system (MeOH/n-hexane, v/v = 1:3). The vial was sealed and left undisturbed at room temperature for slow solvent evaporation. After 7-10 days, well-formed colorless crystals suitable for X-ray diffraction analysis were obtained.

(2) X-ray crystallography

Single-crystal X-ray diffraction data were collected on a Rigaku XtaLAB Pro II AFC12

diffractometer (Cu Kα radiation, λ = 1.54184 Å) equipped with a HyPix-3000 detector. The X-ray generator was operated at 50 kV and 1.0 mA. Single crystals of $C_{21}H_{21}N_3O_5$ (1-3_auto) were obtained. A suitable crystal was selected and mounted on a XtaLAB Pro II AFC12 (RINC): Kappa single diffractometer. The crystal was kept at 293K during data collection. The ellipsoid probability level was set at 50%. Using Olex2, the structure was solved with the SHELXT structure solution program using Intrinsic Phasing and refined with the SHELXL refinement package using Least Squares minimisation. CCDC: 2322830 contains the supplementary crystallographic data for this work.

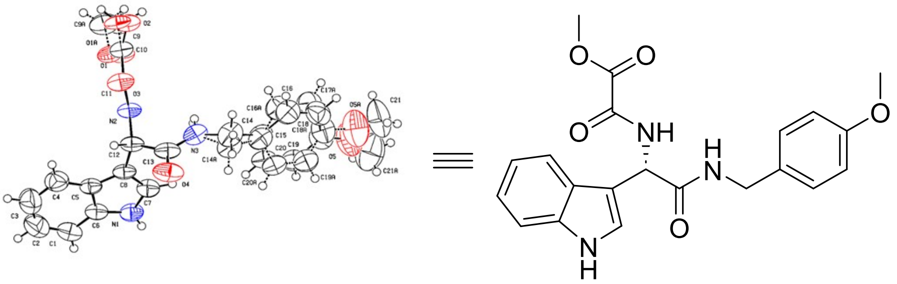

X-ray of 40 **40**

Crystal data and structure refinement for compound **40**

CCDC code	2322830
Identification code	1-3_auto
Empirical formula	$C_{21}H_{21}N_3O_5$
Formula weight	395.41
Temperature/K	293.00
Crystal system	trigonal
Space group	$P3_121$
a/Å	9.5132(2)
b/Å	9.5132(2)
c/Å	39.0933(15)
α/°	90
β/°	90
γ/°	120
Volume/Å^3	3063.98(17)
Z	6
ρ_{calc}g/cm^3	1.286
μ/mm^{-1}	0.772
F(000)	1248.0
Crystal size/mm^3	0.06 × 0.05 × 0.04
Radiation	Cu Kα (λ = 1.54184)
2Θ range for data collection/°	6.784 to 136.606
Index ranges	$-11 \le h \le 11$, $-9 \le k \le 11$, $-47 \le l \le 46$
Reflections collected	22638
Independent reflections	3728 [R_{int} = 0.0559, R_{sigma} = 0.0416]
Data/restraints/parameters	3728/385/353

Goodness-of-fit on F^2	1.068
Final R indexes [I>=2σ (I)]	R_1 = 0.0504, wR_2 = 0.1273
Final R indexes [all data]	R_1 = 0.0671, wR_2 = 0.1456
Largest diff. peak/hole / e Å^{-3}	0.21/-0.17
Flack parameter	0.10(19)

5. Total synthesis of the bisindole alkaloid Calcicamide B

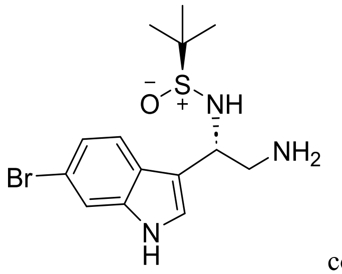

compound **41**

(*S*)-*N*-((*S*)-2-amino-1-(6-bromo-1*H*-indol-3-yl)ethyl)-2-methylpropane-2-sulfinamide compound **41:** In a temperature below 0 °C, a solution of compound **11** (6.0 g, 13.0 mmol, 1.0 eq.) in freshly distilled THF (26 mL) was added dropwise to a solution of lithium aluminum hydride (24.2 mL, 2.5 mol/L, 4.6 eq.) in freshly distilled THF (91 mL) and the mixture was boiled under reflux for 6 h. The solution was cooled. Then 10 mL of water was added dropwise. Filter, evaporate the filtrate to remove the solvent, and distill the residue under reduced pressure to obtain the intermediate, which was directly used for the next step. The intermediate obtained above was dissolved in 29 mL of ethanol and 29 mL of THF, and 0.28 g (0.26 mmol) of palladium (10 % on carbon) were added. The mixture was stirred under a hydrogen atmosphere at atmospheric pressure for 2h at room temperature. The reaction mixture was then filtered off with suction through kieselguhr, the residue was washed with THF and the filtrate was concentrated. The crude product was purified by chromatography on silica gel (dichloromethane: methanol=10:1) to obtain the compound **41** (3.5 g, yield: 76%) as a yellow solid: $[\alpha]_D^{20}$= + 17.0 (c 0.1, MeOH). m.p. 95.5-98.6 °C. IR (KBr) Vmax: 3202, 2932, 1450, 1103, 1026, 802 cm^{-1}. ^{1}H NMR (400 MHz, DMSO-d_6) δ 11.21 – 11.10 (m, 1H), 7.54 (dd, *J* = 5.2, 3.3 Hz, 2H), 7.28 (d, *J* = 2.3 Hz, 1H), 7.09 (dd, *J* = 8.6, 1.8 Hz, 1H), 5.26 (d, *J* = 4.7 Hz, 1H), 4.38 (dt, *J* = 9.6, 5.0 Hz, 1H), 3.02 – 2.84 (m, 2H), 1.84 (s, 1H), 1.23 (d, *J* = 4.3 Hz, 1H), 1.08 (s, 9H). ^{13}C NMR (100 MHz, DMSO-d_6) δ 137.9, 125.4, 125.0, 121.7, 121.5, 115.3, 114.6, 114.3, 55.8, 55.2, 47.8, 23.1. HRMS (ESI) calculated for $C_{14}H_{21}BrN_3OS^+$[M+H]$^+$: 358.0583, found: 358.0585.

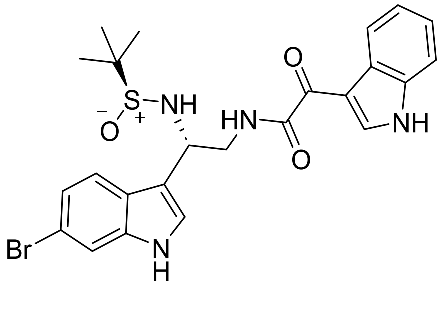

compound **42**

N-((*S*)-2-(6-bromo-1*H*-indol-3-yl)-2-(((*S*)-*tert*-butylsulfinyl)amino)ethyl)-2-(1*H*-indol-3-yl)-2-oxoacetamide compound **42**: Oxalyl chloride (2.79 mL, 2 mol/L, 1.5 eq.) was added dropwise into

a round bottomed flask containing newly distilled THF (9.25 mL) mixed with indole (0.435 g, 3.72 mmol, 1.0 eq.). After stirring in an argon environment for 1 hour at a temperature below 0 °C, the mixture was heated to room temperature and stirred for 1 hour to obtain the intermediate of the acyl chloride reaction. Triethylamine (1.15 mL, 0.84 mmol, 1.14 eq.) was added dropwise to a newly distilled THF (9.25 mL) solution mixed with compound **41** (1.0 g, 2.79 mmol, 0.75 eq.), and then the obtained acyl chloride intermediate was added dropwise to the mixture. Stirred at 0 °C for 2 hours, then saturated salt water (30 mL) was added to quench the reaction and extract with ethyl acetate (3 × 30 mL). The obtained organic phase was merged, dried with anhydrous sodium sulfate, and concentrated under vacuum concentration. The residue was purified by flash column chromatography (dichloromethane: methanol=20:1) to obtain compound **42**（1.29 g, yield: 88%）, as a white solid: $[\alpha]_D^{20}$= +4.8 (c 0.1, MeOH). m.p. 167.3-169.6 ℃. IR (KBr) Vmax: 3271, 2931, 1674, 1620, 1504, 1427, 1234, 1226, 1026, 897, 794, 756, 663 cm^{-1}. ^{1}H NMR (400 MHz, DMSO-d_6) δ 12.23 (s, 1H), 11.12 (d, *J* = 2.4 Hz, 1H), 8.90 (t, *J* = 6.1 Hz, 1H), 8.71 (s, 1H), 8.24 – 8.19 (m, 1H), 7.62 (d, *J* = 8.5 Hz, 1H), 7.55 (d, *J* = 1.8 Hz, 1H), 7.54 – 7.52 (m, 1H), 7.36 (d, *J* = 2.5 Hz, 1H), 7.30 – 7.22 (m, 2H), 7.13 (dd, *J* = 8.5, 1.8 Hz, 1H), 5.39 (d, *J* = 5.0 Hz, 1H), 4.77 (dt, *J* = 7.4, 5.4 Hz, 1H), 3.80 – 3.65 (m, 2H), 1.10 (s, 9H). ^{13}C NMR (100MHz, DMSO-d_6) δ 181.7, 163.9, 138.5, 137.4, 136.2, 126.1, 124.9, 124.6, 123.4, 122.5, 121.4, 121.2, 120.9, 114.3, 114.1, 113.9, 112.5, 112.1, 54.9, 52.6, 22.6. HRMS (ESI) calculated for $C_{24}H_{26}BrN_4O_3S^+$[M+H]$^+$: 529.0904, found: 529.0908.

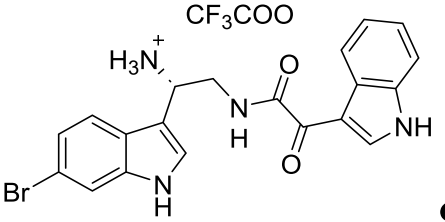

Calcicamide B

Calcicamide B: Compound **42** (1.0 g, 1.89 mmol, 1.0 eq.) was added to 7.6 mL of dichloromethane under an argon conditions. Trifluoroacetic acid (0.3 mL, 3.78 mmol, 2.0 eq.) was slowly added dropwise and stirred at room temperature for 1 hour. Washed with saturated salt water and extracted with dichloromethane (3 × 10 mL). The organic phase obtained was dried with anhydrous sodium sulfate, and the organic solvent was removed by vacuum concentration. The residue was concentrated and purified by flash column chromatography (dichloromethane: methanol=10:1) to obtain the product **Calcicamide B** (0.74 g, yield: 92%) as a yellow solid：$[\alpha]_D^{20}$= +3.6 (c 0.1, MeCN). m.p. 213.5-215.1 ℃. IR (KBr) Vmax: 3425, 3294, 3186, 1581, 1419, 1041, 1018, 925, 810, 648, 532 cm^{-1}. ^{1}H NMR (400 MHz, DMSO-d_6) δ 12.28 (d, *J* = 3.3 Hz, 1H), 11.47 (d, *J* = 2.6 Hz, 1H), 8.95 (t, *J* = 6.1 Hz, 1H), 8.77 (d, *J* = 3.2 Hz, 1H), 8.36 (s, 3H), 8.31 (s, 0H), 8.25 – 8.20 (m, 1H), 7.71 (d, *J* = 8.6 Hz, 1H), 7.64 (d, *J* = 1.8 Hz, 1H), 7.59 (d, *J* = 2.6 Hz, 1H), 7.56 – 7.53 (m, 1H), 7.28 – 7.25 (m, 2H), 7.23 (dd, *J* = 8.6, 1.8 Hz, 1H), 4.84 (s, 1H), 3.88 – 3.64 (m, 2H). ^{13}C NMR (100 MHz, DMSO-d_6) δ 180.9, 163.6, 138.6, 136.8, 136.2, 126.3, 125.2, 124.7, 123.5, 122.6, 122.1, 121.3, 120.3, 114.5, 114.4, 112.6, 112.0, 109.6, 46.8, 42.1. HRMS (ESI) calculated for $C_{20}H_{18}BrN_4O_2^+$[M+H]$^+$: 425.0608, found: 425.0612.

6. NMR spectra of compounds

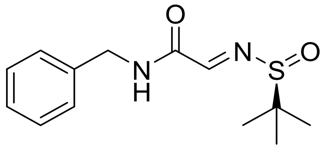

compound **2a**

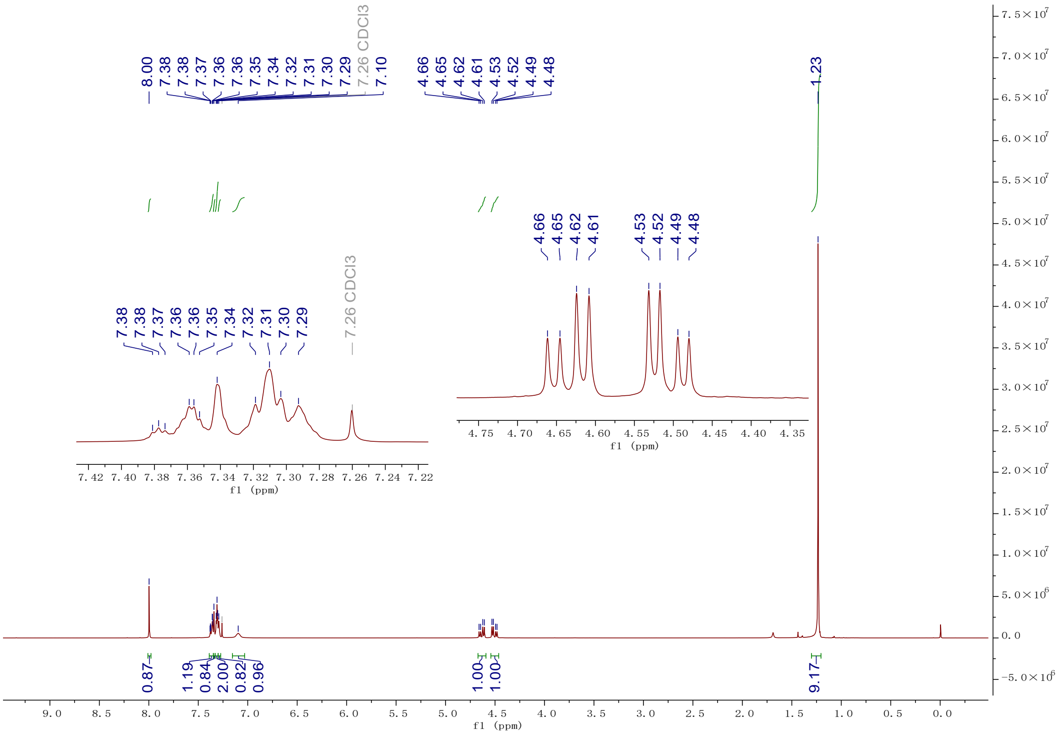

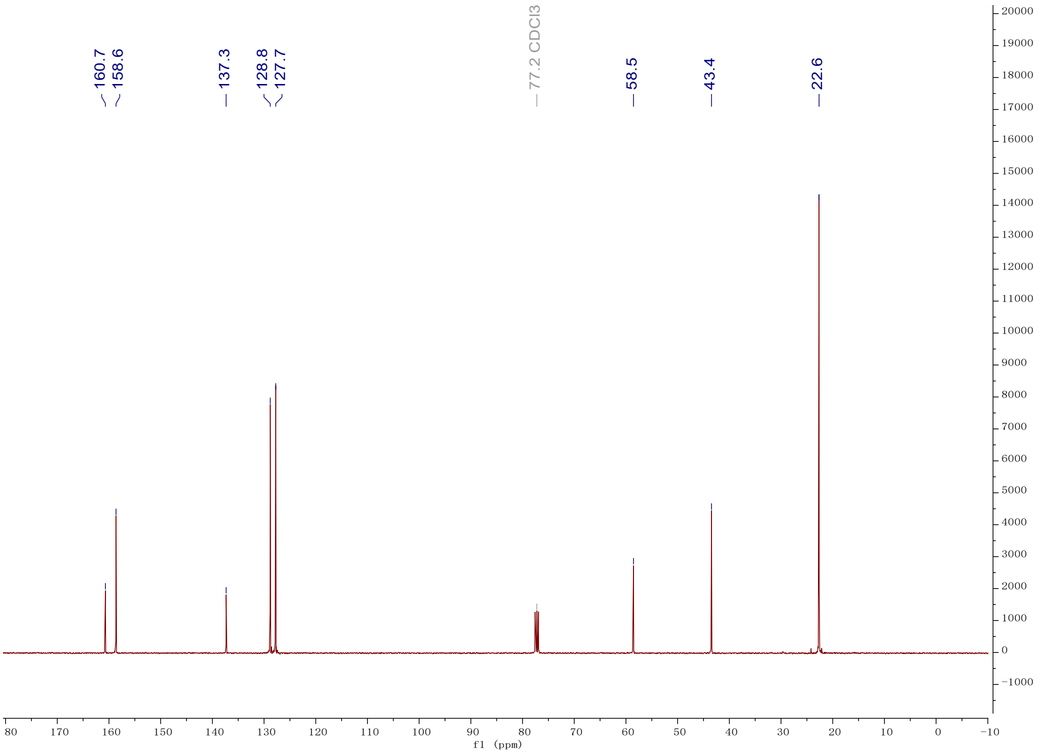

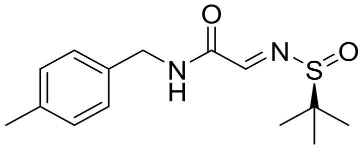
O
N
H
N
S
O

compound **2b**

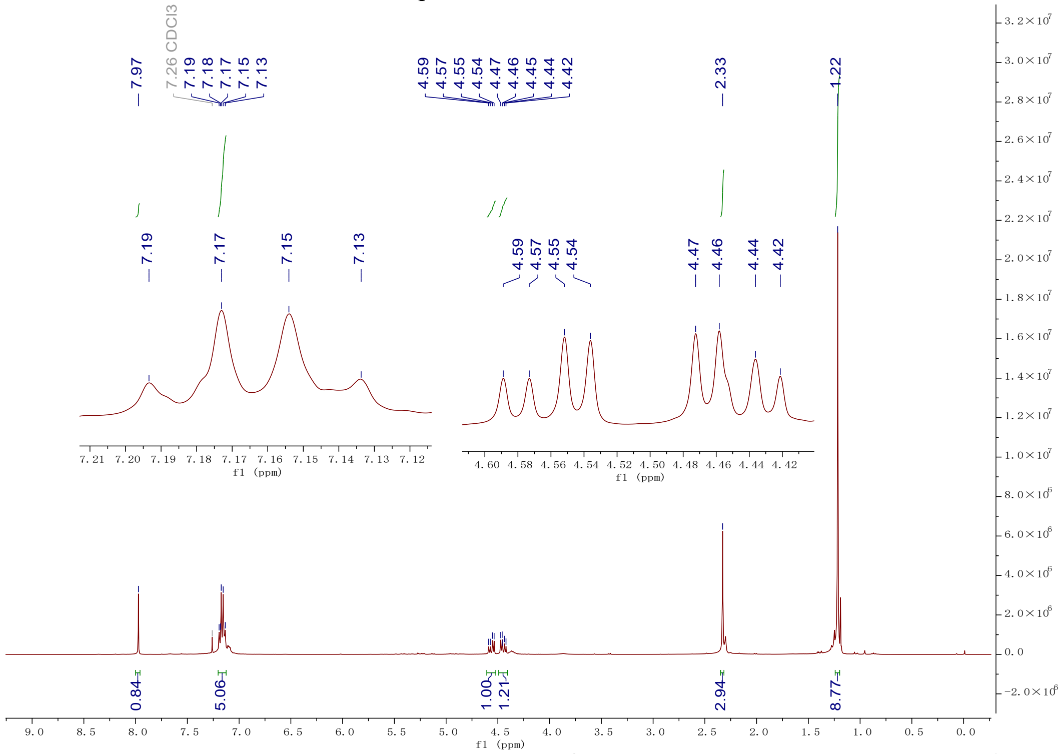
7.97
7.26 CDCl3
7.19
7.18
7.17
7.15
7.13
4.59
4.57
4.55
4.54
4.47
4.46
4.45
4.44
4.42
2.33
1.22
0.84
5.06
1.00
1.21
2.94
8.77
f1 (ppm)

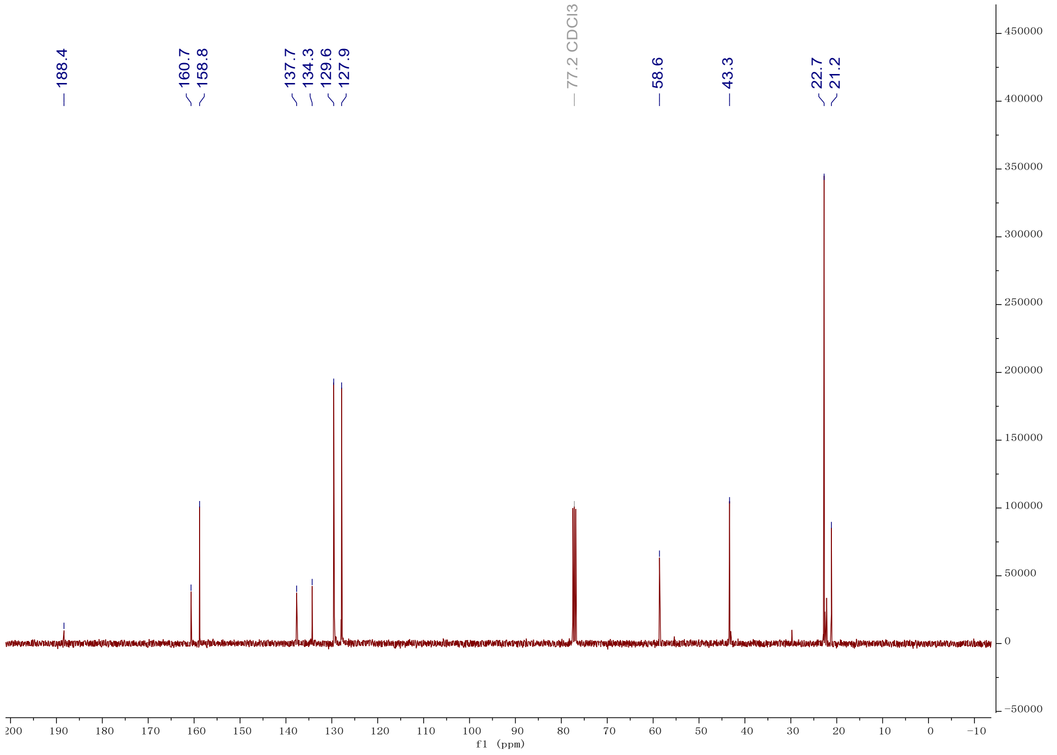
188.4
160.7
158.8
137.7
134.3
129.6
127.9
77.2 CDCl3
58.6
43.3
22.7
21.2
f1 (ppm)

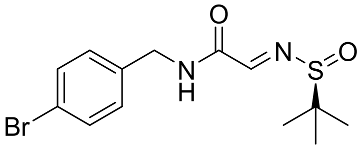
O
N
S
O
N
H
Br

compound **2c**

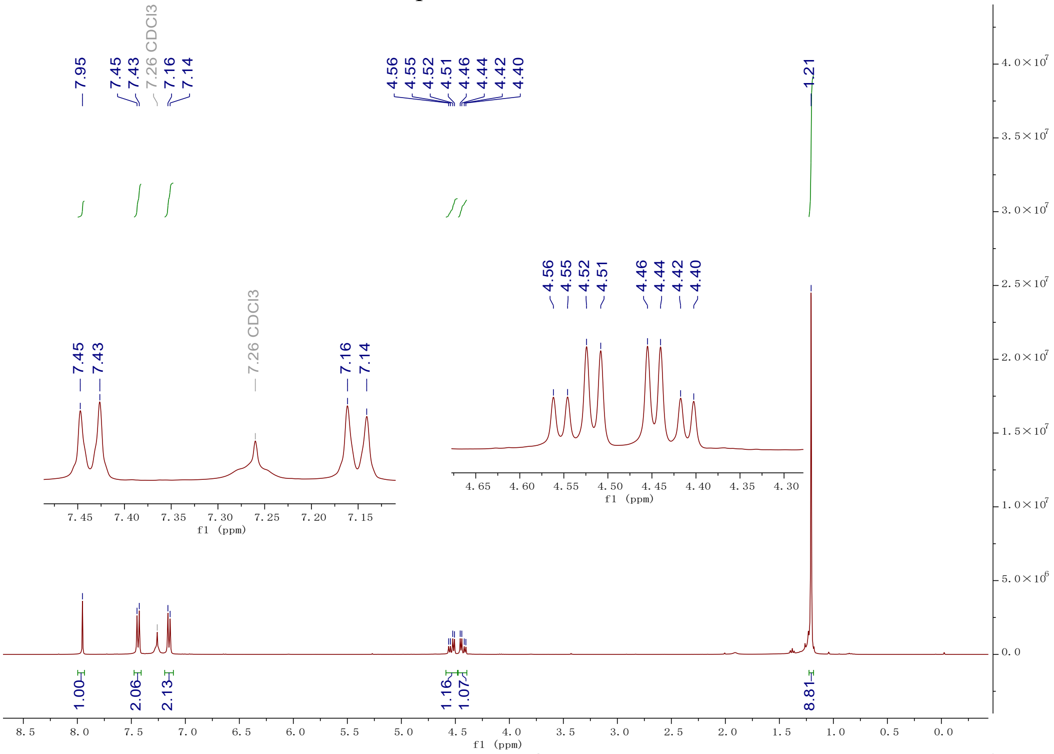
7.95
7.45
7.43
7.26 CDCl3
7.16
7.14
4.56
4.55
4.52
4.51
4.46
4.44
4.42
4.40
1.21
1.00
2.06
2.13
1.16
1.07
8.81
f1 (ppm)

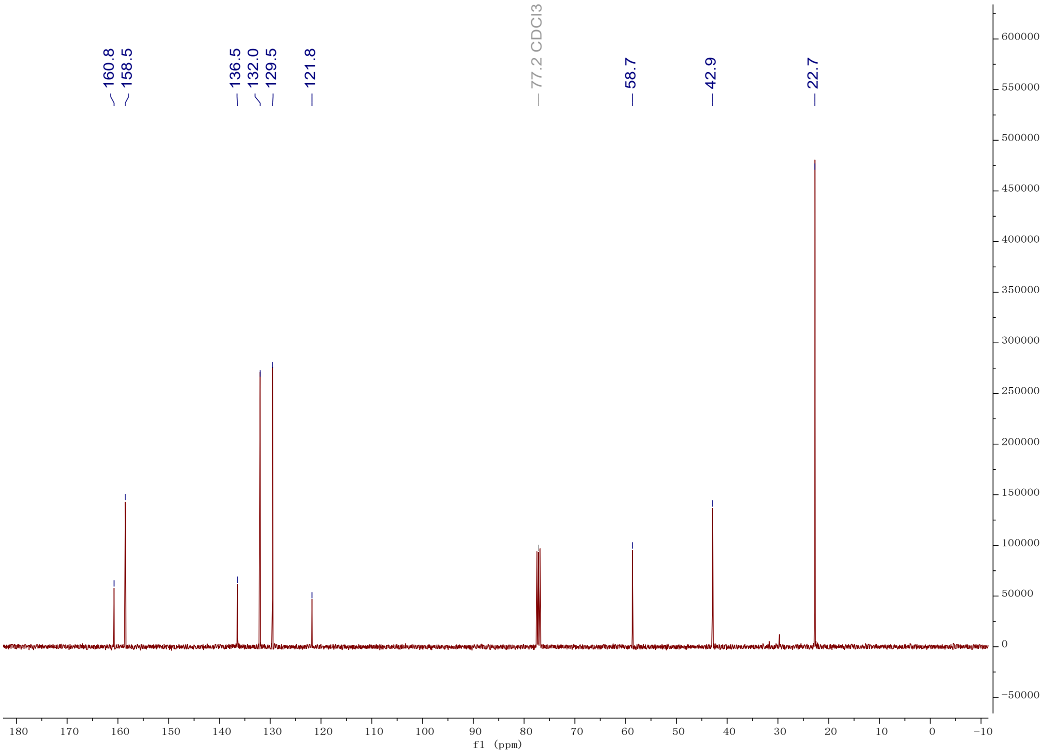
160.8
158.5
136.5
132.0
129.5
121.8
77.2 CDCl3
58.7
42.9
22.7
f1 (ppm)

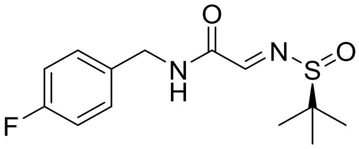

O
N
H
N
S
O
F

compound 2d

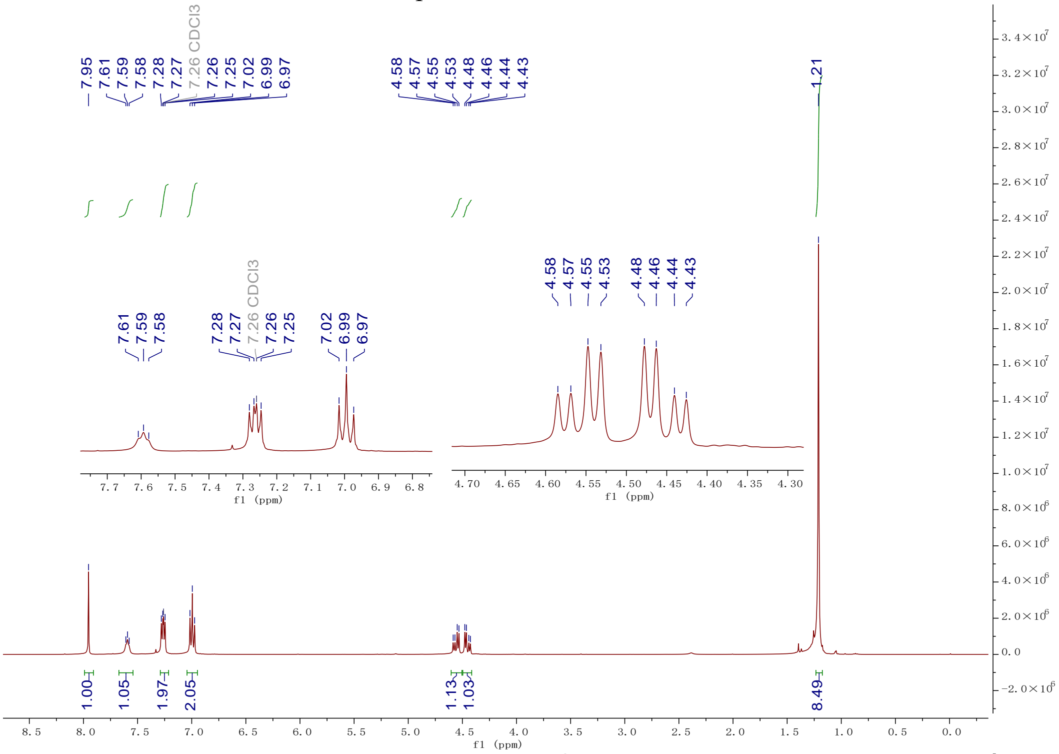

7.95
7.61
7.59
7.58
7.28
7.27
7.26 CDCl3
7.26
7.25
7.02
6.99
6.97
4.58
4.57
4.55
4.53
4.48
4.46
4.44
4.43
1.21
1.00
1.05
1.97
2.05
1.13
1.03
8.49
f1 (ppm)

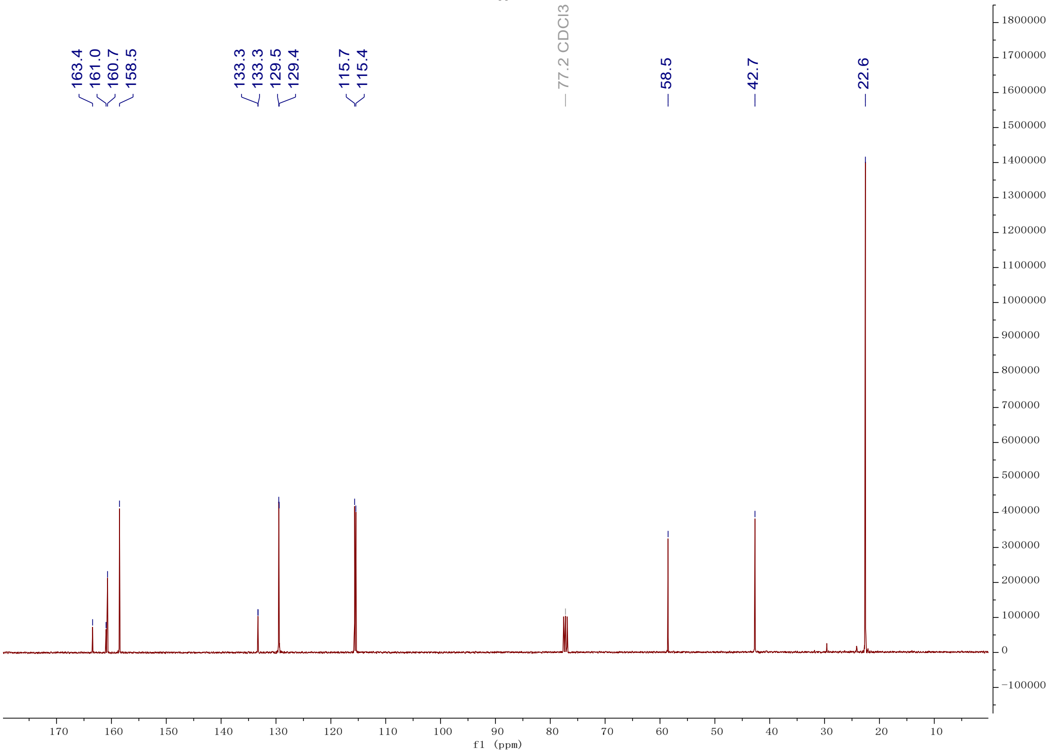

163.4
161.0
160.7
158.5
133.3
133.3
129.5
129.4
115.7
115.4
77.2 CDCl3
58.5
42.7
22.6
f1 (ppm)

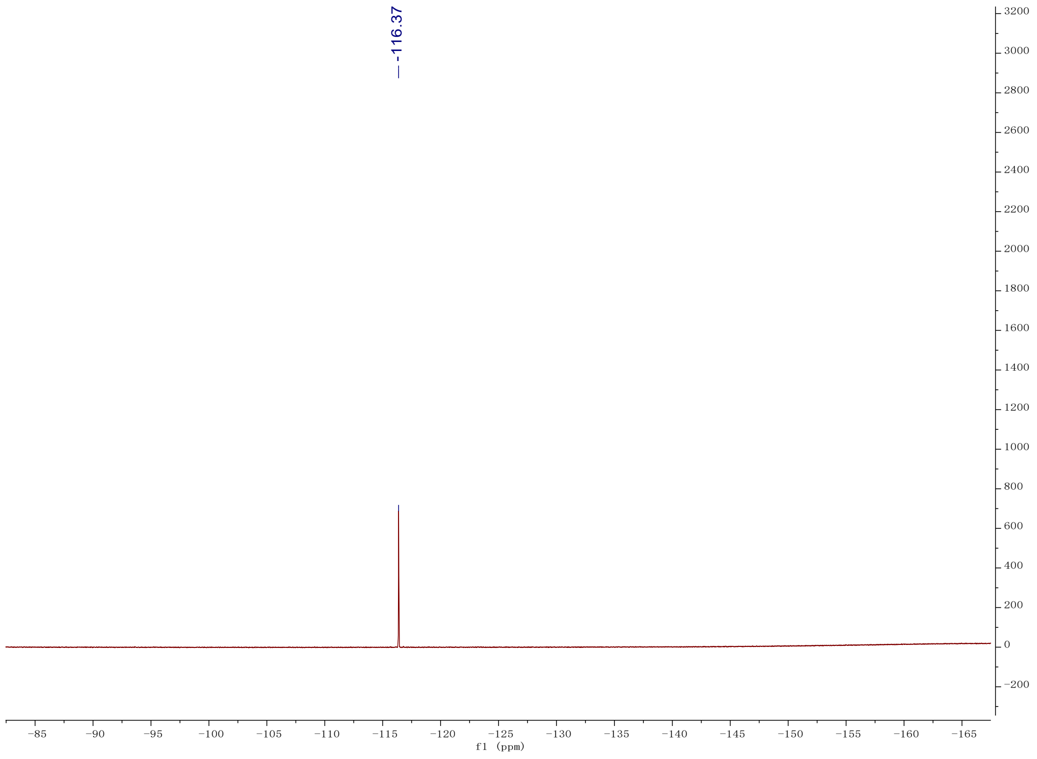
-116.37
f1 (ppm)

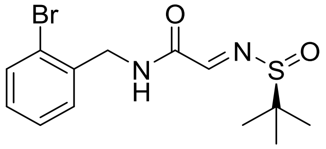
Br
O
N
H
N
S
O

compound **2e**

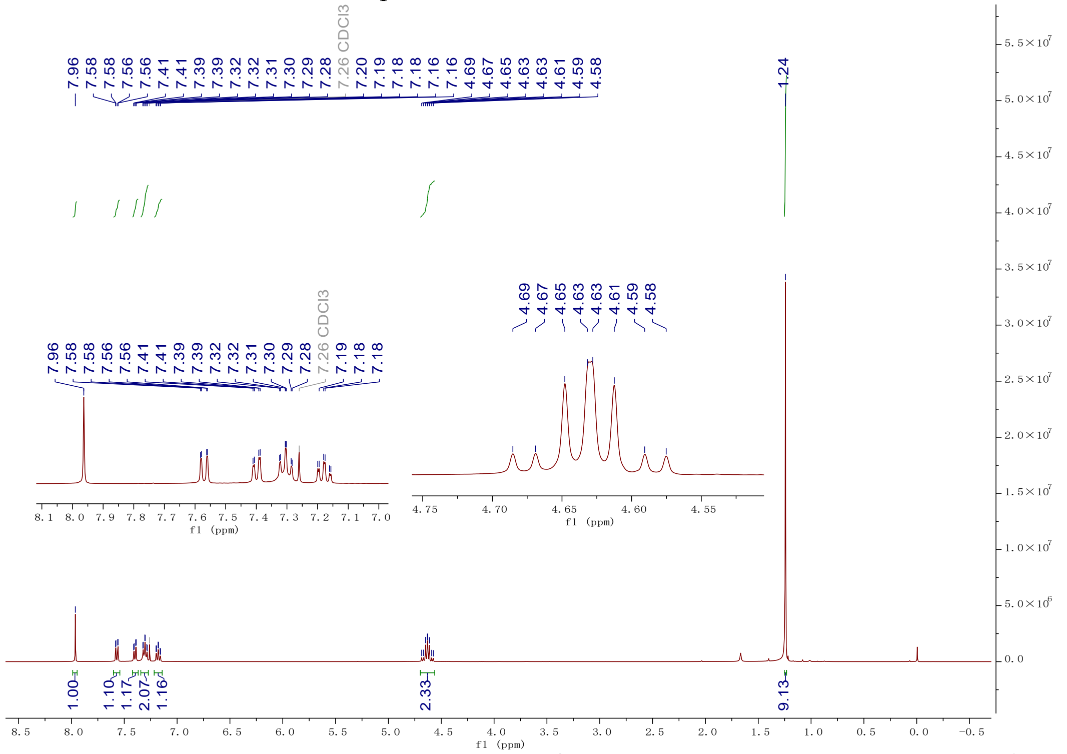
7.96
7.58
7.58
7.56
7.56
7.41
7.41
7.39
7.39
7.32
7.32
7.31
7.30
7.29
7.28
7.26 CDCl3
7.20
7.19
7.18
7.18
7.16
7.16
4.69
4.67
4.65
4.63
4.63
4.61
4.59
4.58
1.24
1.00
1.10
1.17
2.07
1.16
2.33
9.13
f1 (ppm)

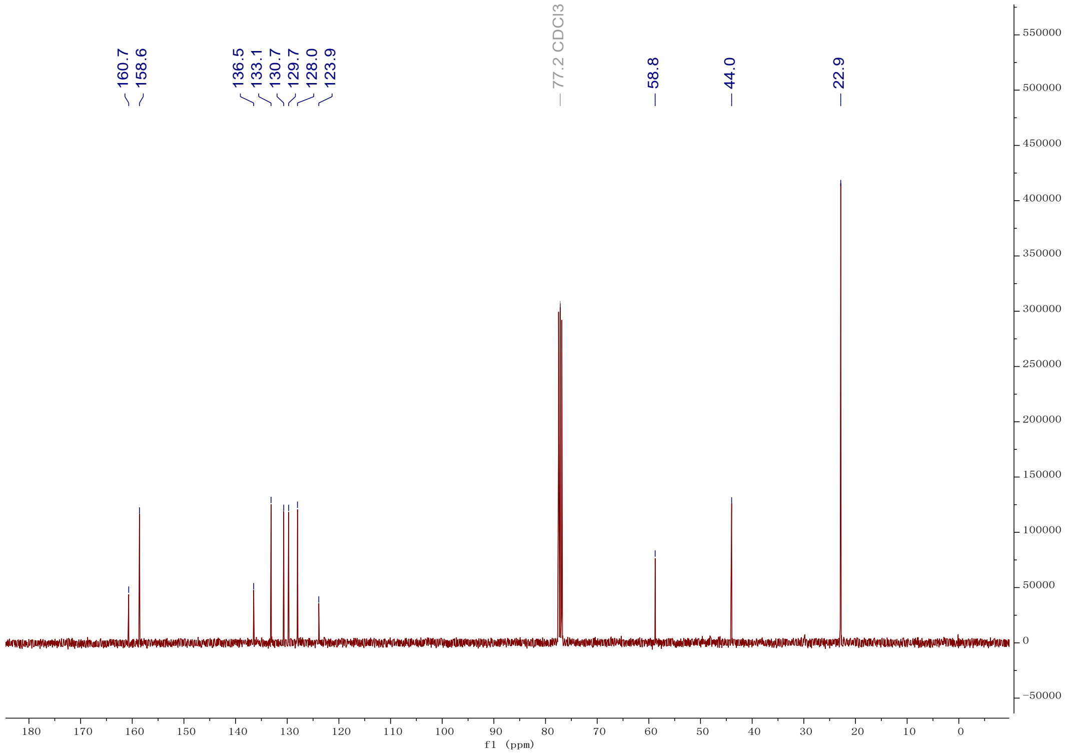
160.7
158.6
136.5
133.1
130.7
129.7
128.0
123.9
77.2 CDCl3
58.8
44.0
22.9
f1 (ppm)

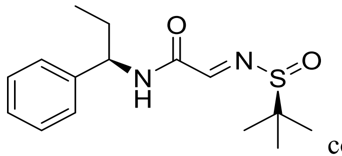

compound **2f**

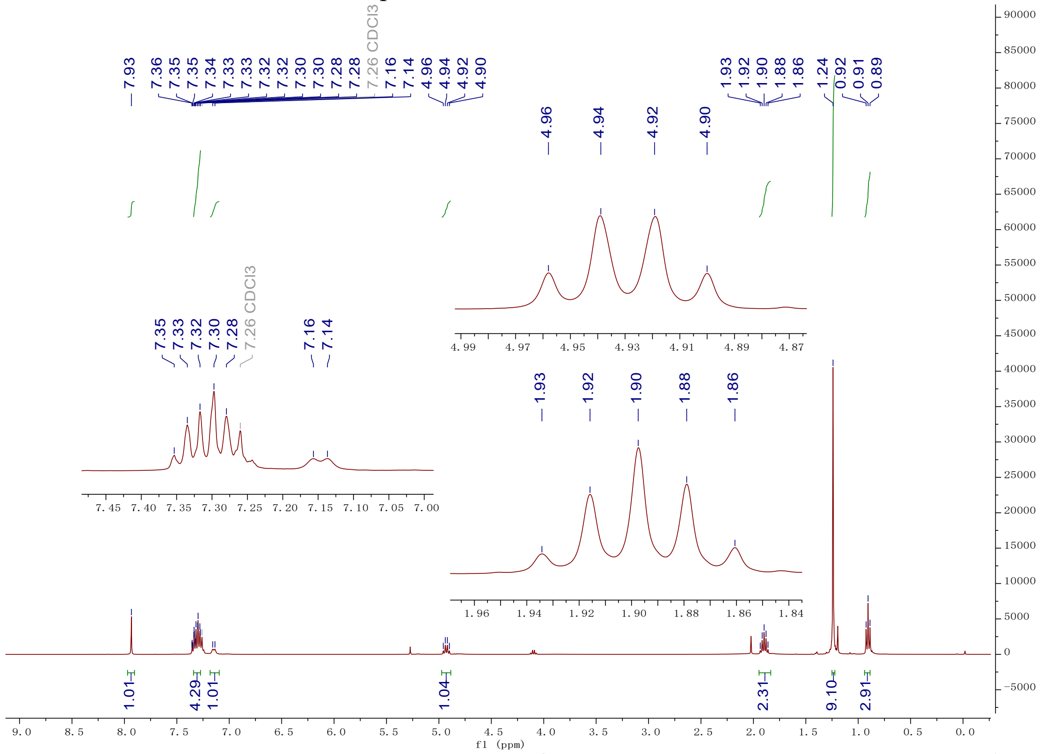

7.93
7.36
7.35
7.35
7.34
7.33
7.33
7.32
7.32
7.30
7.30
7.28
7.28
7.26 CDCl3
7.16
7.14
4.96
4.94
4.92
4.90
1.93
1.92
1.90
1.88
1.86
1.24
0.92
0.91
0.89
1.01
4.29
1.01
1.04
2.31
9.10
2.91
f1 (ppm)

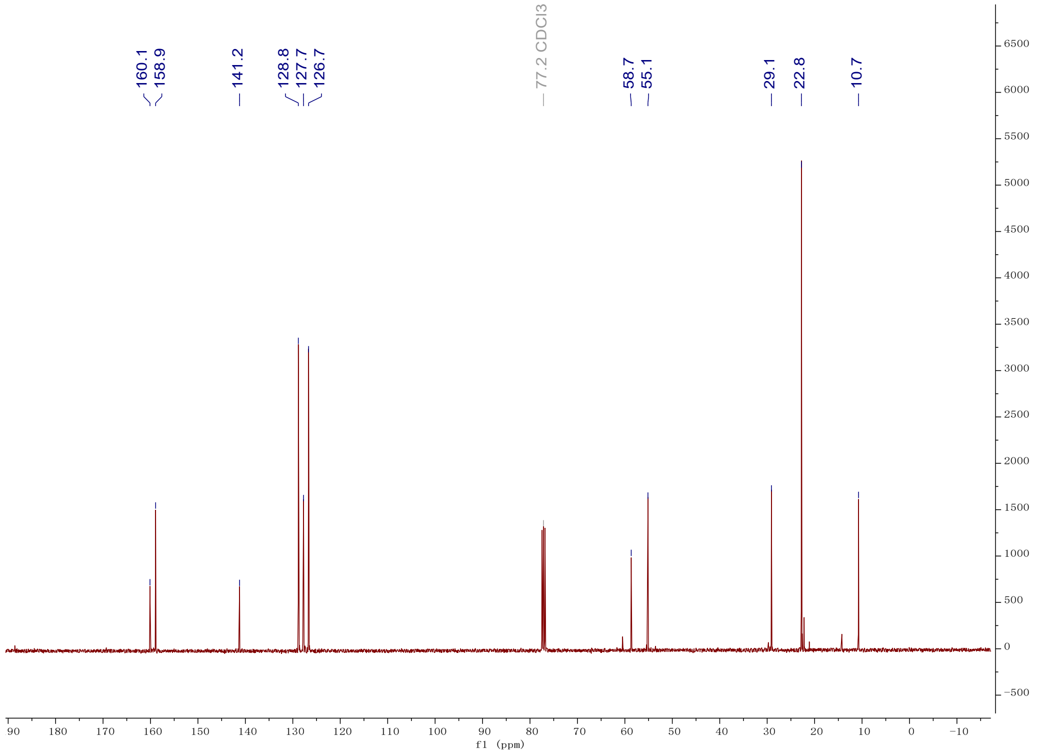

160.1
158.9
141.2
128.8
127.7
126.7
77.2 CDCl3
58.7
55.1
29.1
22.8
10.7
f1 (ppm)

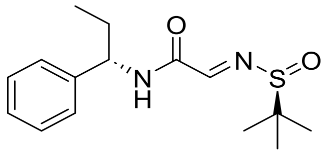
O
N
H
N
S
O

compound **2g**

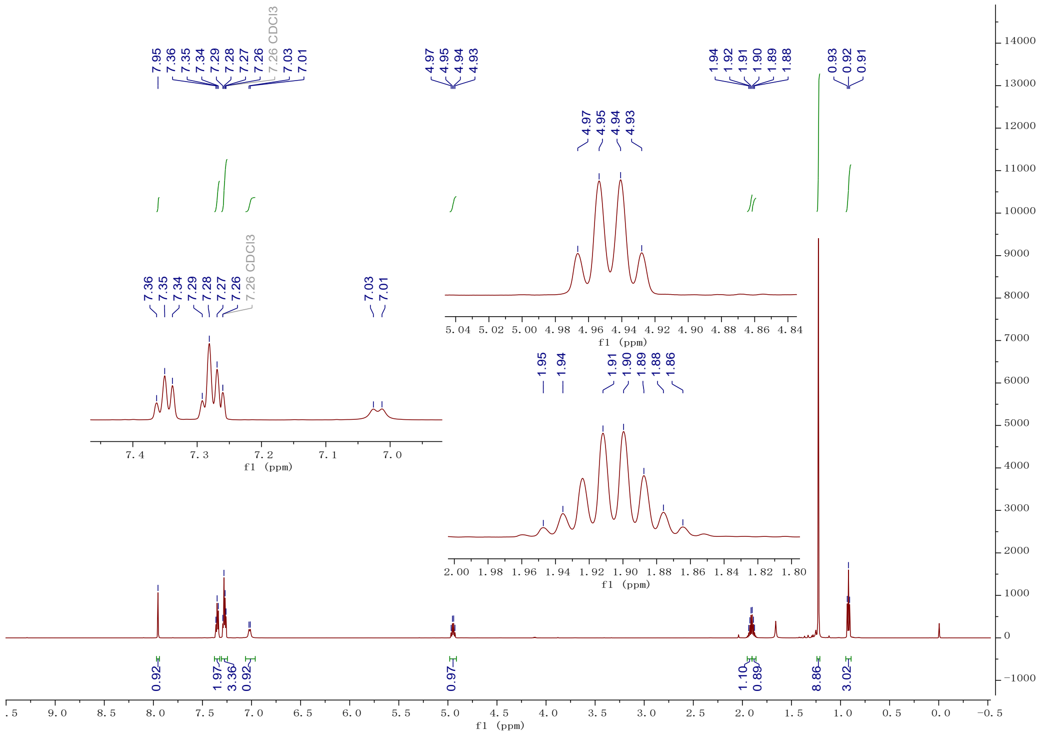
7.95
7.36
7.35
7.34
7.29
7.28
7.27
7.26
7.26 CDCl3
7.03
7.01
4.97
4.95
4.94
4.93
1.94
1.92
1.91
1.90
1.89
1.88
0.93
0.92
0.91
1.95
1.94
1.86
0.92
1.97
3.36
0.92
0.97
1.10
0.89
8.86
3.02
f1 (ppm)

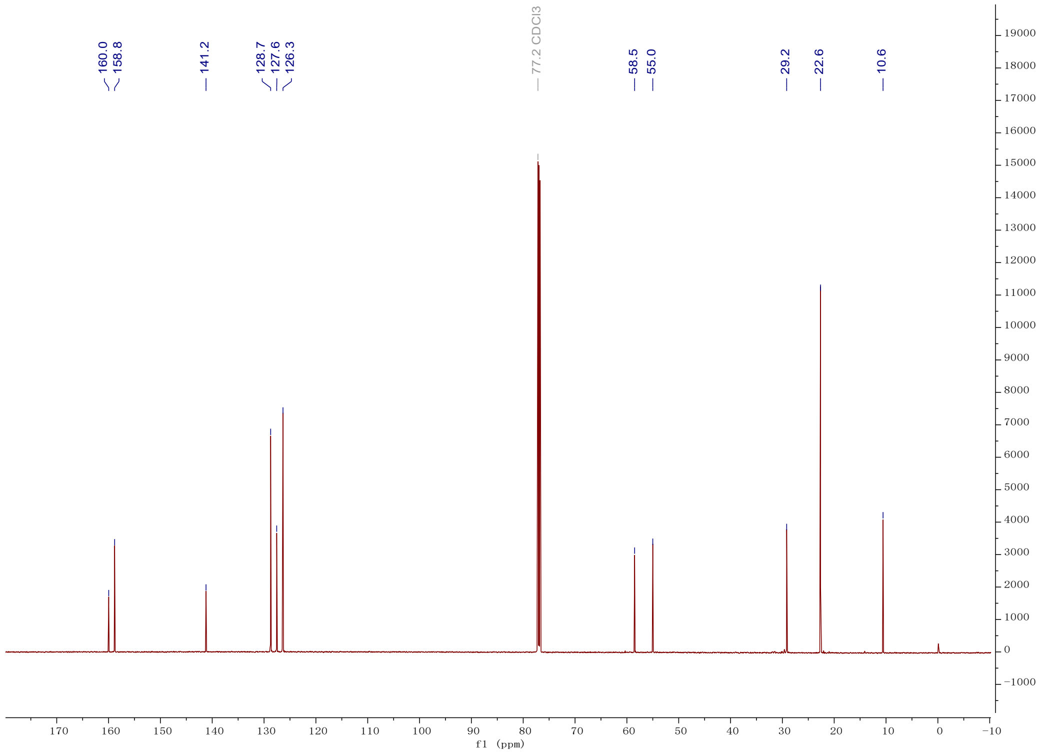
160.0
158.8
141.2
128.7
127.6
126.3
77.2 CDCl3
58.5
55.0
29.2
22.6
10.6
f1 (ppm)

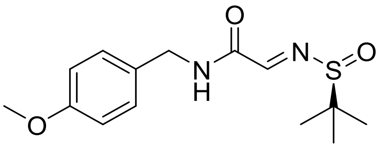
O
N
H
N
S
O
O

compound **2h**

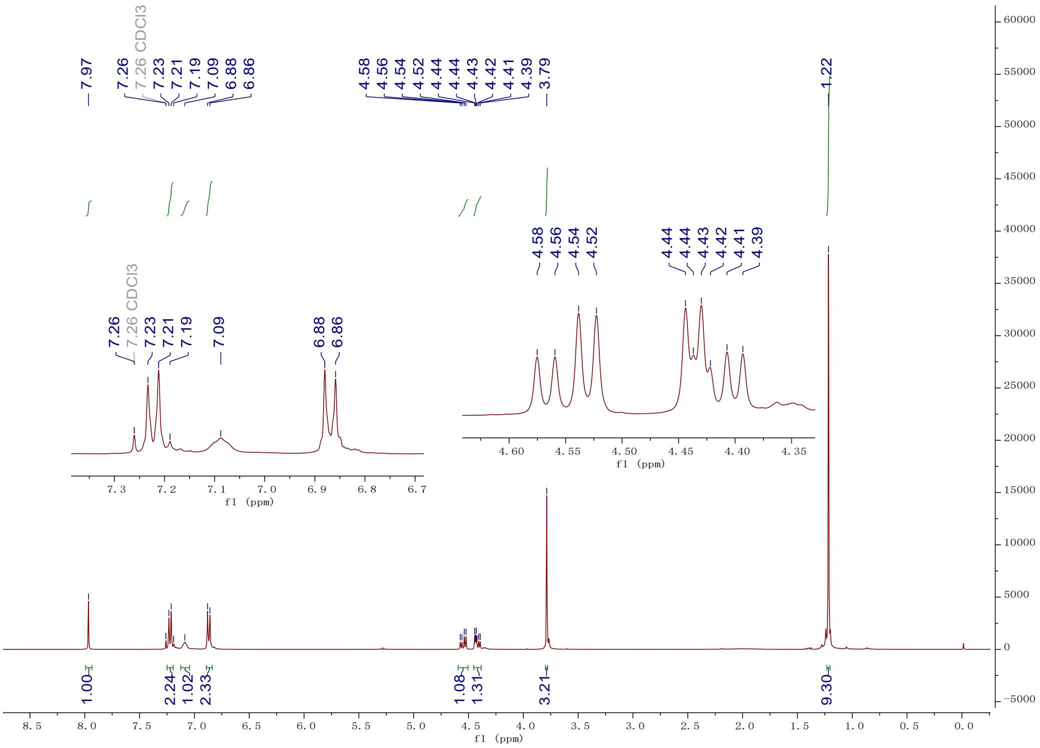
7.97
7.26
7.26 CDCl3
7.23
7.21
7.19
7.09
6.88
6.86
4.58
4.56
4.54
4.52
4.44
4.44
4.43
4.42
4.41
4.39
3.79
1.22
1.00
2.24
1.02
2.33
1.08
1.31
3.21
9.30
f1 (ppm)

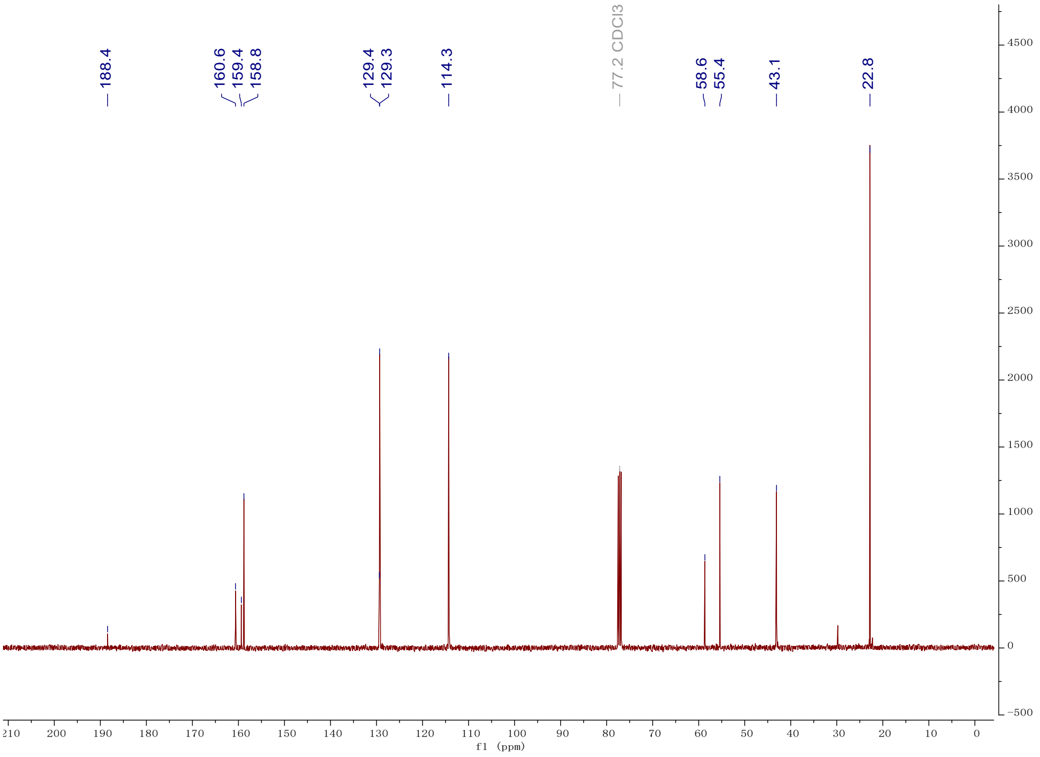
188.4
160.6
159.4
158.8
129.4
129.3
114.3
77.2 CDCl3
58.6
55.4
43.1
22.8
f1 (ppm)

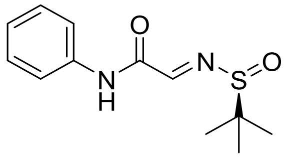
O
N
H
N
S
O

compound **2i**

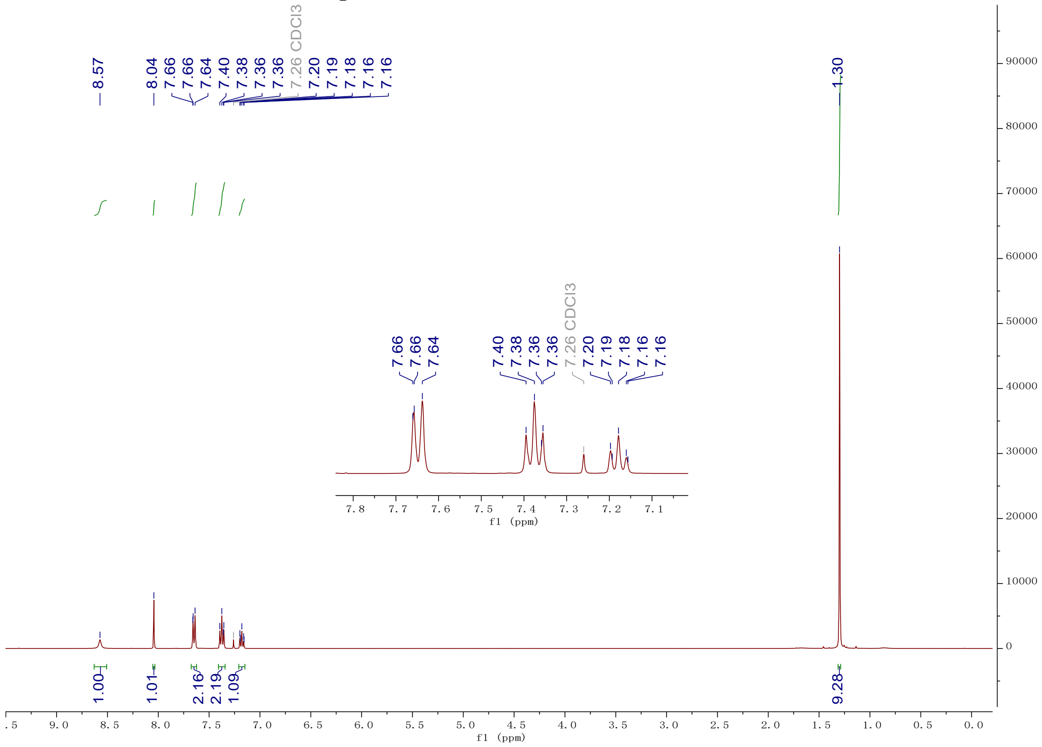
8.57
8.04
7.66
7.66
7.64
7.40
7.38
7.36
7.36
7.26 CDCl3
7.20
7.19
7.18
7.16
7.16
1.30
1.00
1.01
2.16
2.19
1.09
9.28
f1 (ppm)

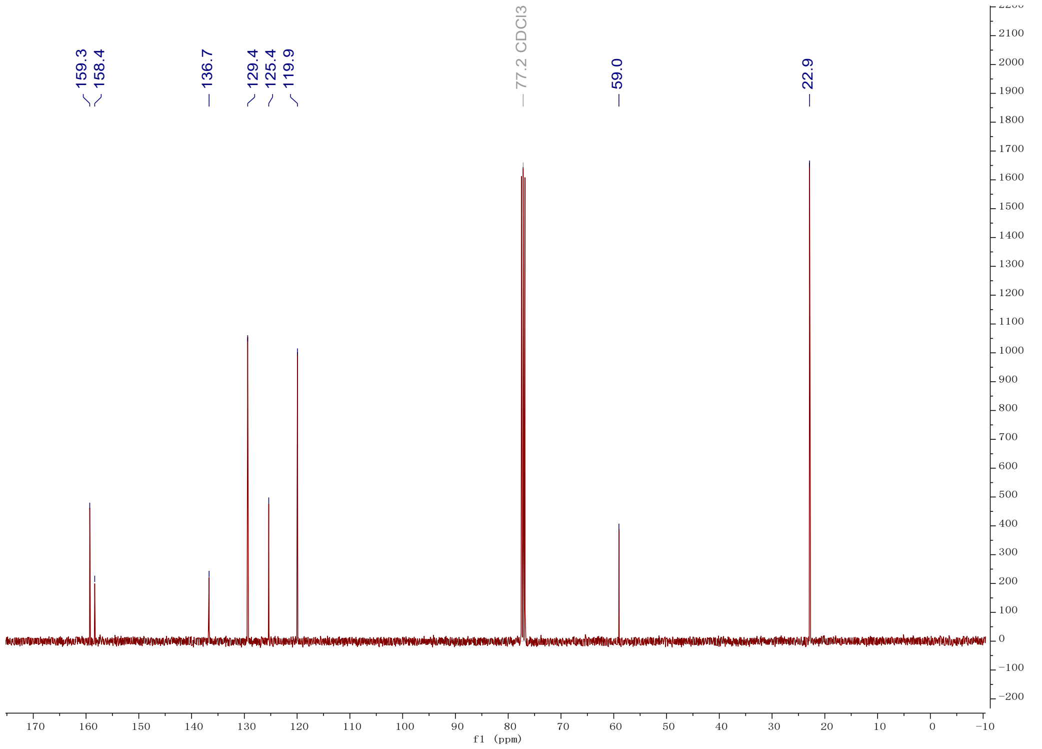
159.3
158.4
136.7
129.4
125.4
119.9
77.2 CDCl3
59.0
22.9
f1 (ppm)

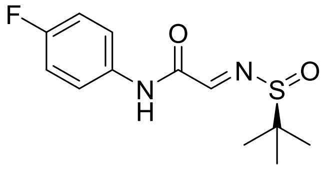
F
O
N
S
O
N
H

compound **2j**

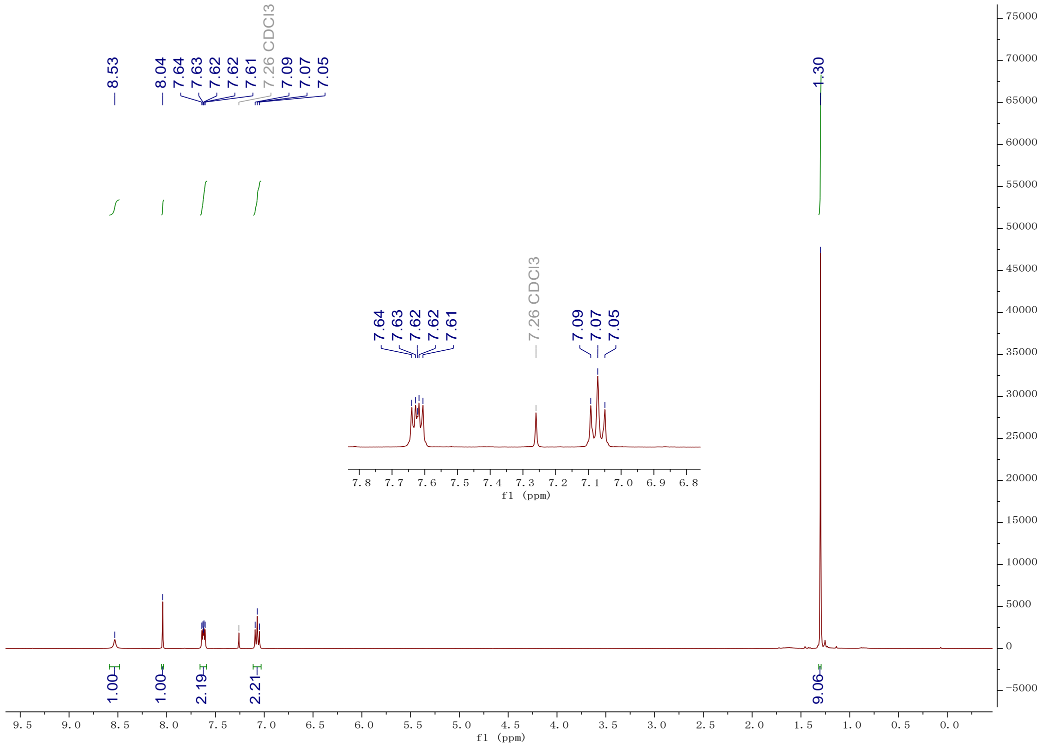
8.53
8.04
7.64
7.63
7.62
7.62
7.61
7.26 CDCl3
7.09
7.07
7.05
1.30
1.00
1.00
2.19
2.21
9.06
f1 (ppm)

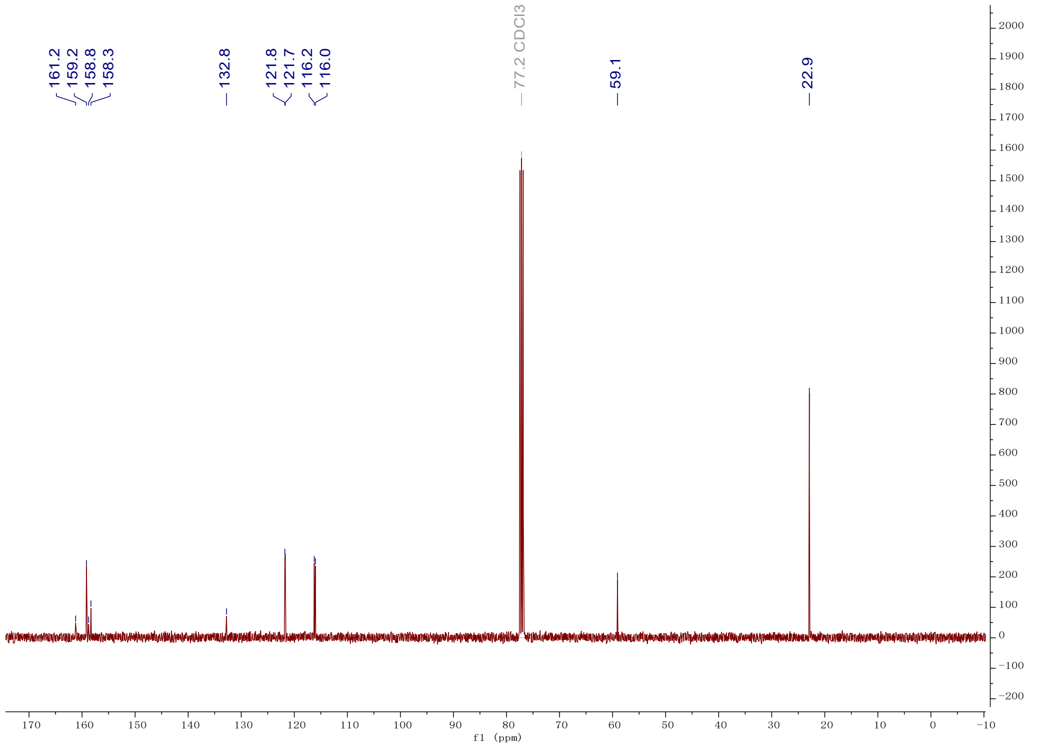
161.2
159.2
158.8
158.3
132.8
121.8
121.7
116.2
116.0
77.2 CDCl3
59.1
22.9
f1 (ppm)

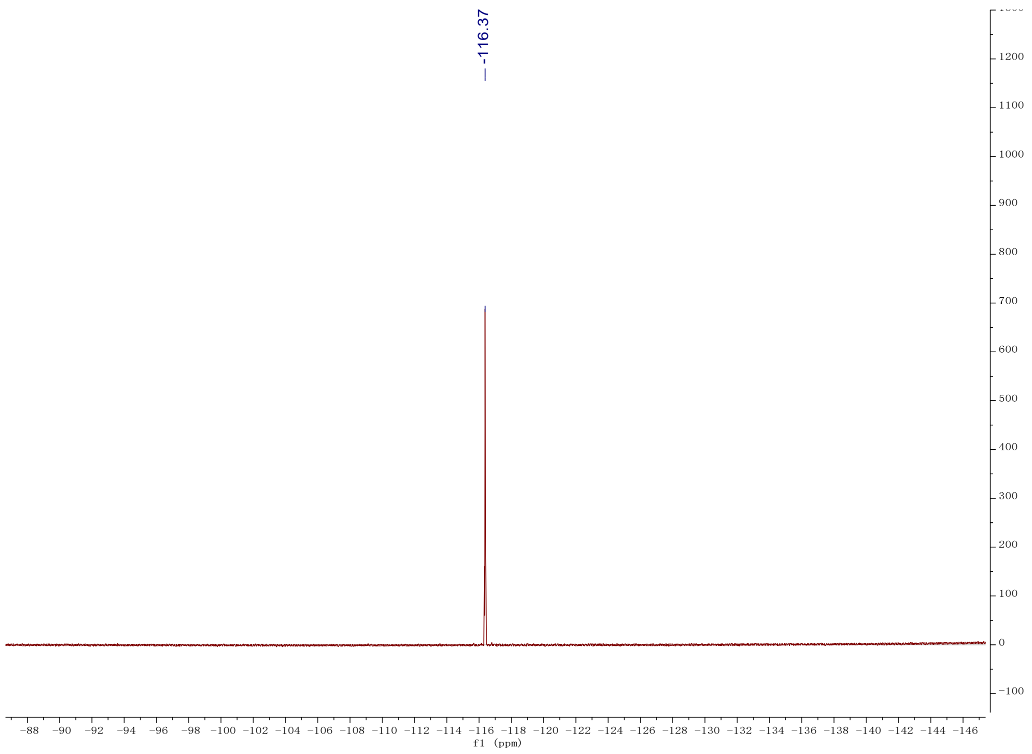
-116.37
f1 (ppm)

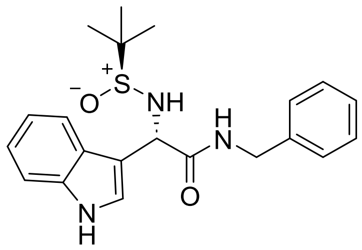
NH
H
N
O
NH
H
S
O

compound 3

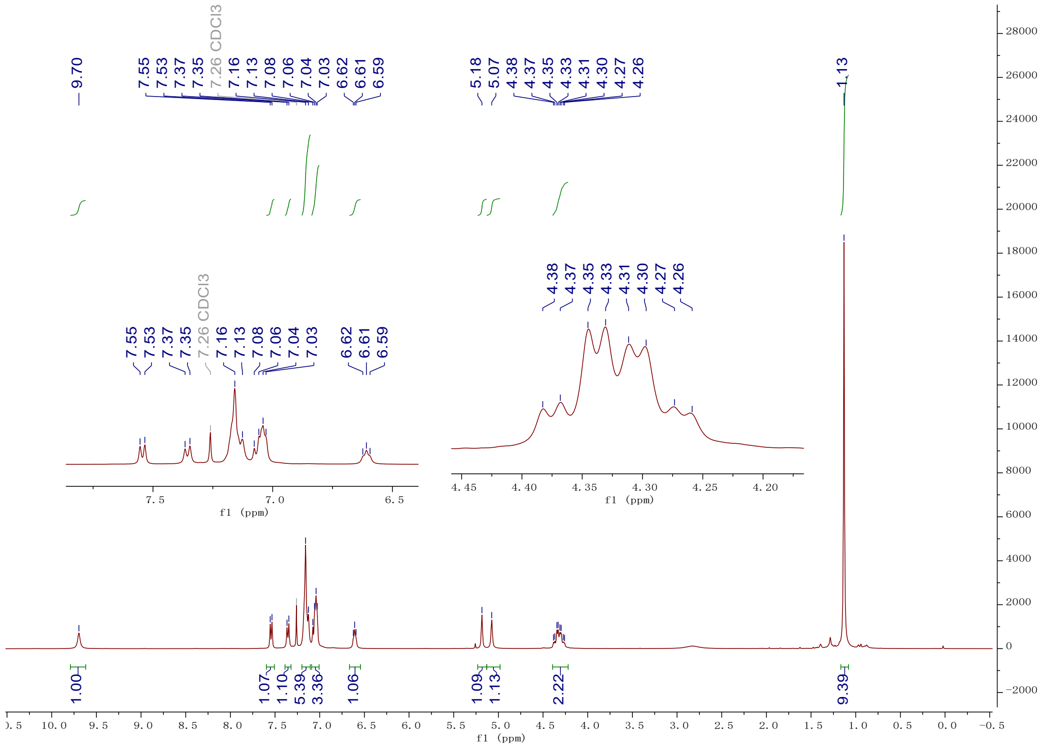
9.70
7.55
7.53
7.37
7.35
7.26 CDCl3
7.16
7.13
7.08
7.06
7.04
7.03
6.62
6.61
6.59
5.18
5.07
4.38
4.37
4.35
4.33
4.31
4.30
4.27
4.26
1.13
1.00
1.07
1.10
5.39
3.36
1.06
1.09
1.13
2.22
9.39
f1 (ppm)

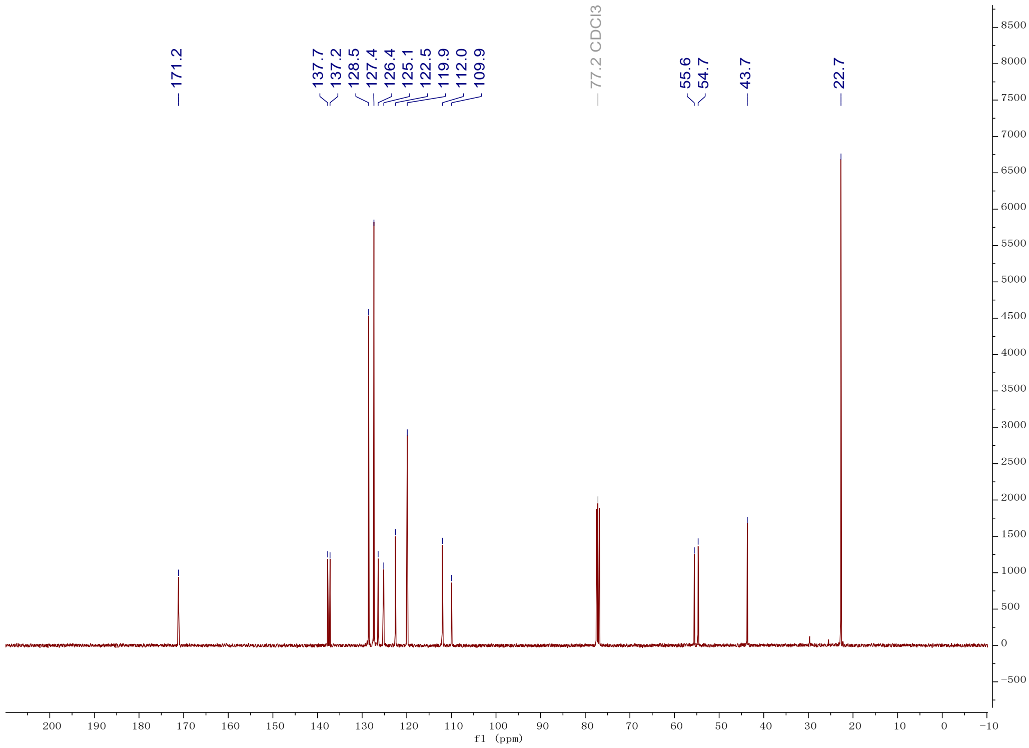
171.2
137.7
137.2
128.5
127.4
126.4
125.1
122.5
119.9
112.0
109.9
77.2 CDCl3
55.6
54.7
43.7
22.7
f1 (ppm)

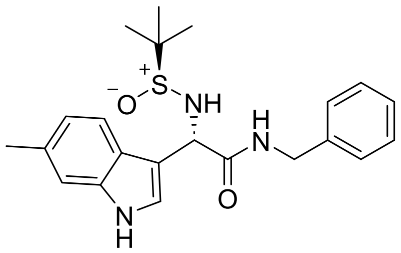
S
NH
O
H
N
N
H
O

compound **4**

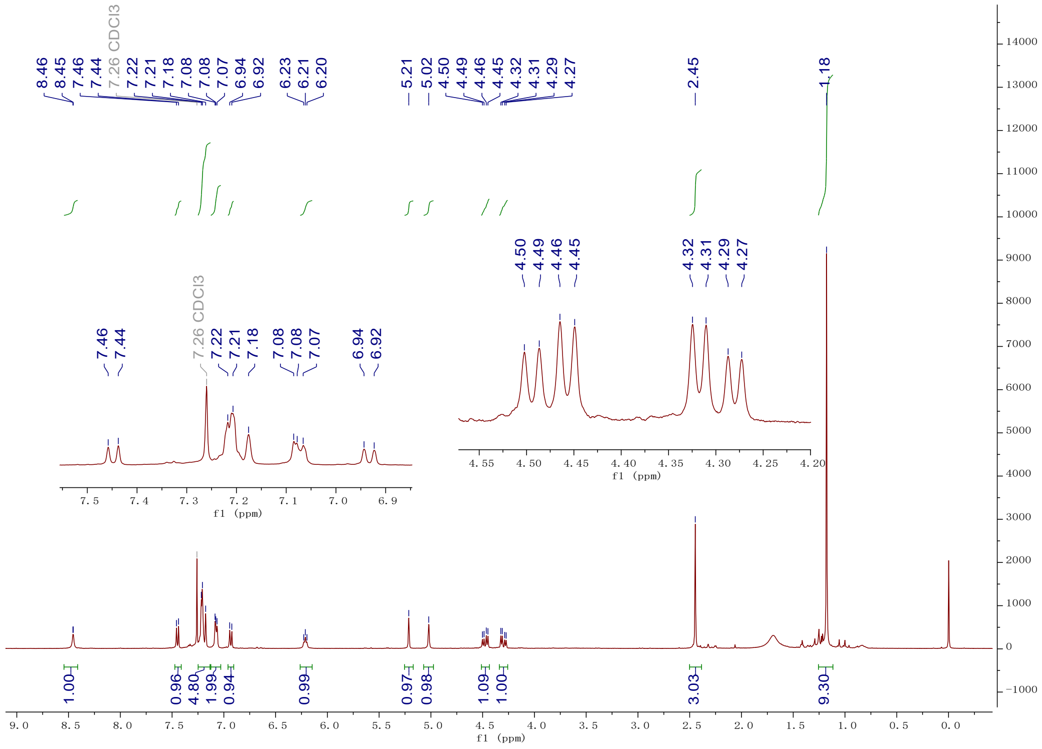
8.46
8.45
7.46
7.44
7.26 CDCl3
7.22
7.21
7.18
7.08
7.08
7.07
6.94
6.92
6.23
6.21
6.20
5.21
5.02
4.50
4.49
4.46
4.45
4.32
4.31
4.29
4.27
2.45
1.18
1.00
0.96
4.80
1.99
0.94
0.99
0.97
0.98
1.09
1.00
3.03
9.30
f1 (ppm)

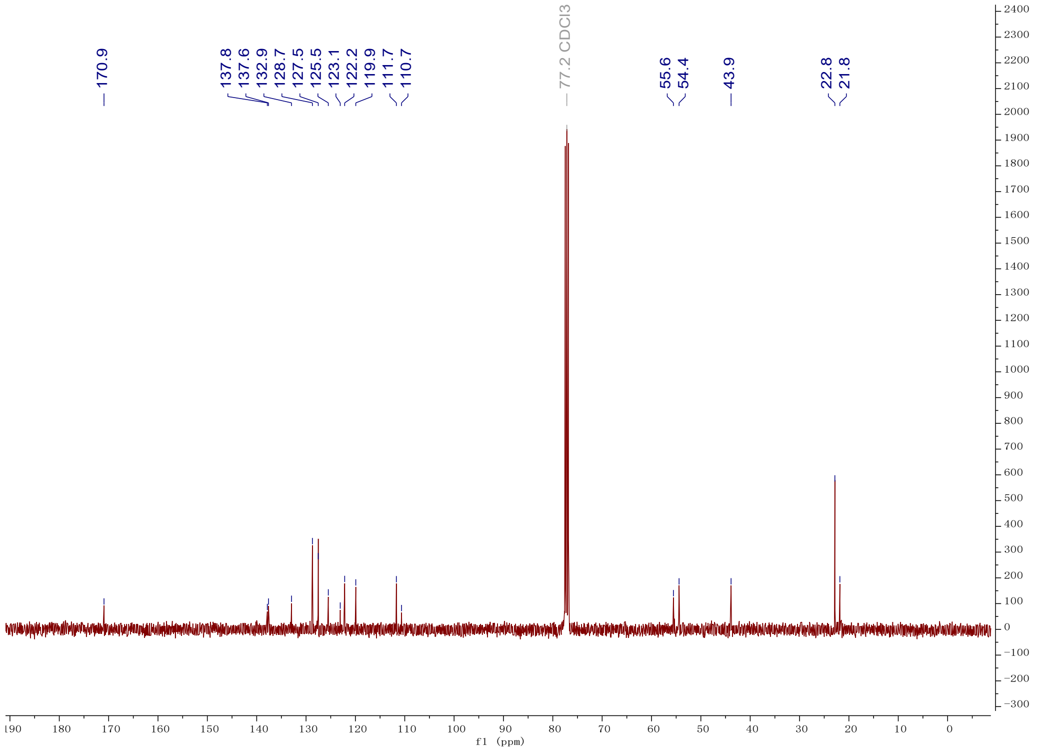
170.9
137.8
137.6
132.9
128.7
127.5
125.5
123.1
122.2
119.9
111.7
110.7
77.2 CDCl3
55.6
54.4
43.9
22.8
21.8
f1 (ppm)

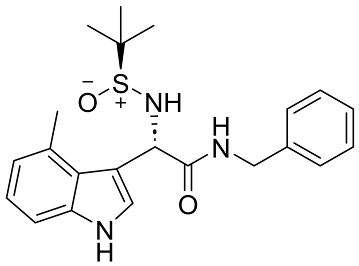
O
S
NH
H
N
O
N
H

compound 5

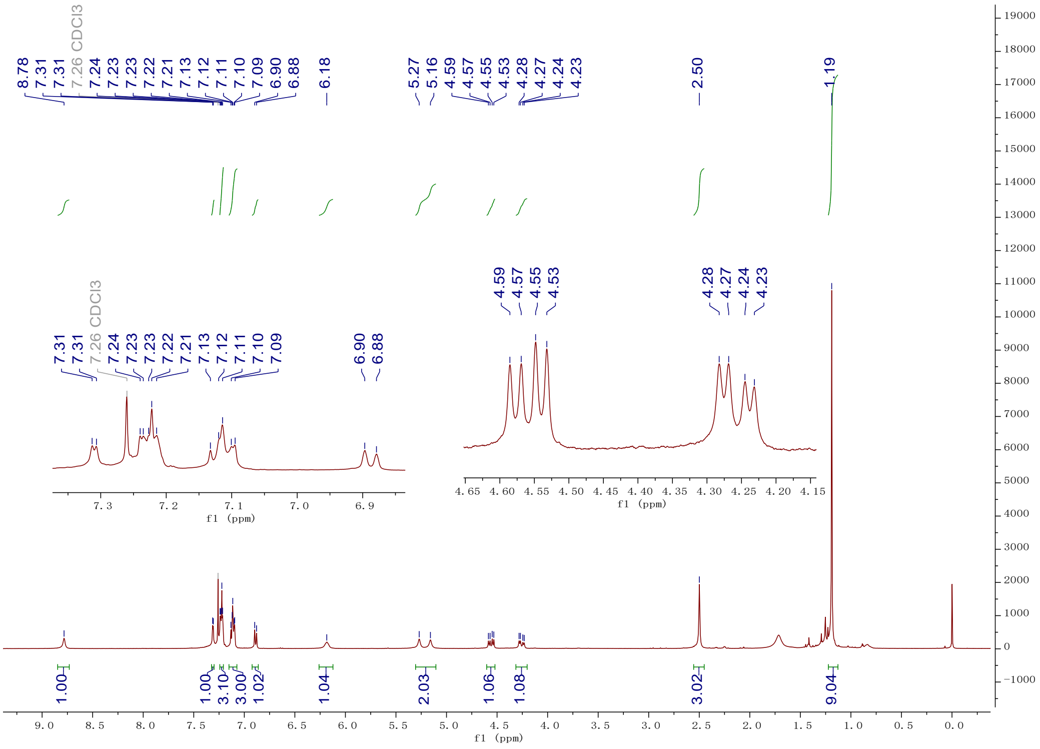
8.78 7.31 7.31 7.26 CDCl3 7.24 7.23 7.23 7.22 7.21 7.21 7.13 7.12 7.11 7.10 7.09 6.90 6.88 6.18 5.27 5.16 4.59 4.57 4.55 4.53 4.28 4.27 4.24 4.23 2.50 1.19
1.00 1.00 3.10 3.00 1.02 1.04 2.03 1.06 1.08 3.02 9.04
f1 (ppm)

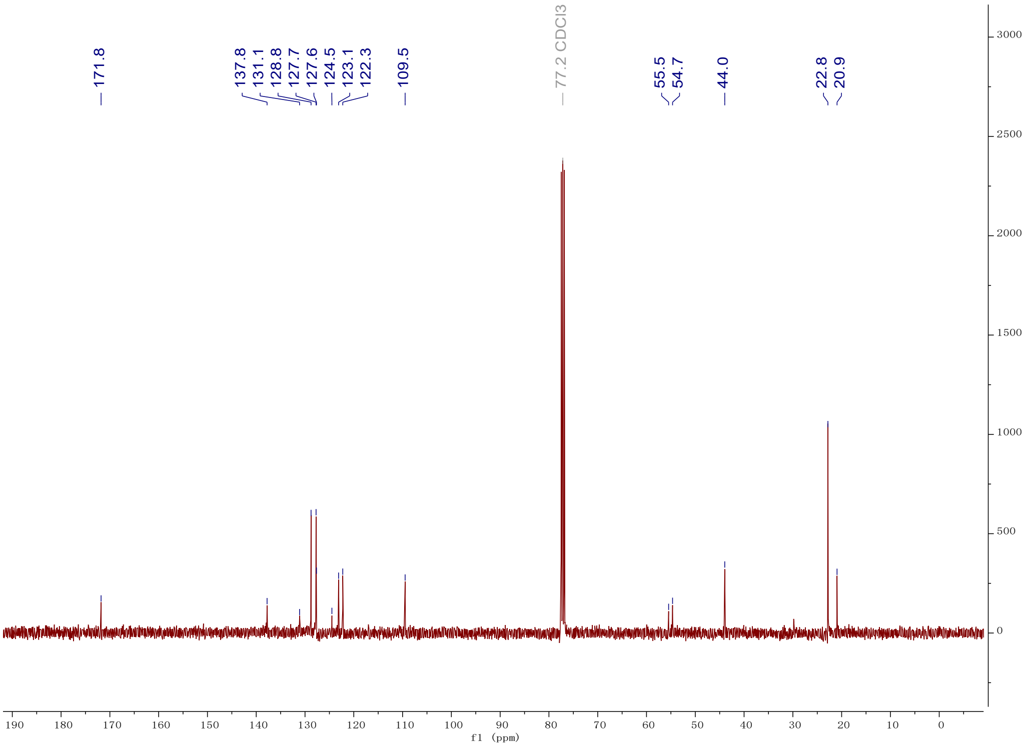
171.8 137.8 131.1 128.8 127.7 127.6 124.5 123.1 122.3 109.5 77.2 CDCl3 55.5 54.7 44.0 22.8 20.9
f1 (ppm)

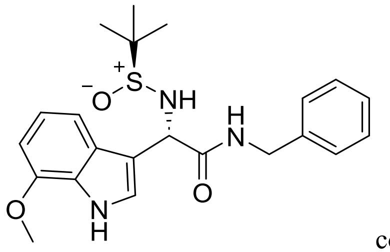

compound **6**

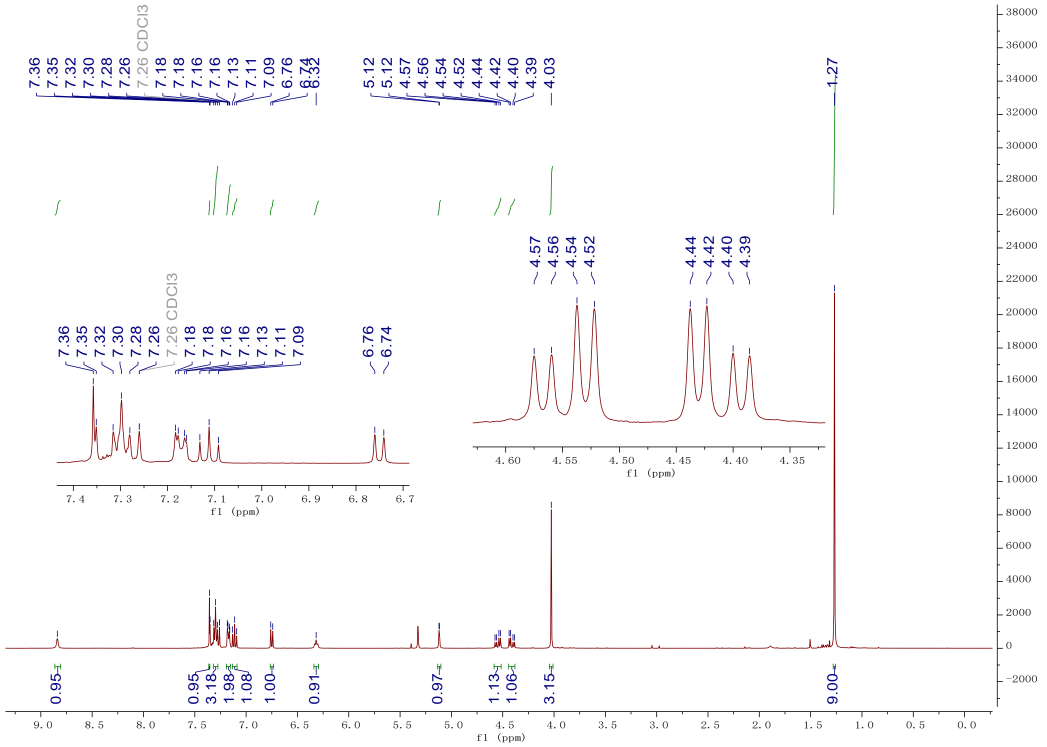

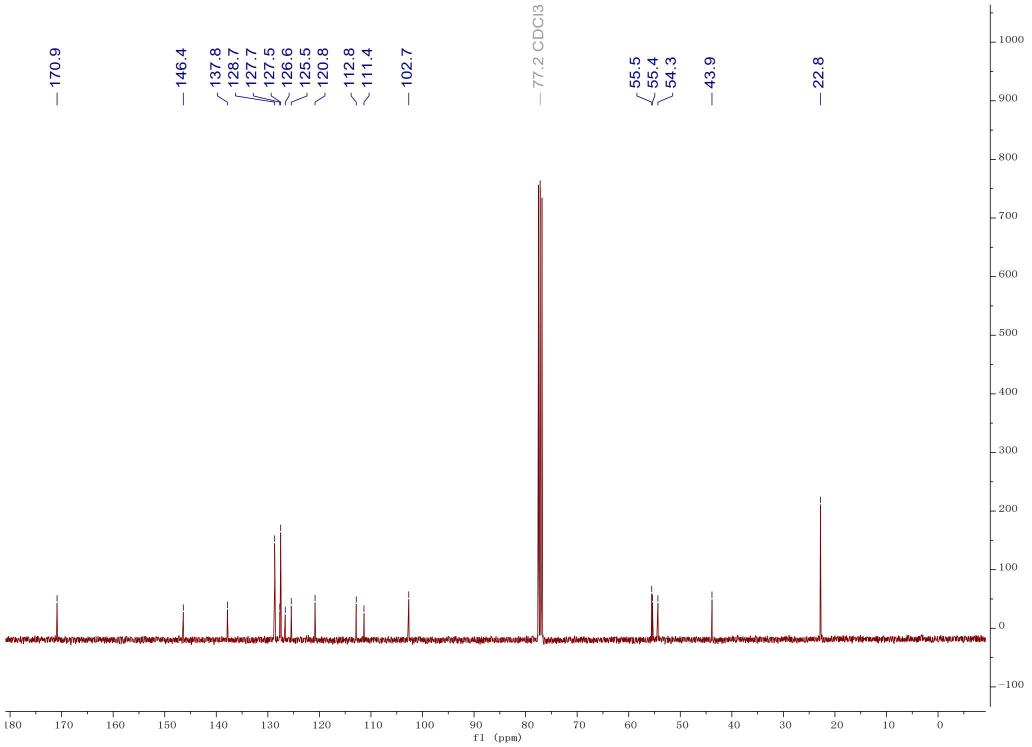

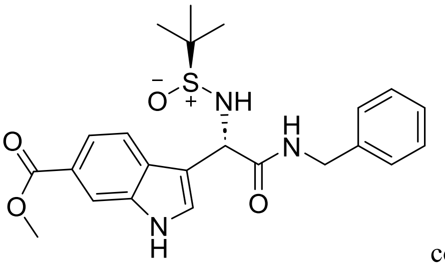

compound **7**

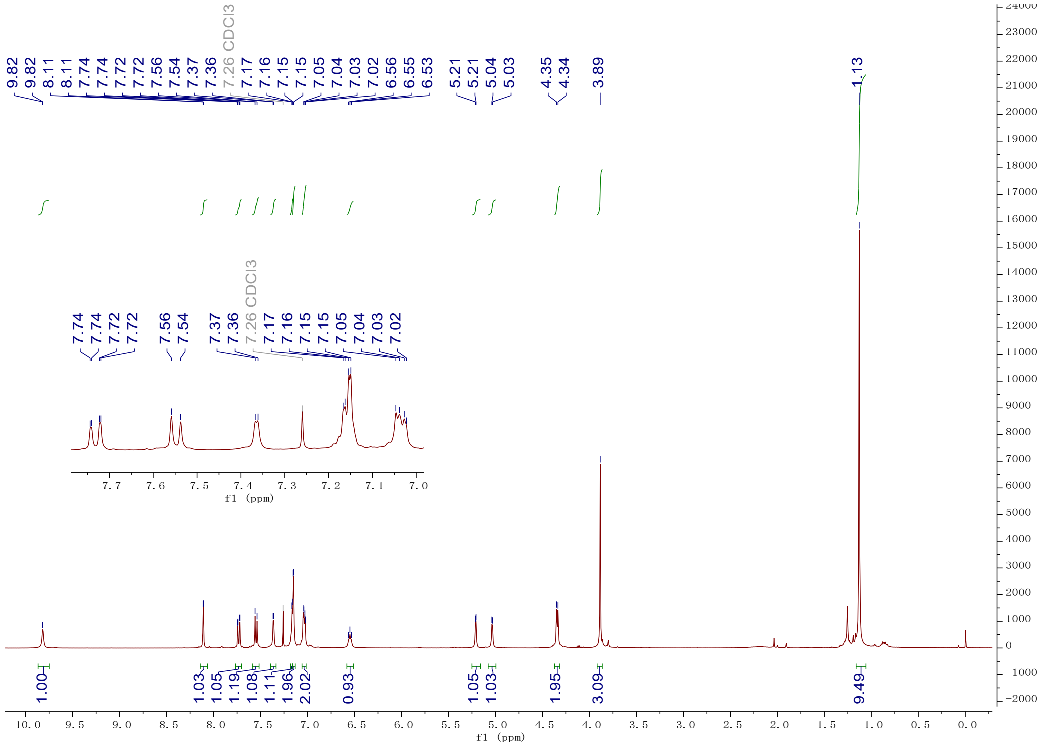

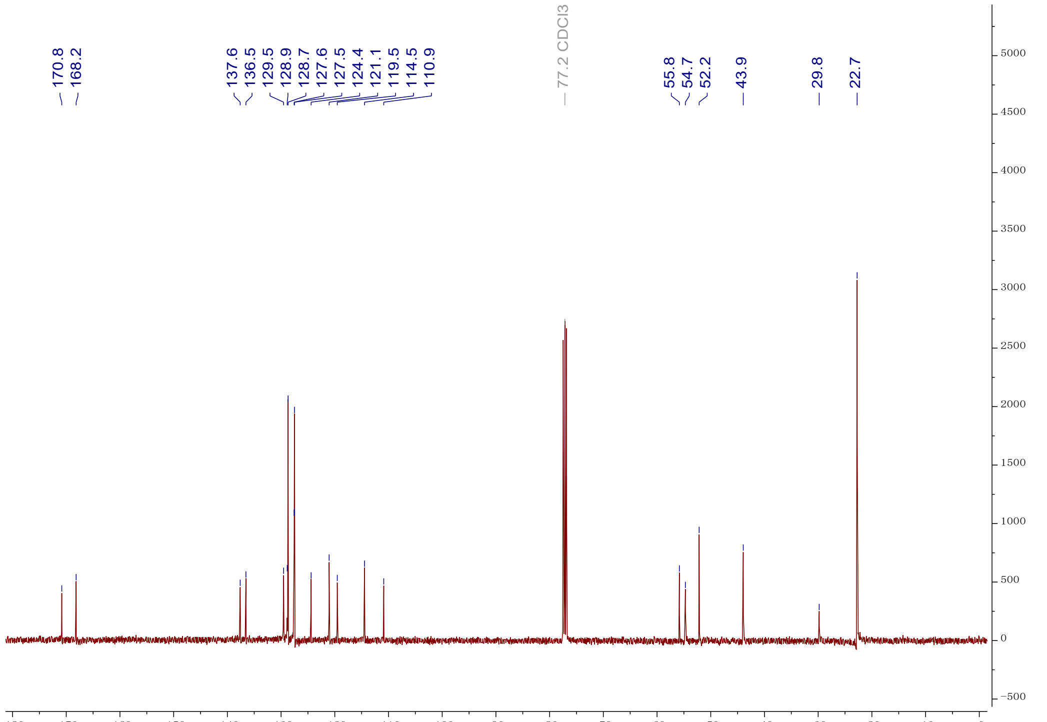

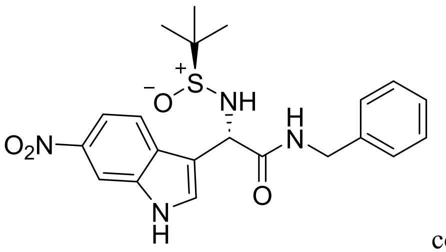
O2N
NH
S
O
H
N
O
N
H

compound **8**

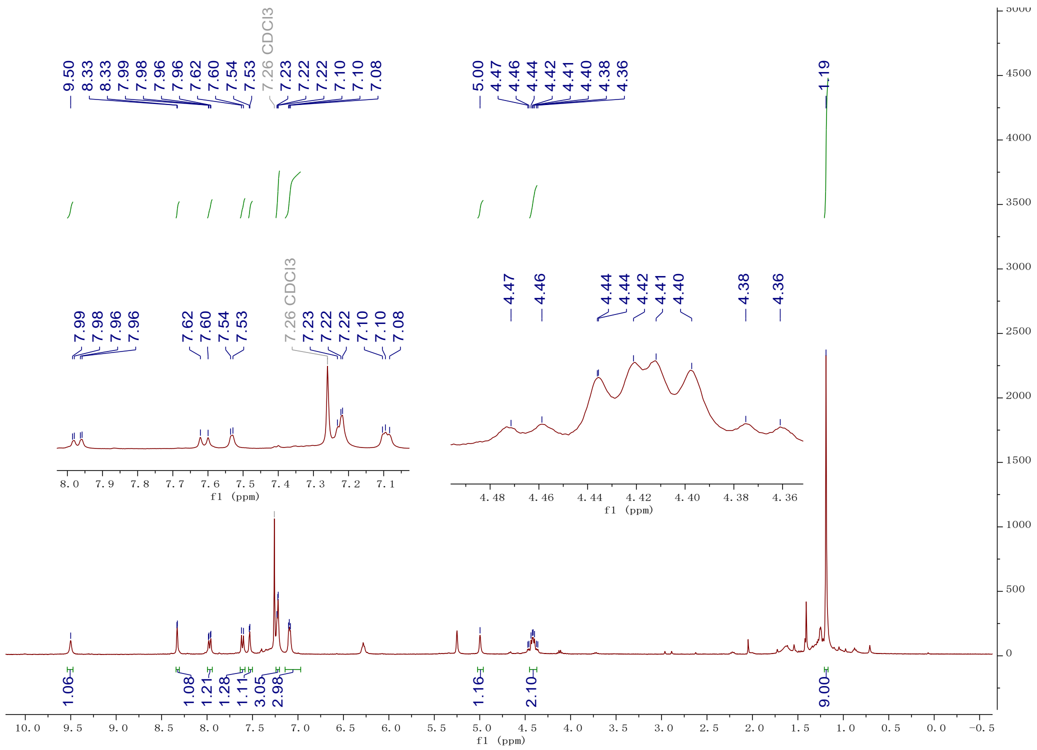
9.50
8.33
8.33
7.99
7.98
7.96
7.96
7.62
7.60
7.54
7.53
7.26 CDCl3
7.23
7.22
7.22
7.22
7.10
7.10
7.08
5.00
4.47
4.46
4.44
4.44
4.42
4.41
4.40
4.38
4.36
1.19
1.06
1.08
1.21
1.28
1.11
3.05
2.98
1.16
2.10
9.00
f1 (ppm)

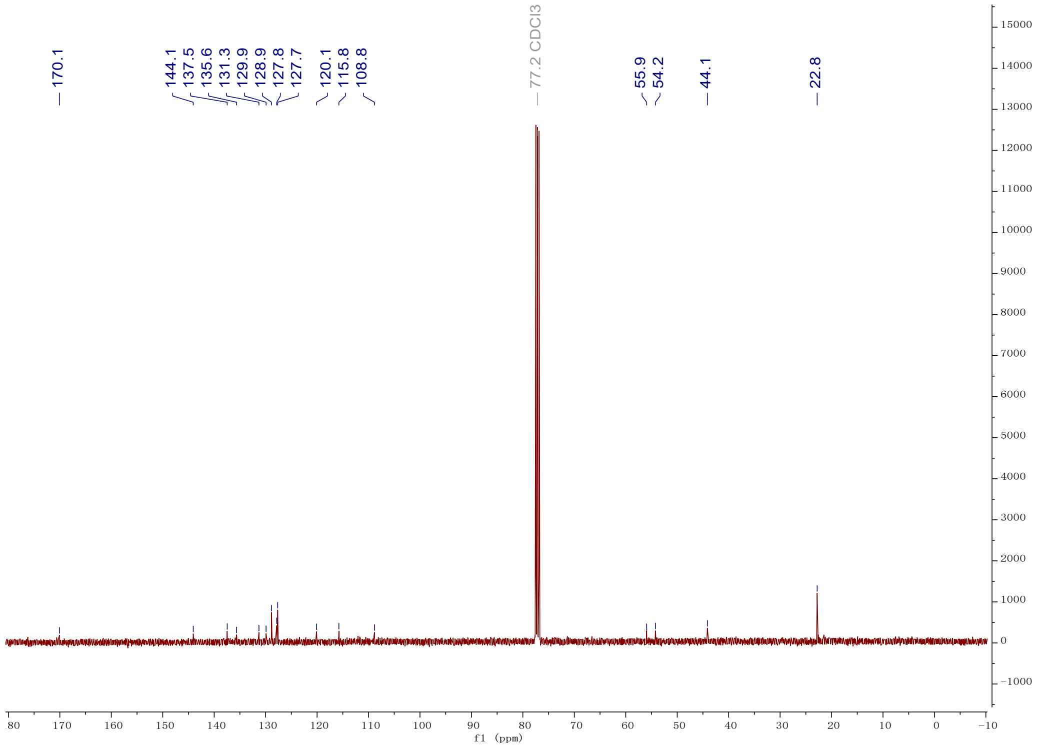
170.1
144.1
137.5
135.6
131.3
129.9
128.9
127.8
127.7
120.1
115.8
108.8
77.2 CDCl3
55.9
54.2
44.1
22.8
f1 (ppm)

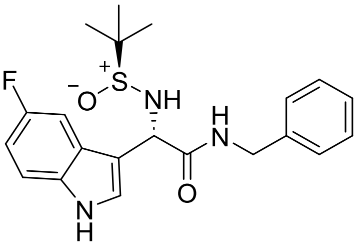
F
O
S
NH
H
N
O
N
H

compound **9**

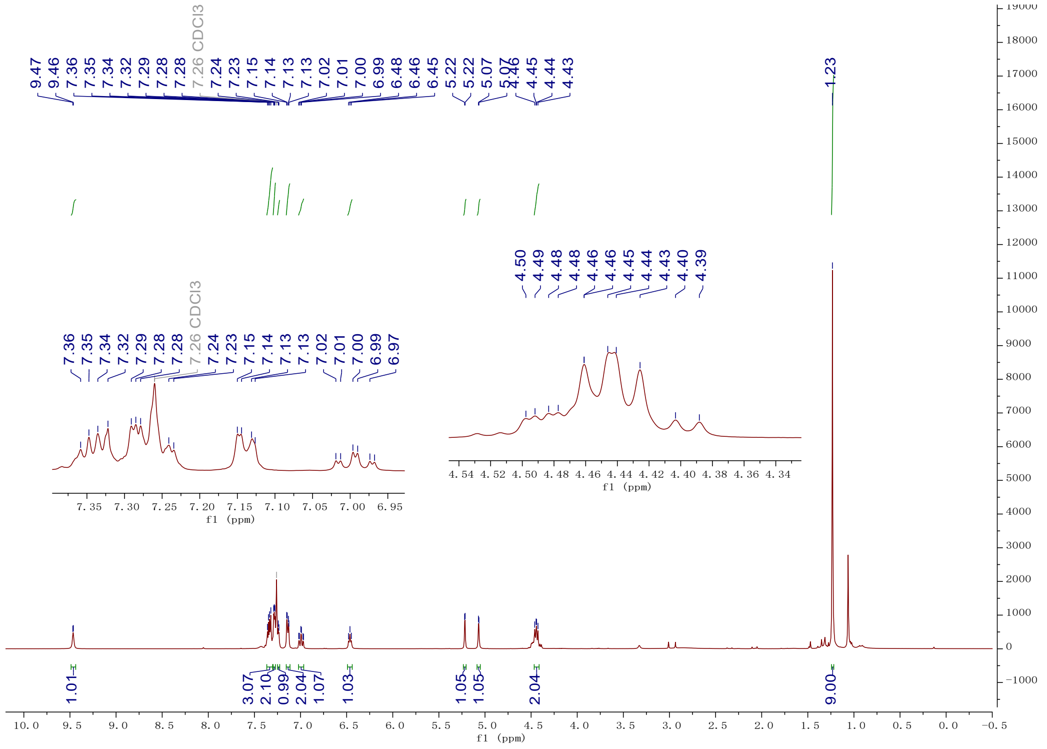
9.47
9.46
7.36
7.35
7.34
7.32
7.29
7.28
7.28
7.26 CDCl3
7.24
7.23
7.15
7.14
7.13
7.13
7.02
7.01
7.00
6.99
6.48
6.46
6.45
5.22
5.22
5.07
5.07
4.46
4.45
4.44
4.43
1.23
1.01
3.07
2.10
0.99
2.04
1.07
1.03
1.05
1.05
2.04
9.00
f1 (ppm)

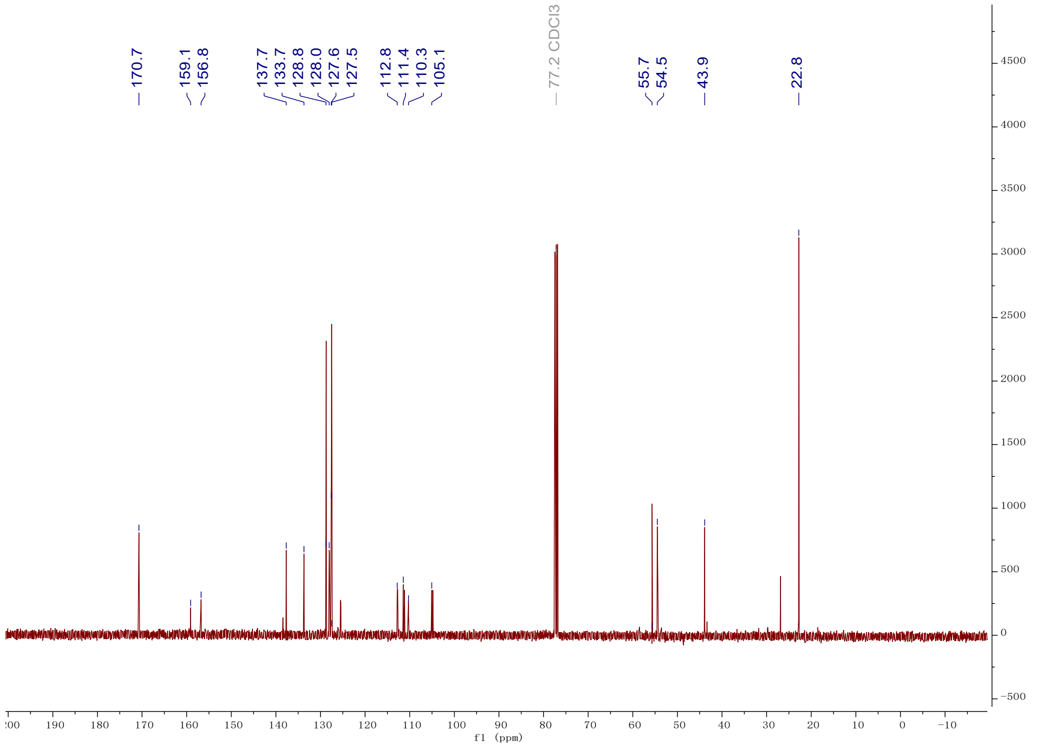
170.7
159.1
156.8
137.7
133.7
128.8
128.0
127.6
127.5
112.8
111.4
110.3
105.1
77.2 CDCl3
55.7
54.5
43.9
22.8
f1 (ppm)

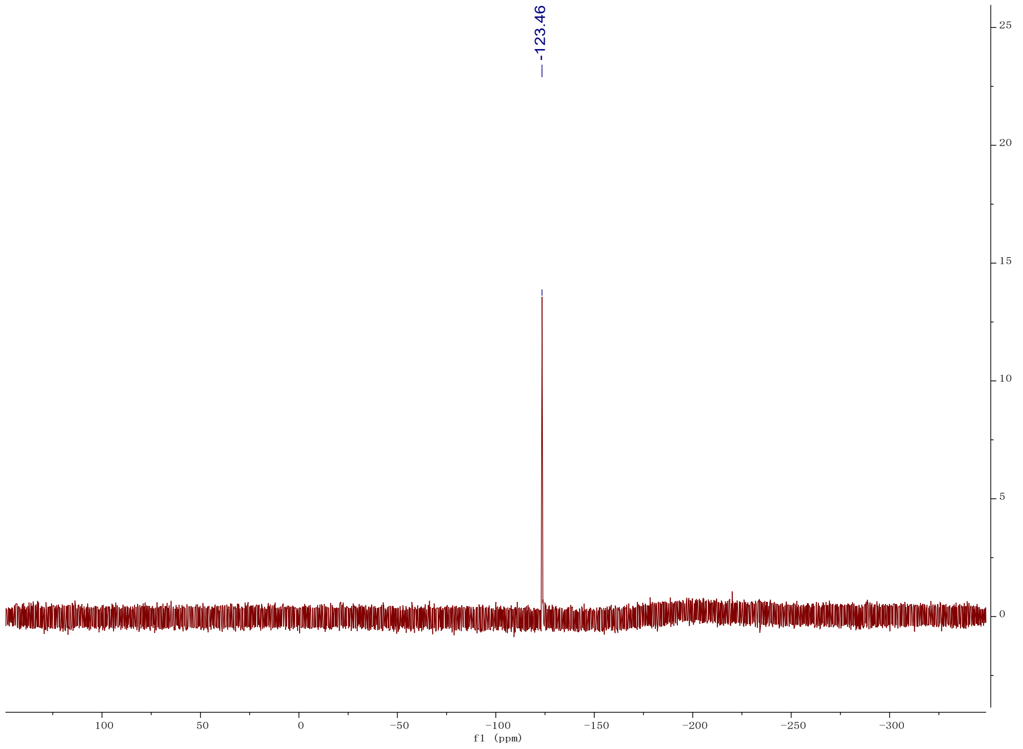
-123.46
25
20
15
10
5
0
100
50
0
-50
-100
-150
-200
-250
-300
f1 (ppm)

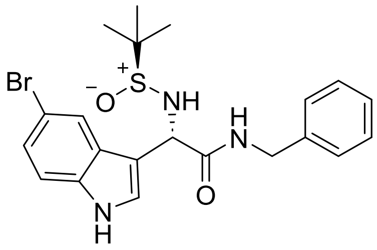
Br
O
S
NH
H
N
O
N
H

compound **10**

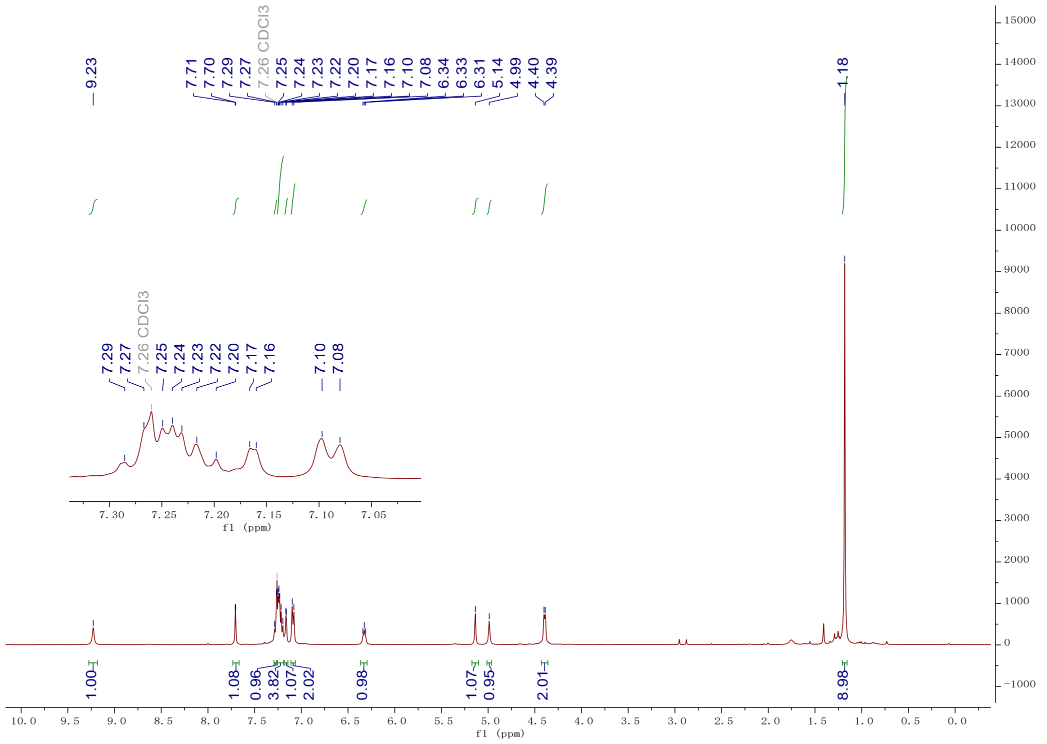
9.23
7.71 7.70 7.29 7.27 7.26 CDCl3 7.25 7.24 7.23 7.22 7.20 7.17 7.16 7.10 7.08 6.34 6.33 6.31 5.14 4.99 4.40 4.39
1.18
7.29 7.27 7.26 CDCl3 7.25 7.24 7.23 7.22 7.20 7.17 7.16 7.10 7.08
1.00 1.08 0.96 3.82 1.07 2.02 0.98 1.07 0.95 2.01 8.98
f1 (ppm)

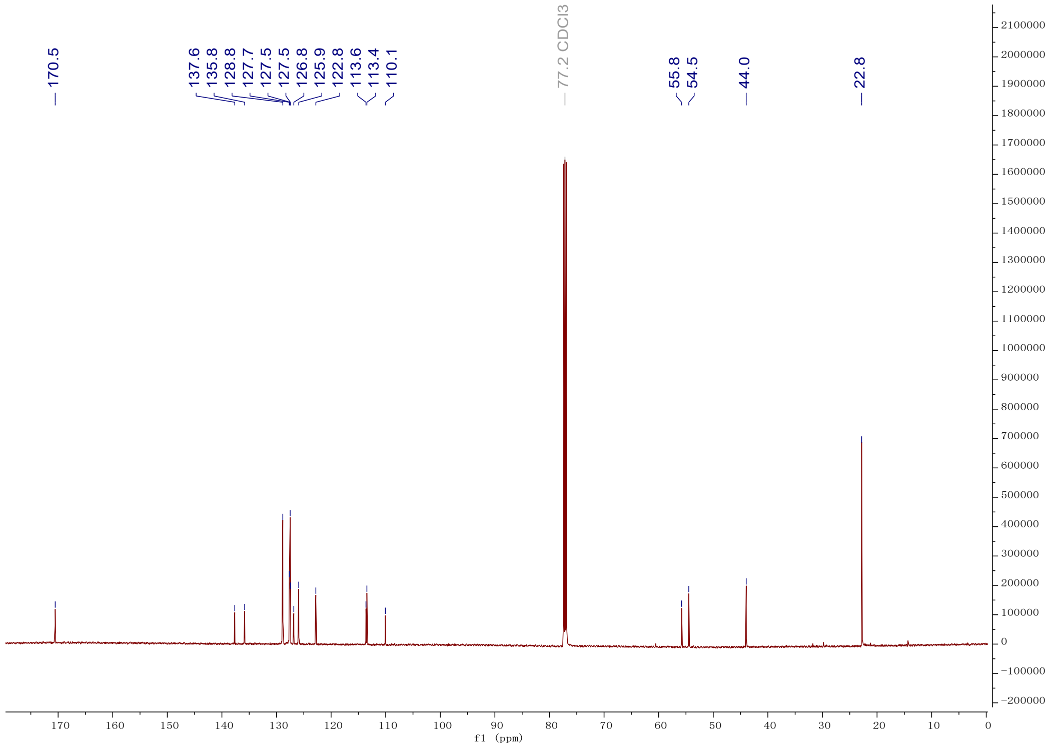
170.5
137.6 135.8 128.8 127.7 127.5 127.5 126.8 125.9 122.8 113.6 113.4 110.1
77.2 CDCl3
55.8 54.5
44.0
22.8
f1 (ppm)

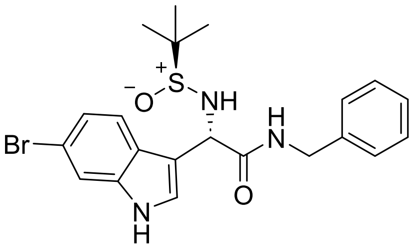

compound **11**

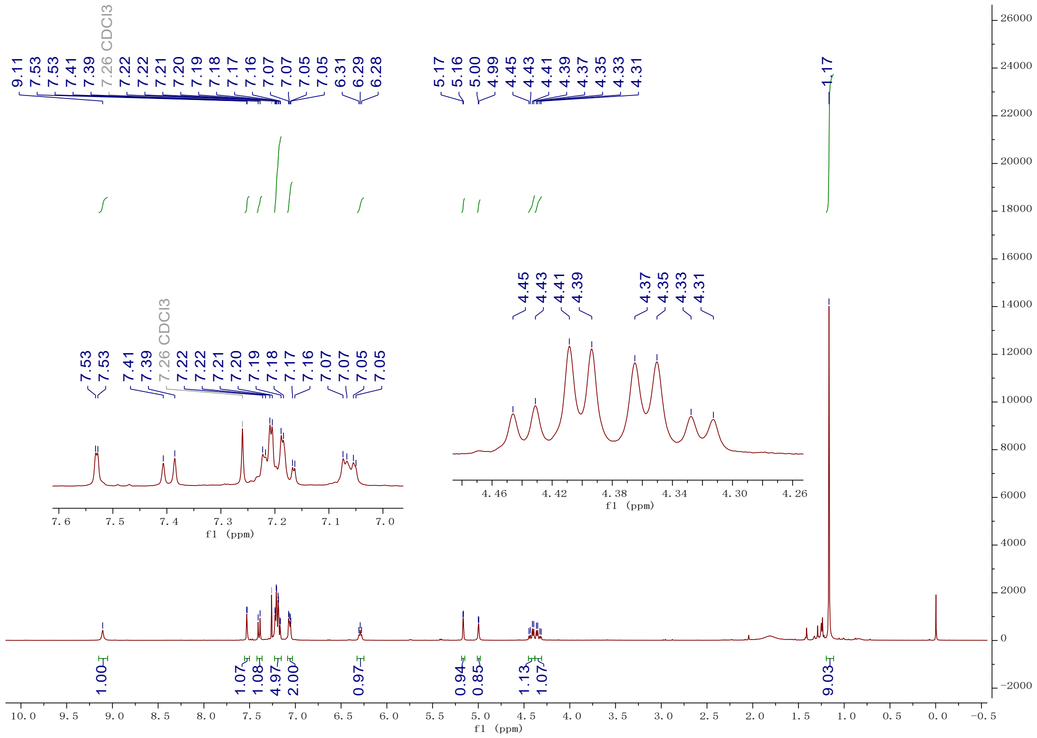

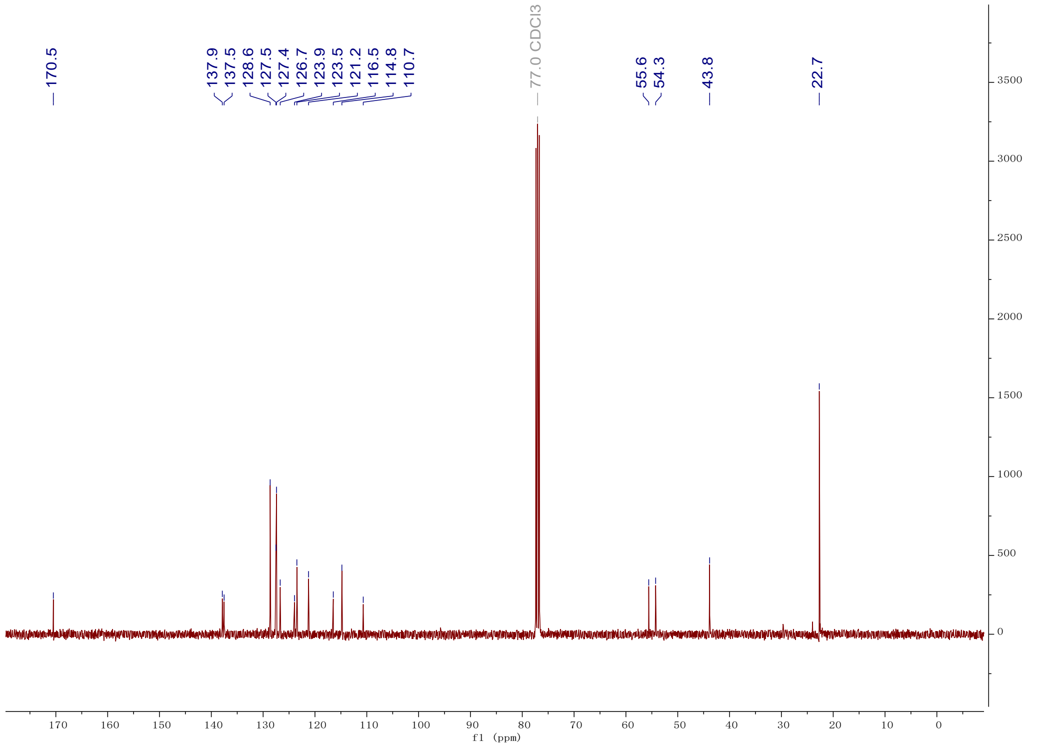

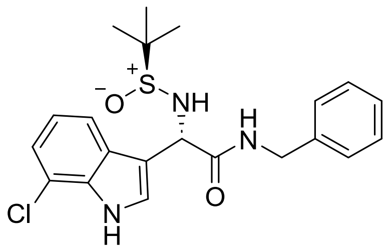
NH
H
N
O
Cl
N
H
O
S
+
–

compound **12**

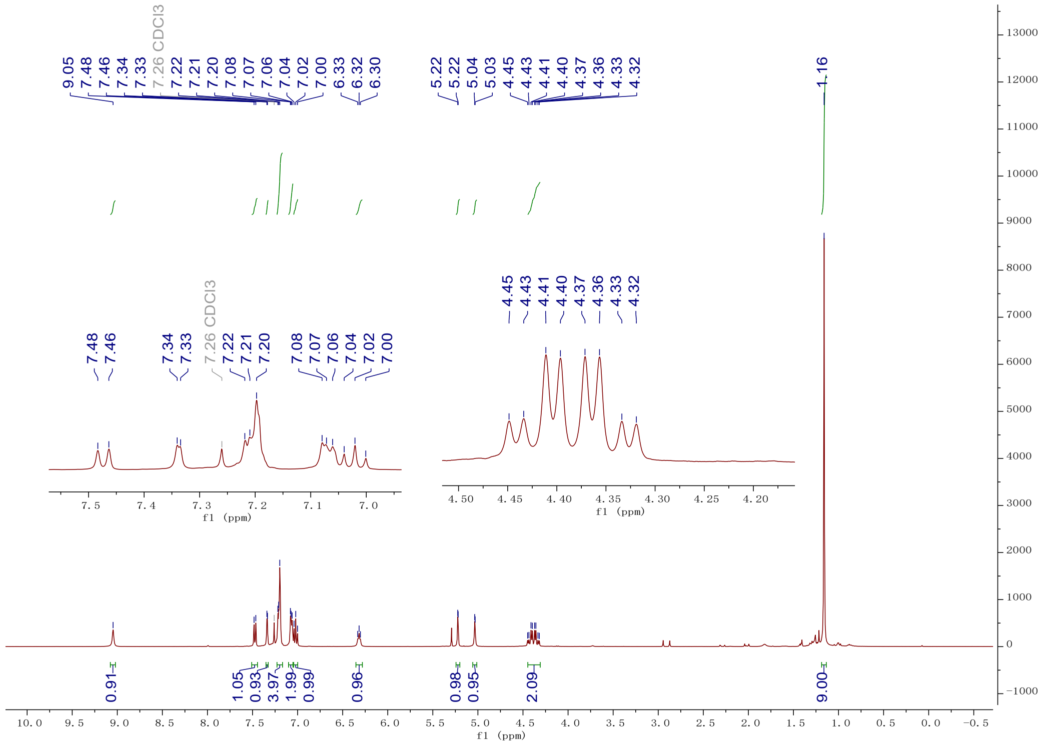
9.05
7.48
7.46
7.34
7.33
7.26 CDCl3
7.22
7.21
7.20
7.08
7.07
7.06
7.04
7.02
7.00
6.33
6.32
6.30
5.22
5.22
5.04
5.03
4.45
4.43
4.41
4.40
4.37
4.36
4.33
4.32
1.16
0.91
1.05
0.93
3.97
1.99
0.99
0.96
0.98
0.95
2.09
9.00
f1 (ppm)

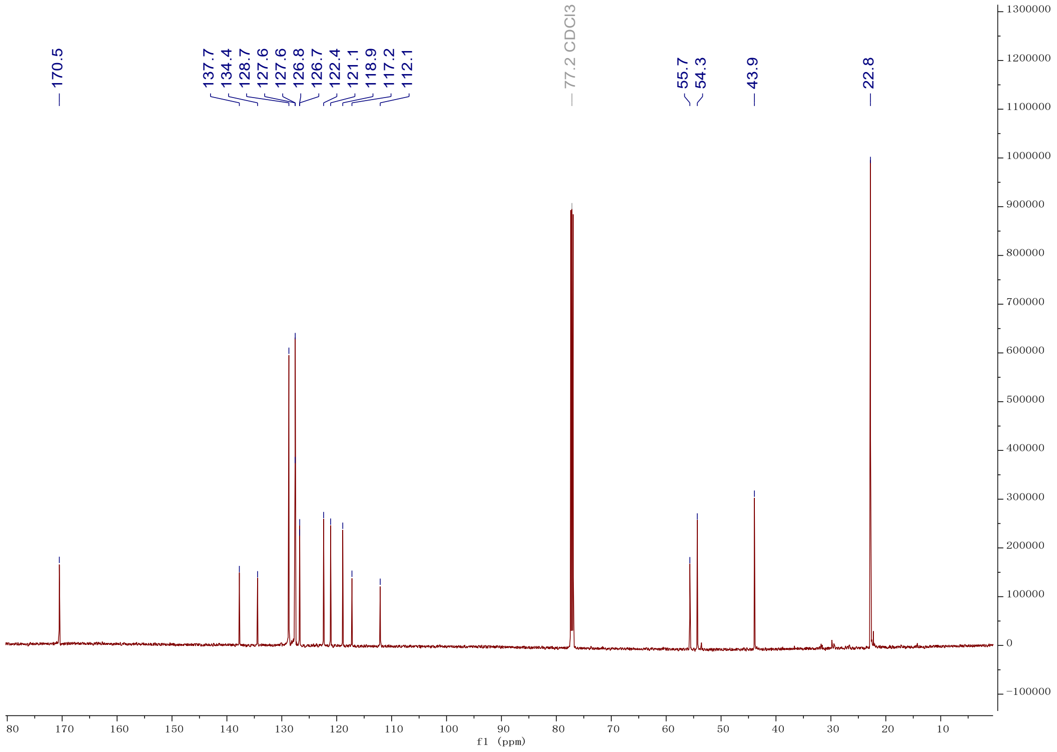
170.5
137.7
134.4
128.7
127.6
127.6
126.8
126.7
122.4
121.1
118.9
117.2
112.1
77.2 CDCl3
55.7
54.3
43.9
22.8
f1 (ppm)

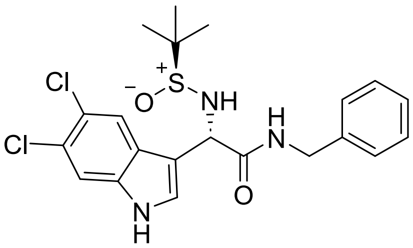
Cl
Cl
O
S
NH
H
N
O
N
H

compound **13**

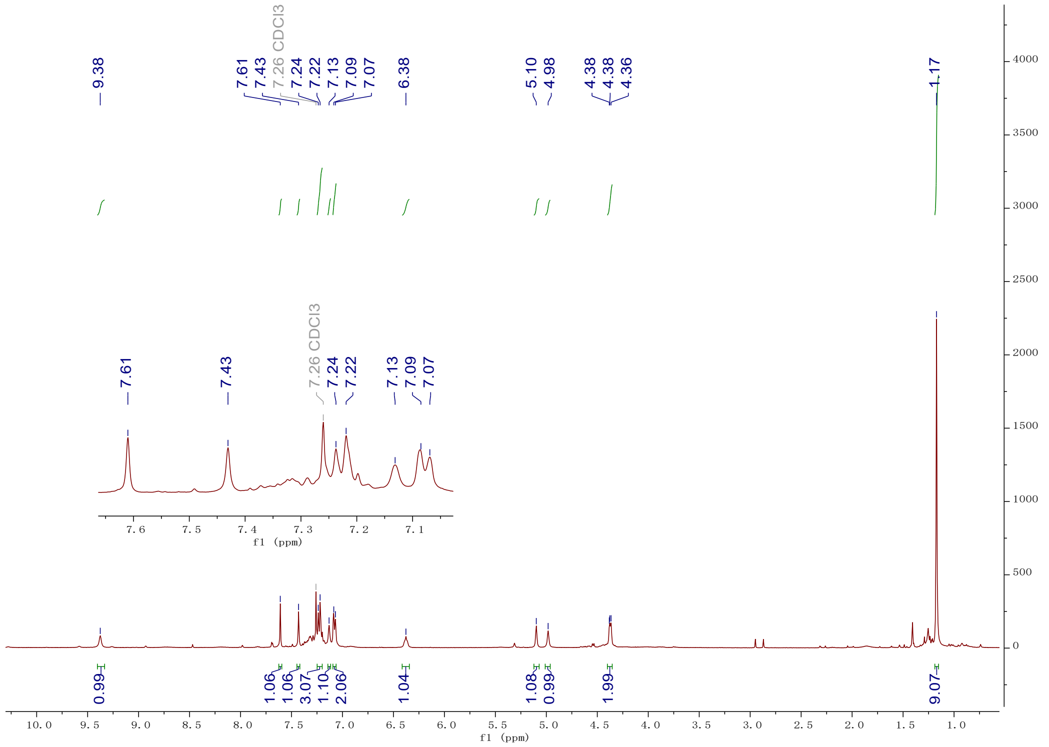
9.38
7.61
7.43
7.26 CDCl3
7.24
7.22
7.13
7.09
7.07
6.38
5.10
4.98
4.38
4.38
4.36
1.17
0.99
1.06
1.06
3.07
1.10
2.06
1.04
1.08
0.99
1.99
9.07
f1 (ppm)

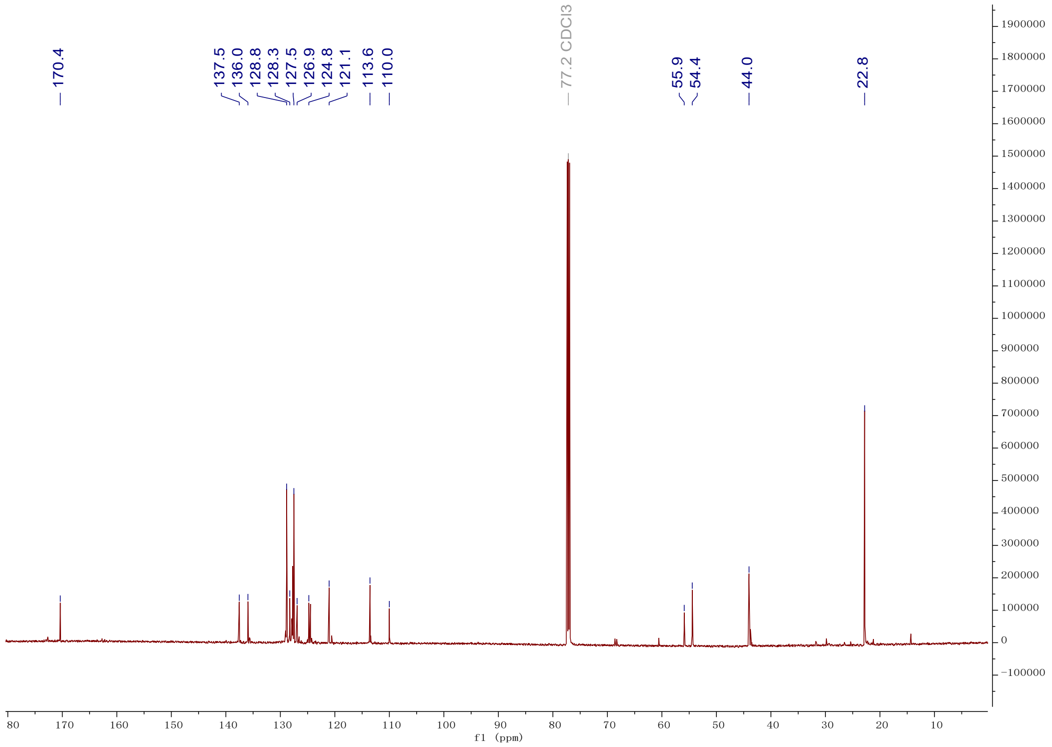
170.4
137.5
136.0
128.8
128.3
127.5
126.9
124.8
121.1
113.6
110.0
77.2 CDCl3
55.9
54.4
44.0
22.8
f1 (ppm)

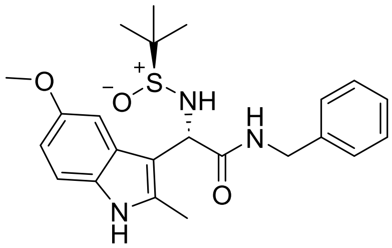

compound **14**

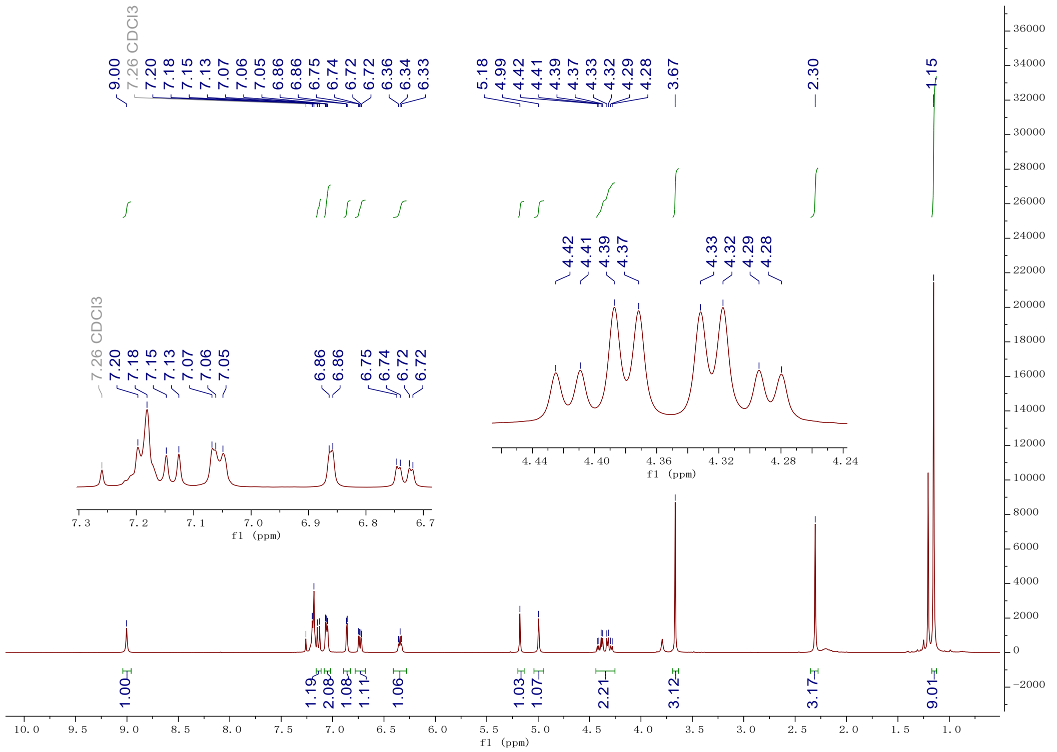
9.00
7.26 CDCl3
7.20
7.18
7.15
7.13
7.07
7.06
7.05
6.86
6.86
6.75
6.74
6.72
6.72
6.36
6.34
6.33
5.18
4.99
4.42
4.41
4.39
4.37
4.33
4.32
4.29
4.28
3.67
2.30
1.15
1.00
1.19
2.08
1.08
1.11
1.06
1.03
1.07
2.21
3.12
3.17
9.01
f1 (ppm)

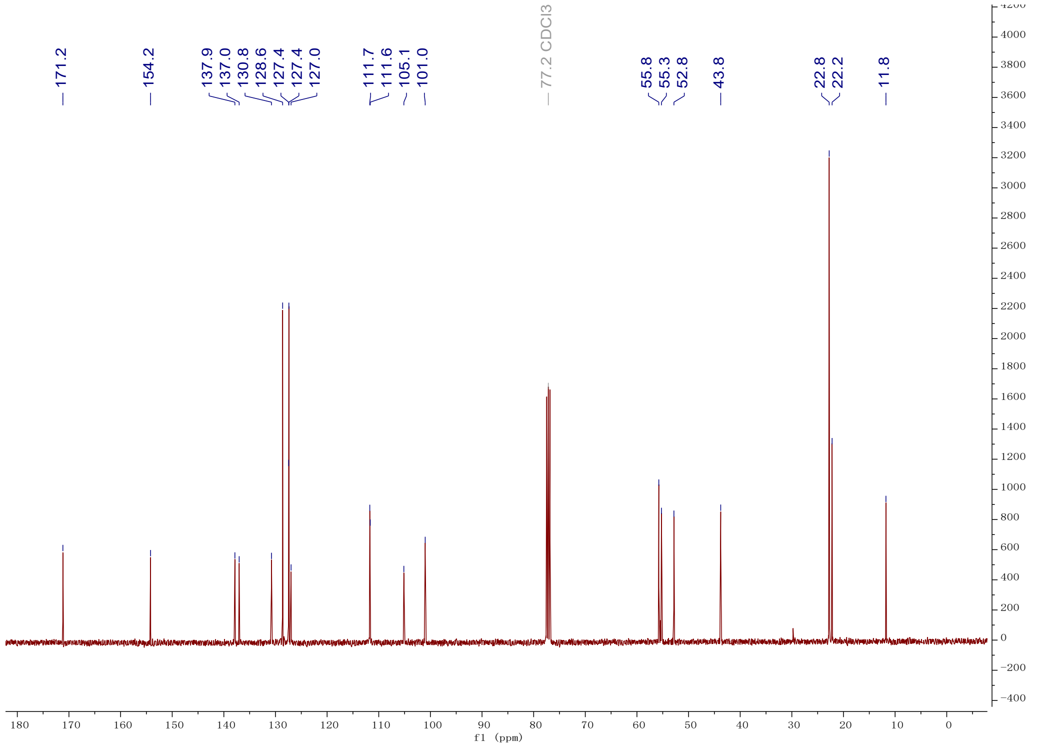
171.2
154.2
137.9
137.0
130.8
128.6
127.4
127.4
127.0
111.7
111.6
105.1
101.0
77.2 CDCl3
55.8
55.3
52.8
43.8
22.8
22.2
11.8
f1 (ppm)

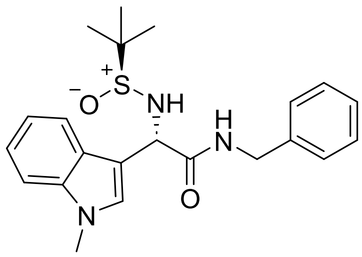
O
S
NH
H
N
O
N

compound **15**

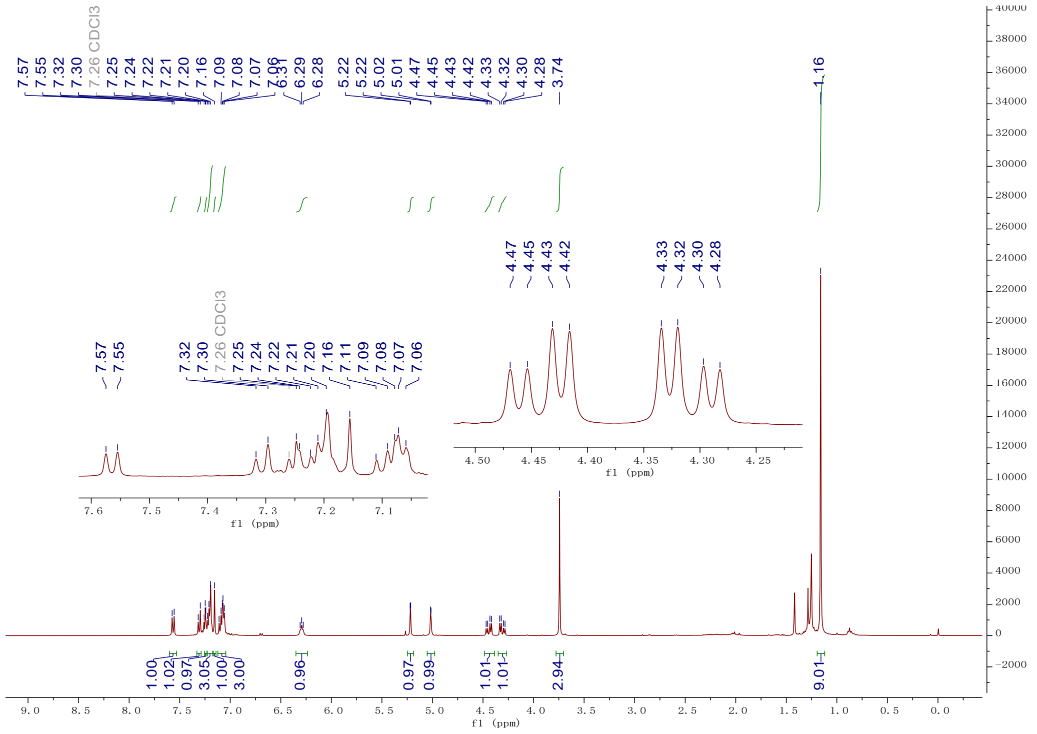
7.57
7.55
7.32
7.30
7.26 CDCl3
7.25
7.24
7.22
7.21
7.20
7.16
7.09
7.08
7.07
7.06
6.31
6.29
6.28
5.22
5.22
5.02
5.01
4.47
4.45
4.43
4.42
4.33
4.32
4.30
4.28
3.74
1.16
1.00
1.02
0.97
3.05
1.00
3.00
0.96
0.97
0.99
1.01
1.01
2.94
9.01
f1 (ppm)

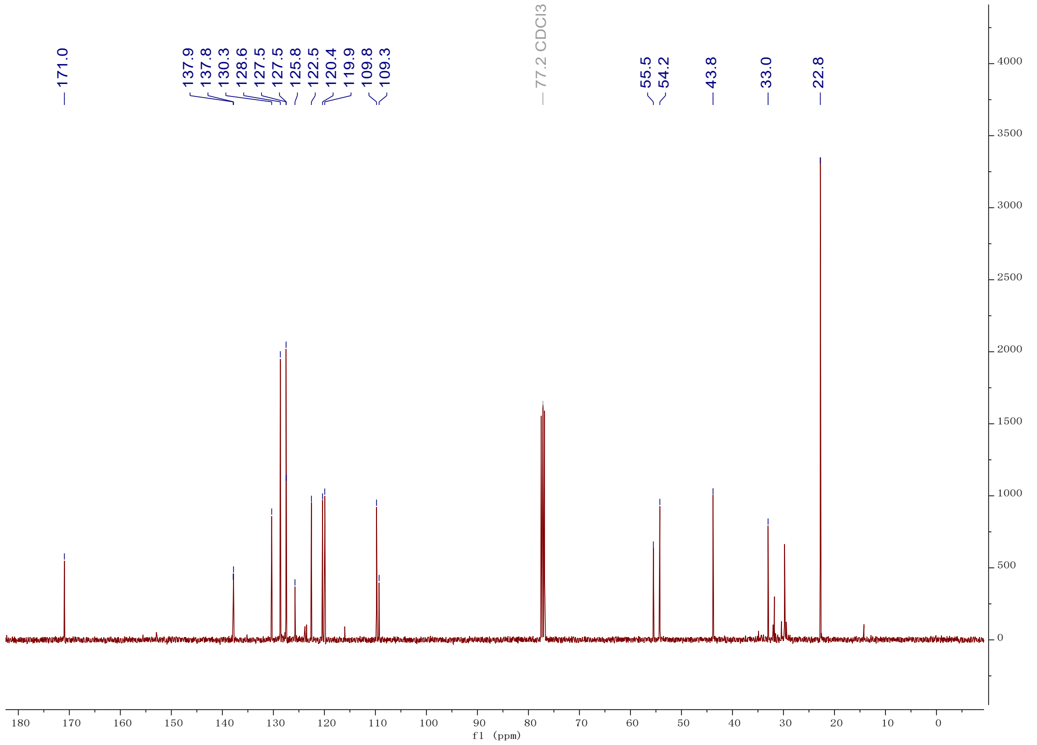
171.0
137.9
137.8
130.3
128.6
127.5
127.5
125.8
122.5
120.4
119.9
109.8
109.3
77.2 CDCl3
55.5
54.2
43.8
33.0
22.8
f1 (ppm)

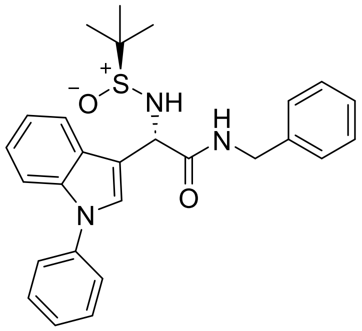

compound **16**

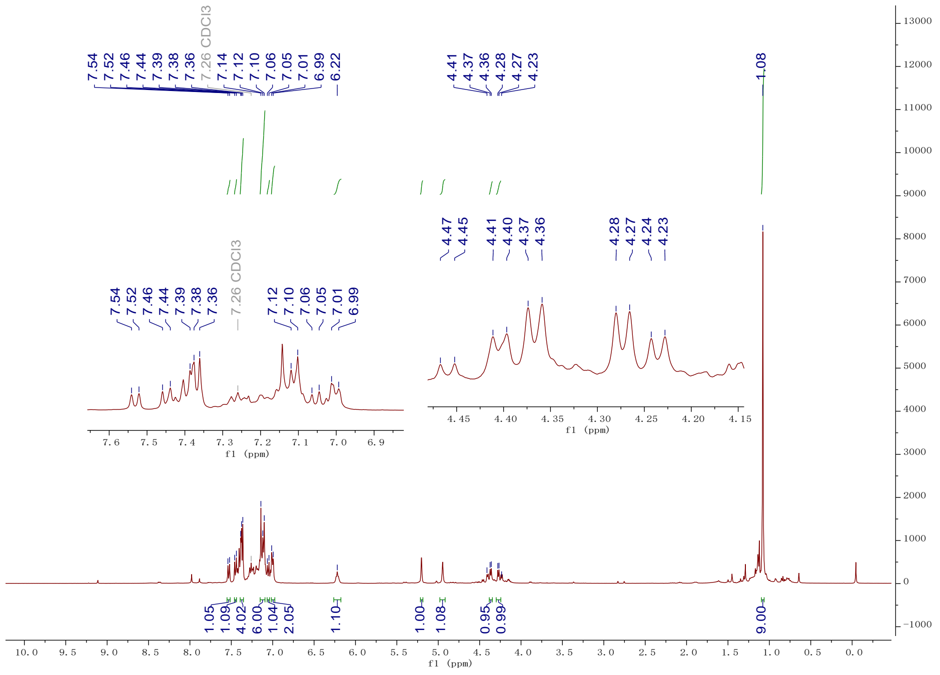

7.54
7.52
7.46
7.44
7.39
7.38
7.36
7.26 CDCl3
7.14
7.12
7.10
7.06
7.05
7.01
6.99
6.22
4.41
4.37
4.36
4.28
4.27
4.23
1.08
4.47
4.45
4.40
4.24
1.05
1.09
4.02
6.00
1.04
2.05
1.10
1.00
1.08
0.95
0.99
9.00
f1 (ppm)

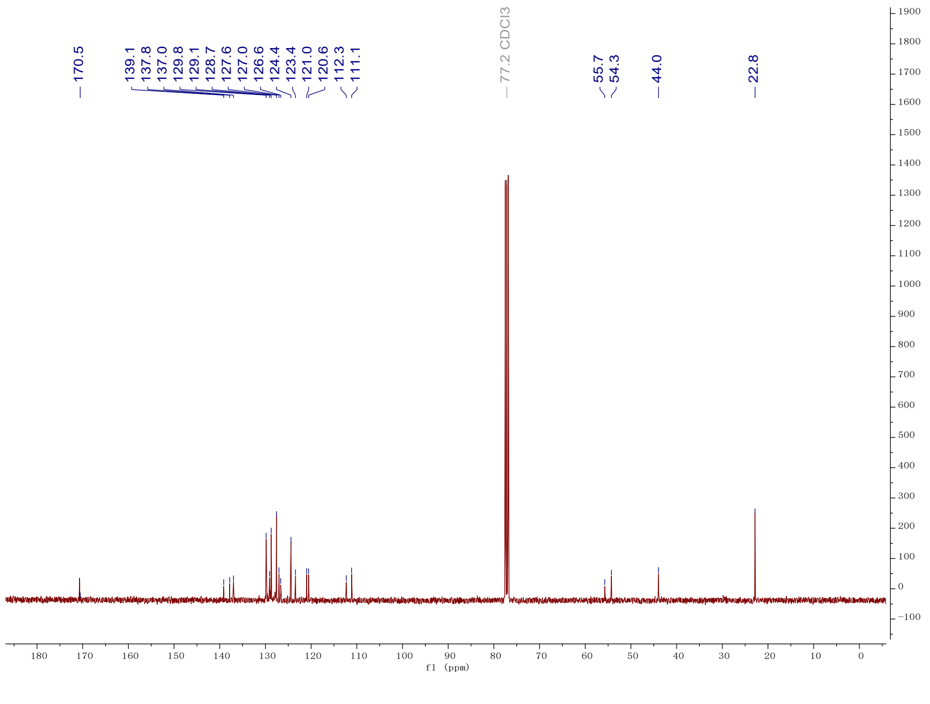

170.5
139.1
137.8
137.0
129.8
129.1
128.7
127.6
127.0
126.6
124.4
123.4
121.0
120.6
112.3
111.1
77.2 CDCl3
55.7
54.3
44.0
22.8
f1 (ppm)

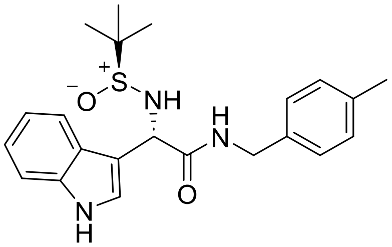
NH
N
H
O
N
H
S
+
−
O

compound **19**

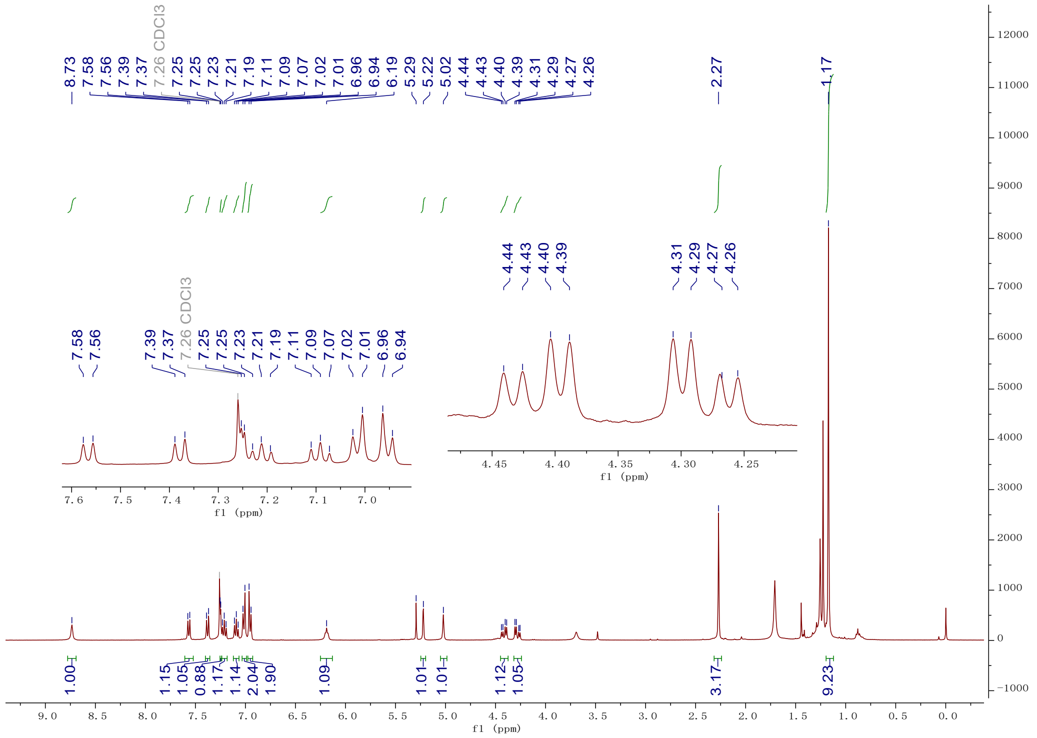
8.73
7.58
7.56
7.39
7.37
7.26 CDCl3
7.25
7.25
7.23
7.21
7.19
7.11
7.09
7.07
7.02
7.01
6.96
6.94
6.19
5.29
5.22
5.02
4.44
4.43
4.40
4.39
4.31
4.29
4.27
4.26
2.27
1.17
1.00
1.15
1.05
0.88
1.17
1.14
2.04
1.90
1.09
1.01
1.01
1.12
1.05
3.17
9.23
f1 (ppm)

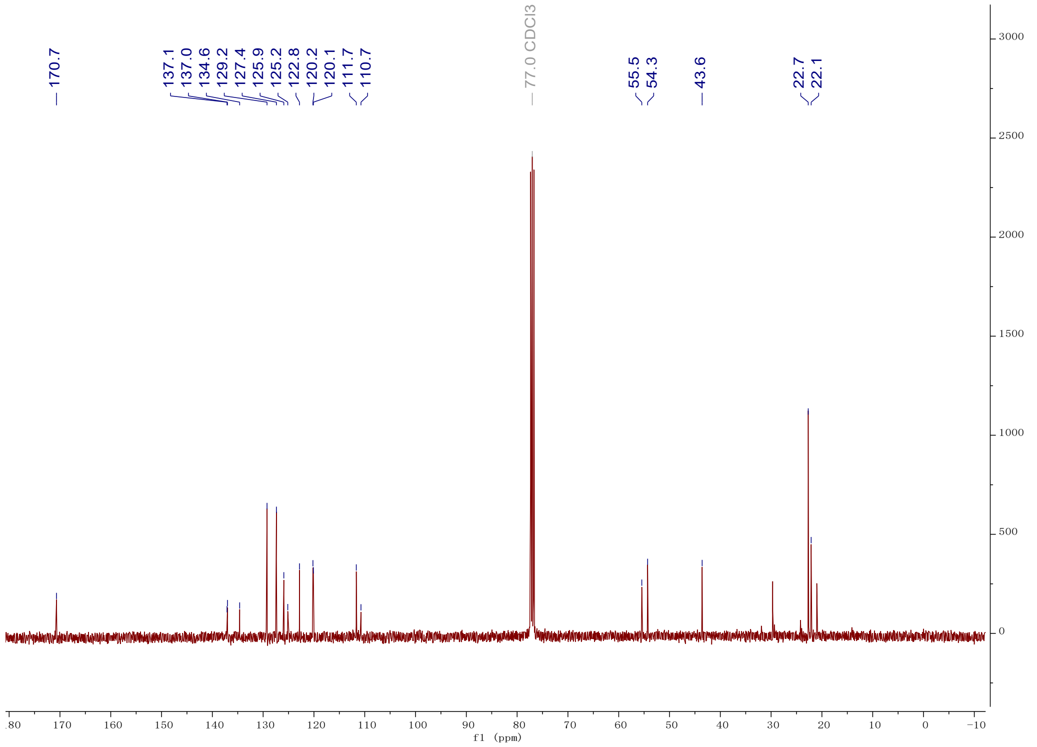
170.7
137.1
137.0
134.6
129.2
127.4
125.9
125.2
122.8
120.2
120.1
111.7
110.7
77.0 CDCl3
55.5
54.3
43.6
22.7
22.1
f1 (ppm)

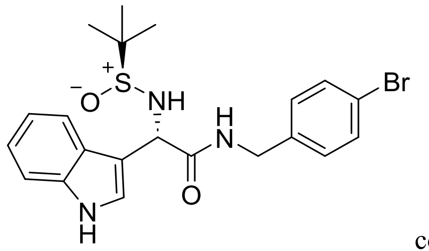

compound **20**

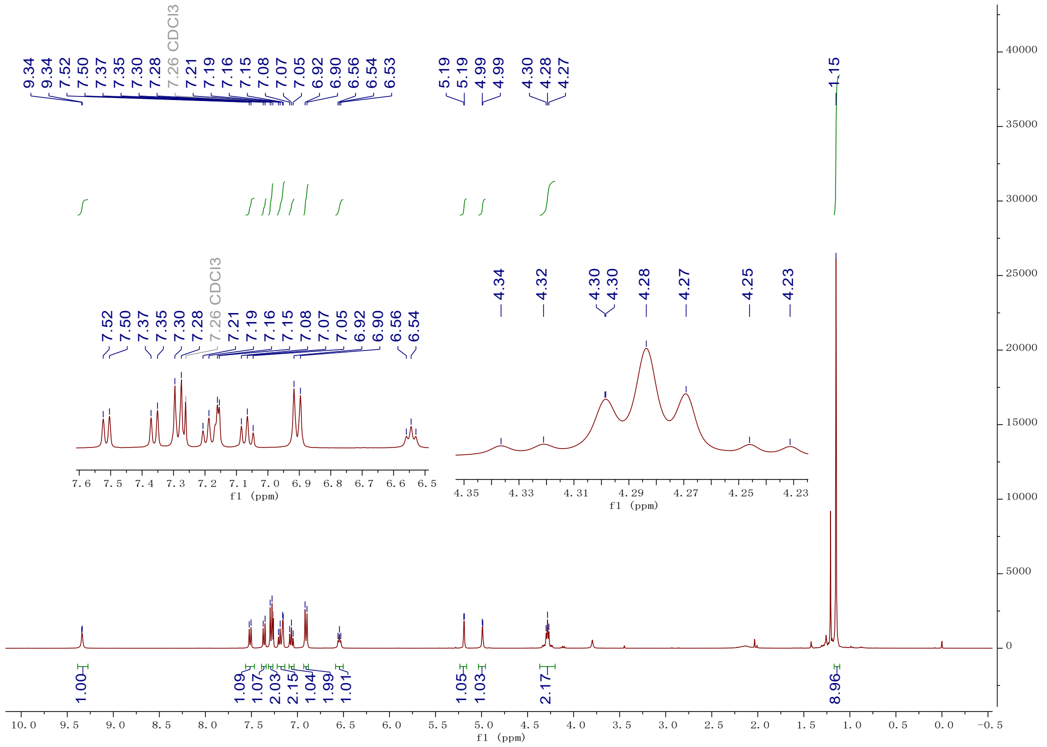

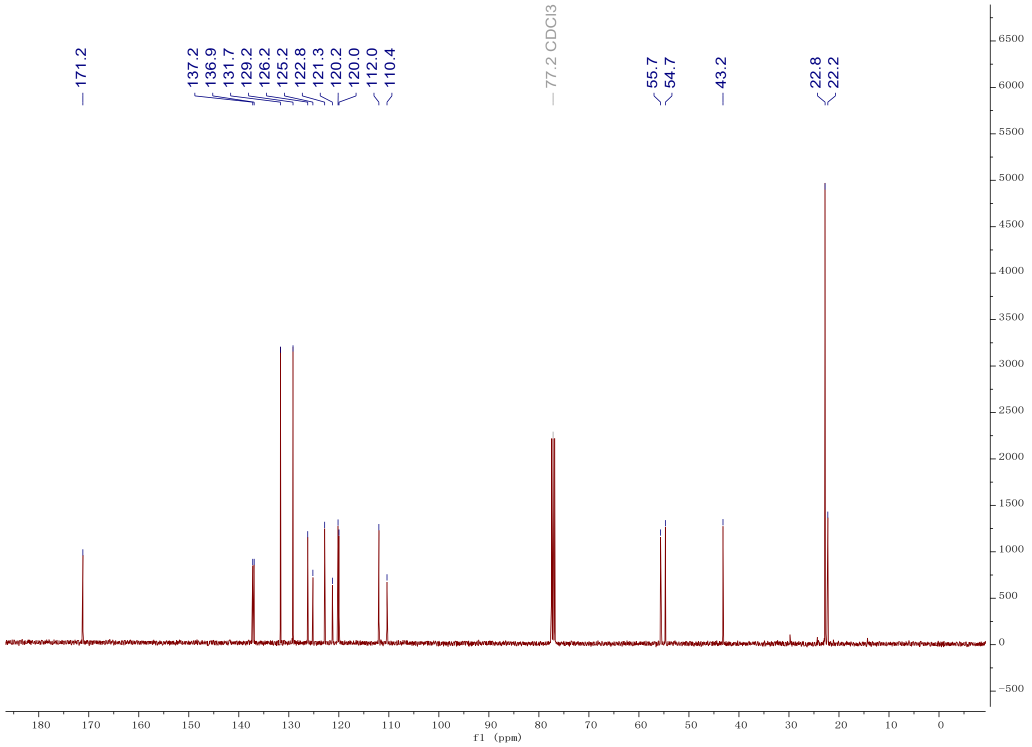

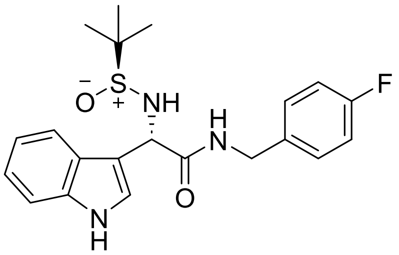
F
NH
H
N
O
N
H

compound **21**

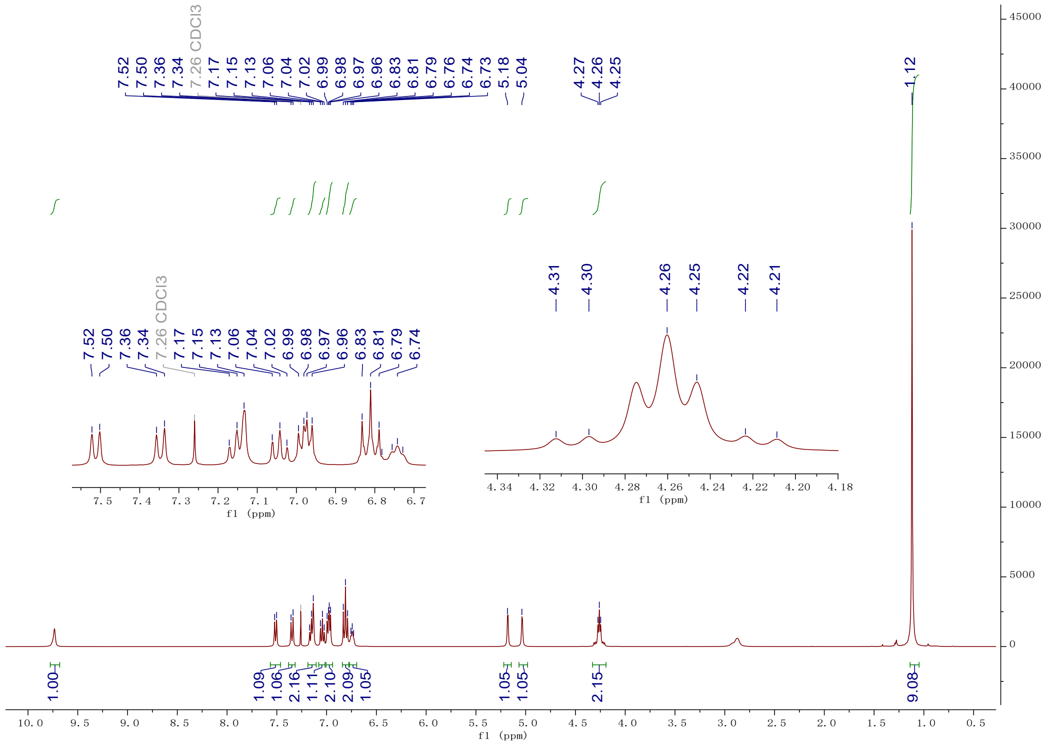
7.52 7.50 7.36 7.34 7.26 CDCl3 7.17 7.15 7.13 7.06 7.04 7.02 6.99 6.98 6.97 6.96 6.83 6.81 6.79 6.76 6.74 6.73 5.18 5.04 4.27 4.26 4.25 1.12
4.31 4.30 4.26 4.25 4.22 4.21
1.00 1.09 1.06 2.16 1.11 2.10 2.09 1.05 1.05 1.05 2.15 9.08
f1 (ppm)

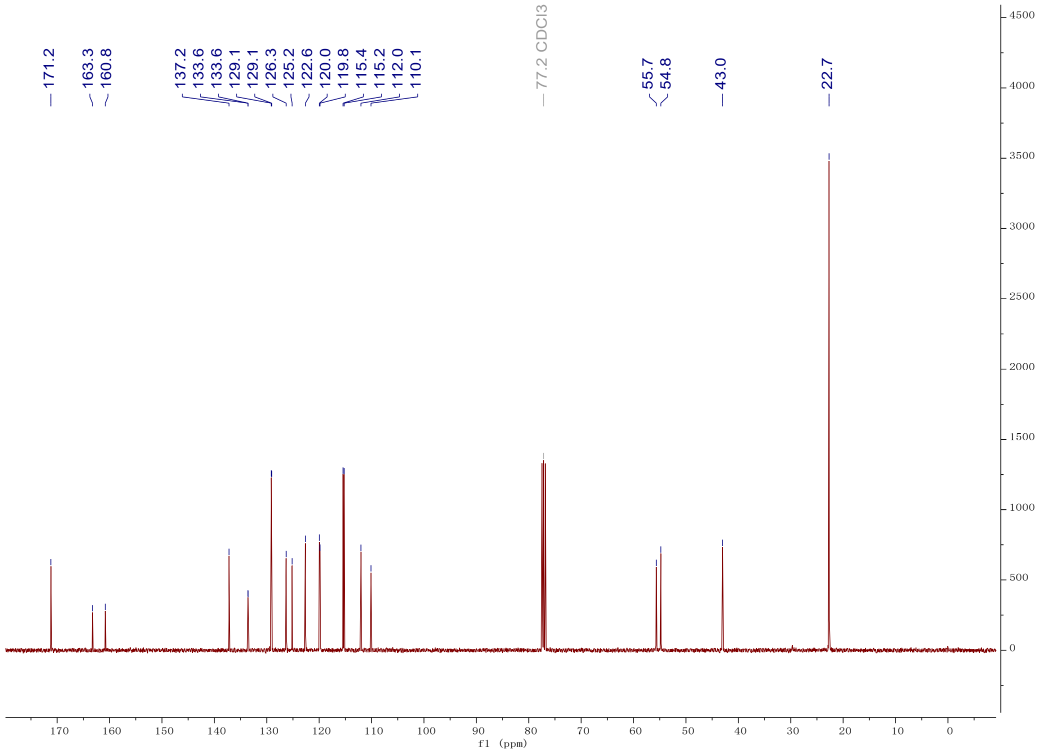
171.2 163.3 160.8 137.2 133.6 129.1 129.1 126.3 125.2 122.6 120.0 119.8 115.4 115.2 112.0 110.1 77.2 CDCl3 55.7 54.8 43.0 22.7
f1 (ppm)

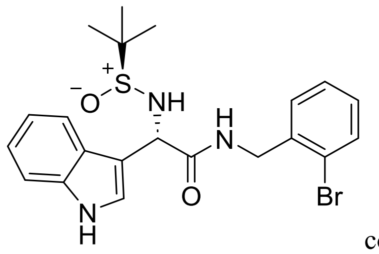
O
S
NH
H
N
N
H
O
Br

compound **22**

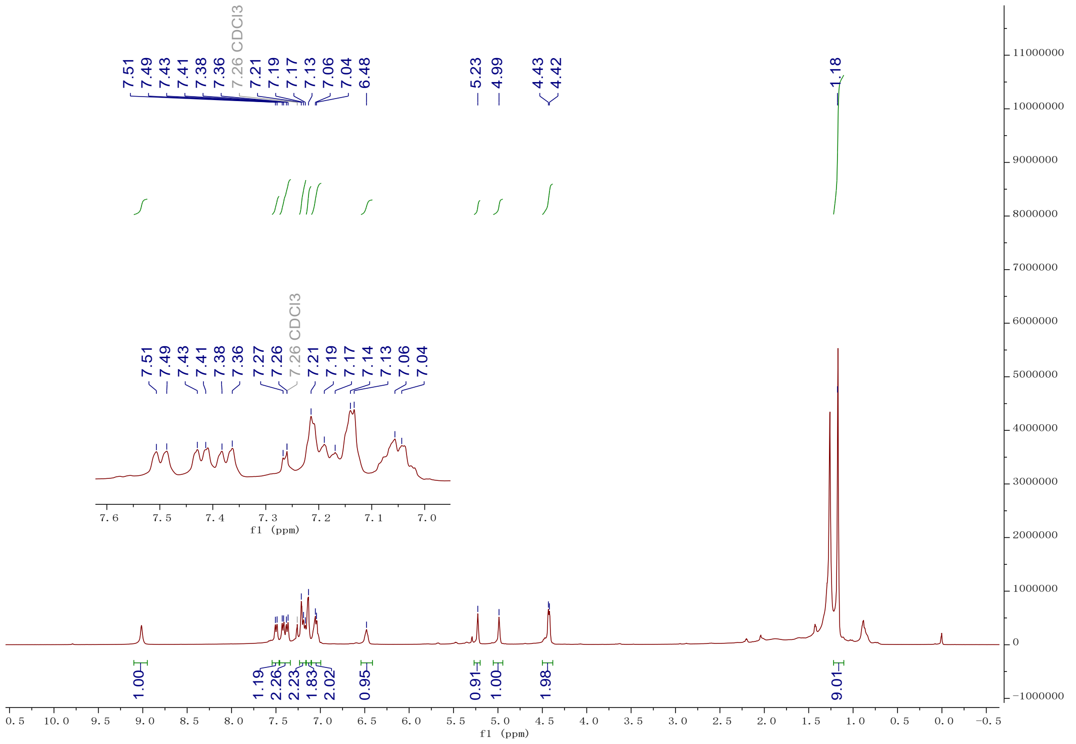
7.51
7.49
7.43
7.41
7.38
7.36
7.26 CDCl3
7.21
7.19
7.17
7.13
7.06
7.04
6.48
5.23
4.99
4.43
4.42
1.18
7.27
7.26
7.14
1.00
1.19
2.26
2.23
1.83
2.02
0.95
0.91
1.00
1.98
9.01
f1 (ppm)

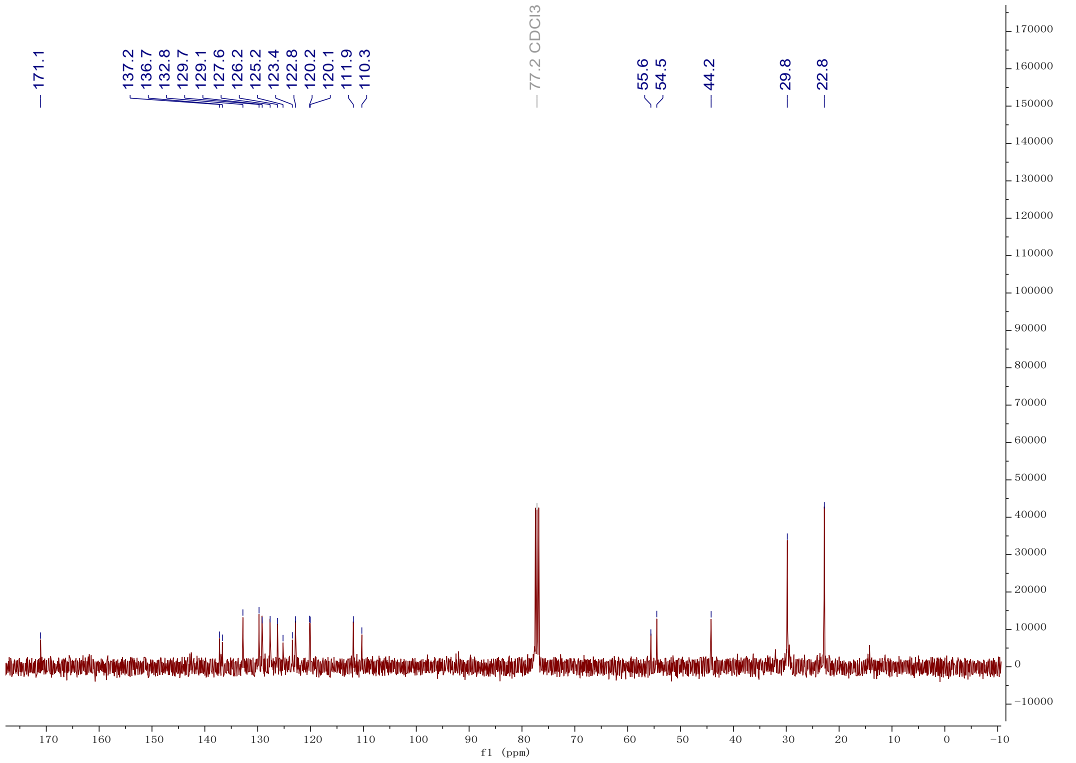
171.1
137.2
136.7
132.8
129.7
129.1
127.6
126.2
125.2
123.4
122.8
120.2
120.1
111.9
110.3
77.2 CDCl3
55.6
54.5
44.2
29.8
22.8
f1 (ppm)

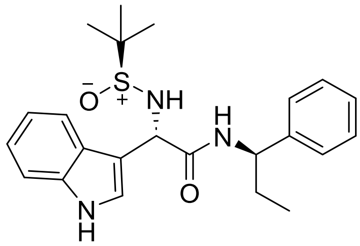

compound **23**

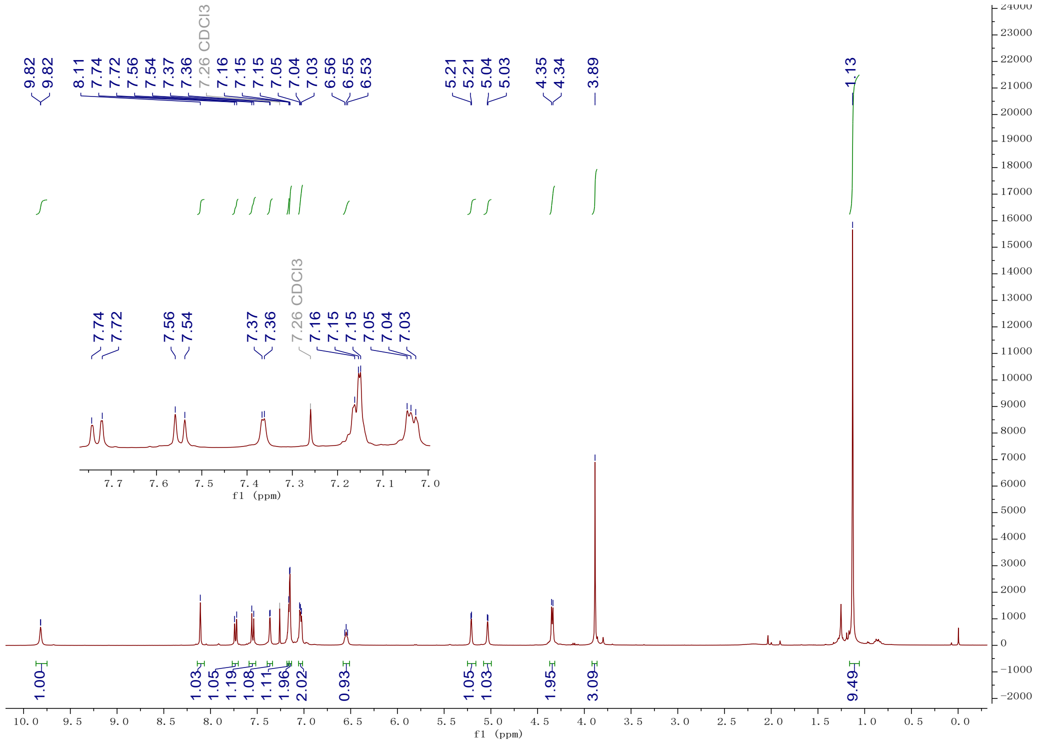
9.82
9.82
8.11
7.74
7.72
7.56
7.54
7.37
7.36
7.26 CDCl3
7.16
7.15
7.15
7.05
7.04
7.03
6.56
6.55
6.53
5.21
5.21
5.04
5.03
4.35
4.34
3.89
1.13
1.00
1.03
1.05
1.19
1.08
1.11
1.96
2.02
0.93
1.05
1.03
1.95
3.09
9.49
f1 (ppm)

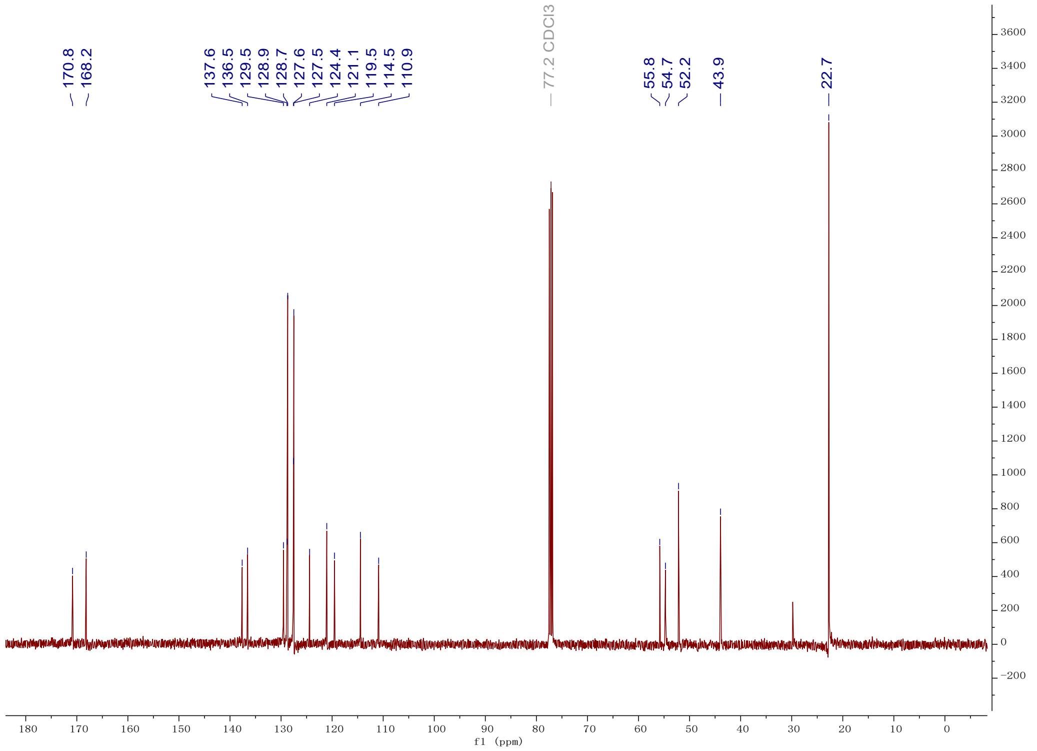
170.8
168.2
137.6
136.5
129.5
128.9
128.7
127.6
127.5
124.4
121.1
119.5
114.5
110.9
77.2 CDCl3
55.8
54.7
52.2
43.9
22.7
f1 (ppm)

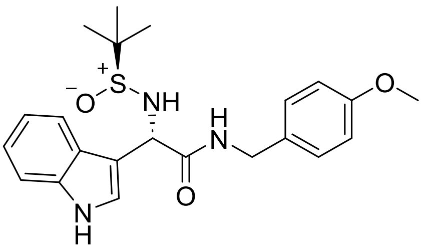

compound **24**

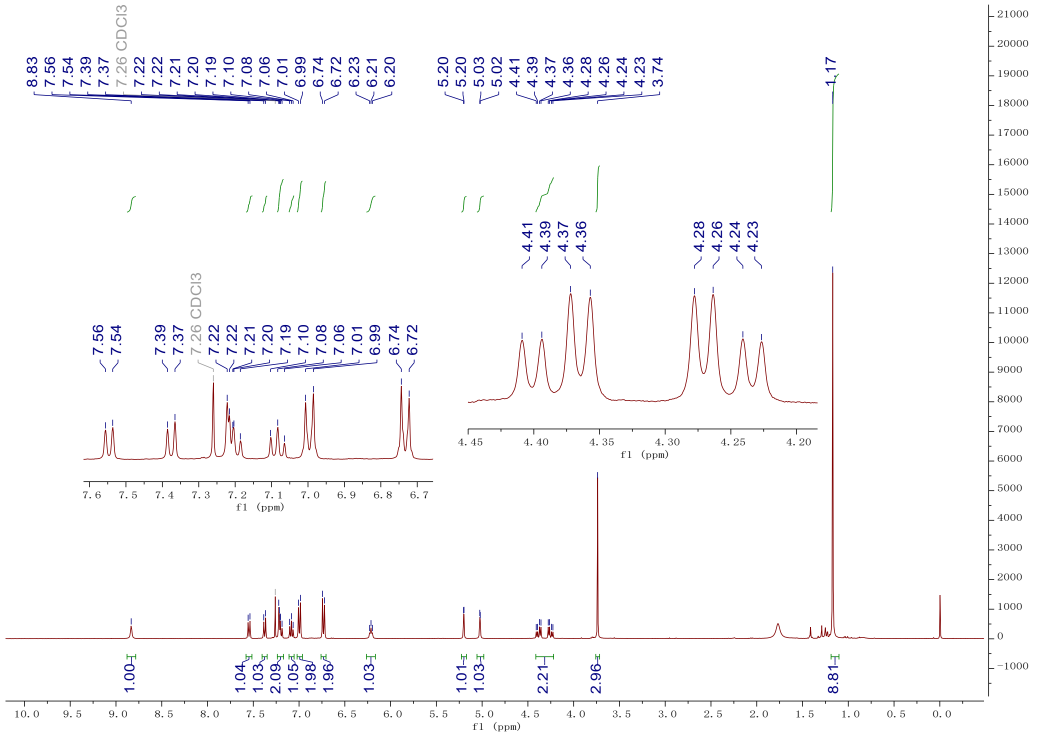
8.83 7.56 7.54 7.39 7.37 7.26 CDCl3 7.22 7.22 7.21 7.20 7.19 7.10 7.08 7.06 7.01 6.99 6.74 6.72 6.23 6.21 6.20 5.20 5.20 5.03 5.02 4.41 4.39 4.37 4.36 4.28 4.26 4.24 4.23 3.74 1.17
1.00 1.04 1.03 2.09 1.05 1.98 1.96 1.03 1.01 1.03 2.21 2.96 8.81
f1 (ppm)

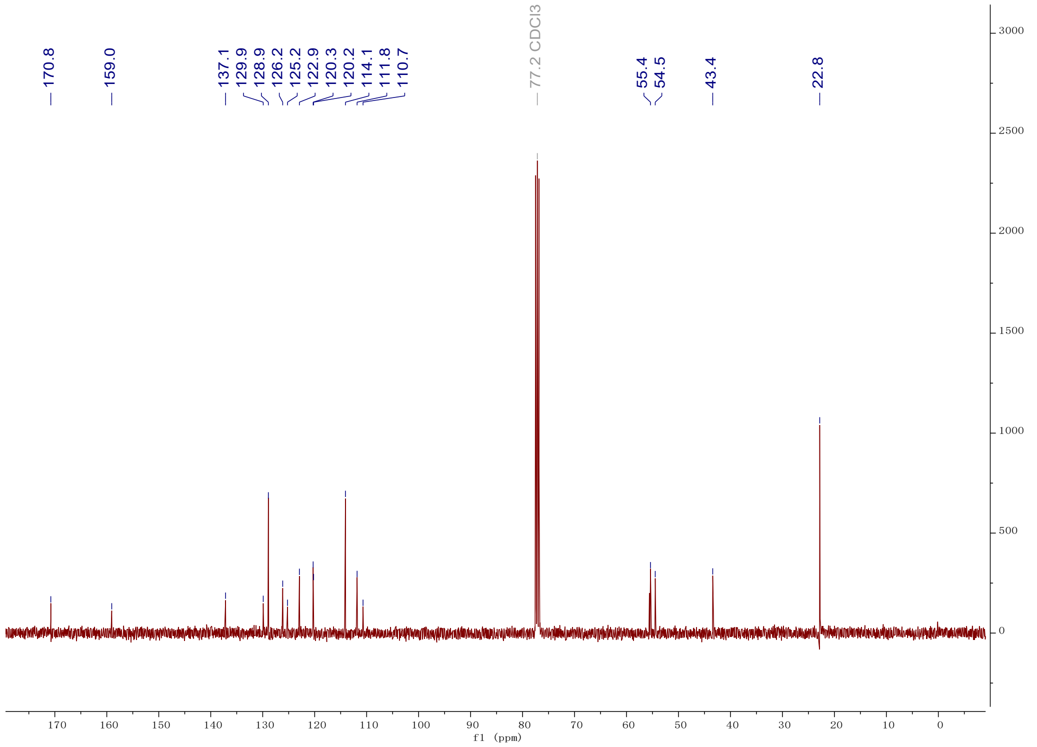
170.8 159.0 137.1 129.9 128.9 126.2 125.2 122.9 120.3 120.2 114.1 111.8 110.7 77.2 CDCl3 55.4 54.5 43.4 22.8
f1 (ppm)

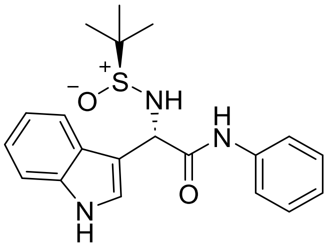
NH
H
N
O
NH
H
S
O

compound **25**

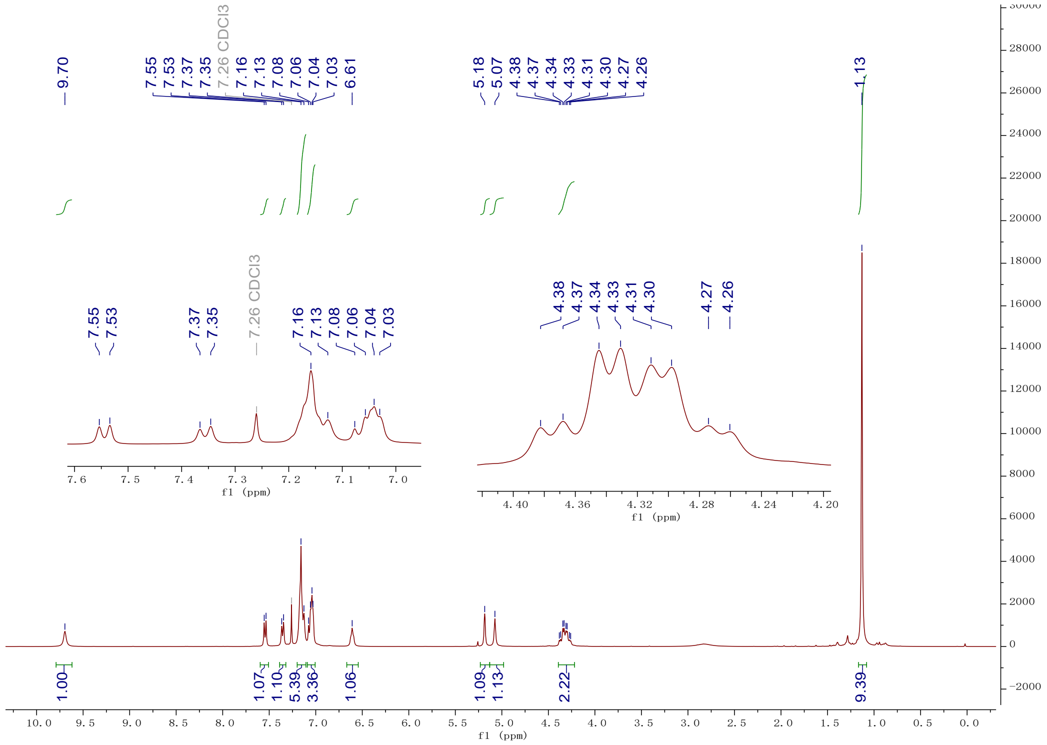
9.70
7.55
7.53
7.37
7.35
7.26 CDCl3
7.16
7.13
7.08
7.06
7.04
7.03
6.61
5.18
5.07
4.38
4.37
4.34
4.33
4.31
4.30
4.27
4.26
1.13
1.00
1.07
1.10
5.39
3.36
1.06
1.09
1.13
2.22
9.39
f1 (ppm)

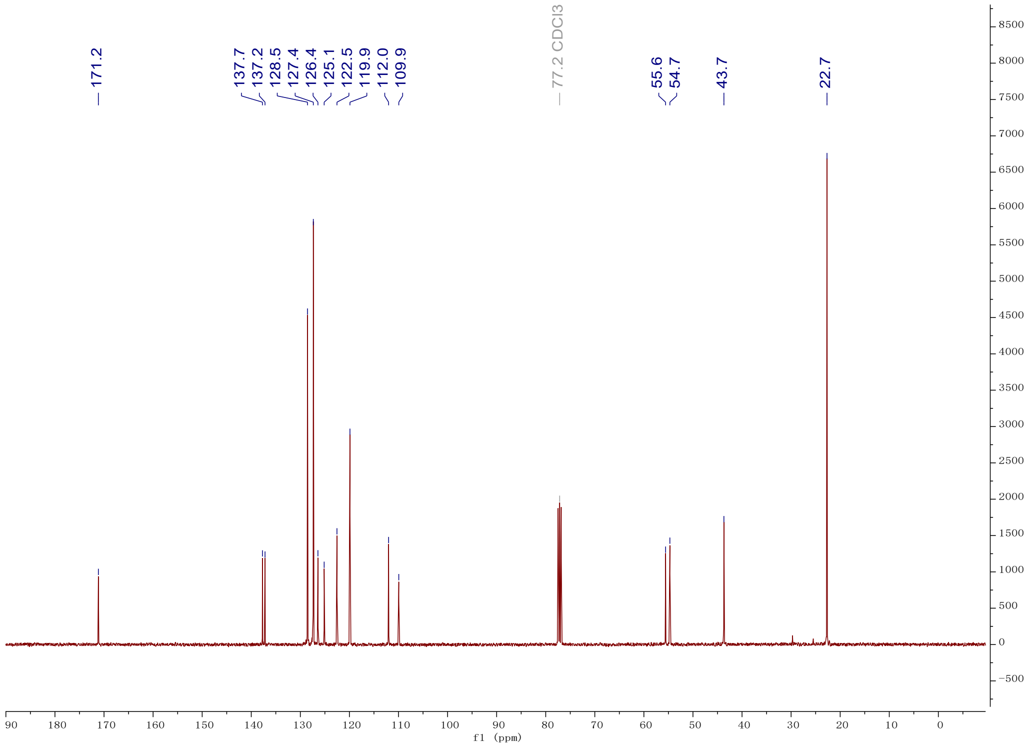
171.2
137.7
137.2
128.5
127.4
126.4
125.1
122.5
119.9
112.0
109.9
77.2 CDCl3
55.6
54.7
43.7
22.7
f1 (ppm)

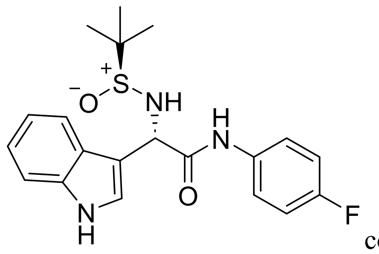
NH
H
N
O
F
N
H
O
S
+
−

compound **26**

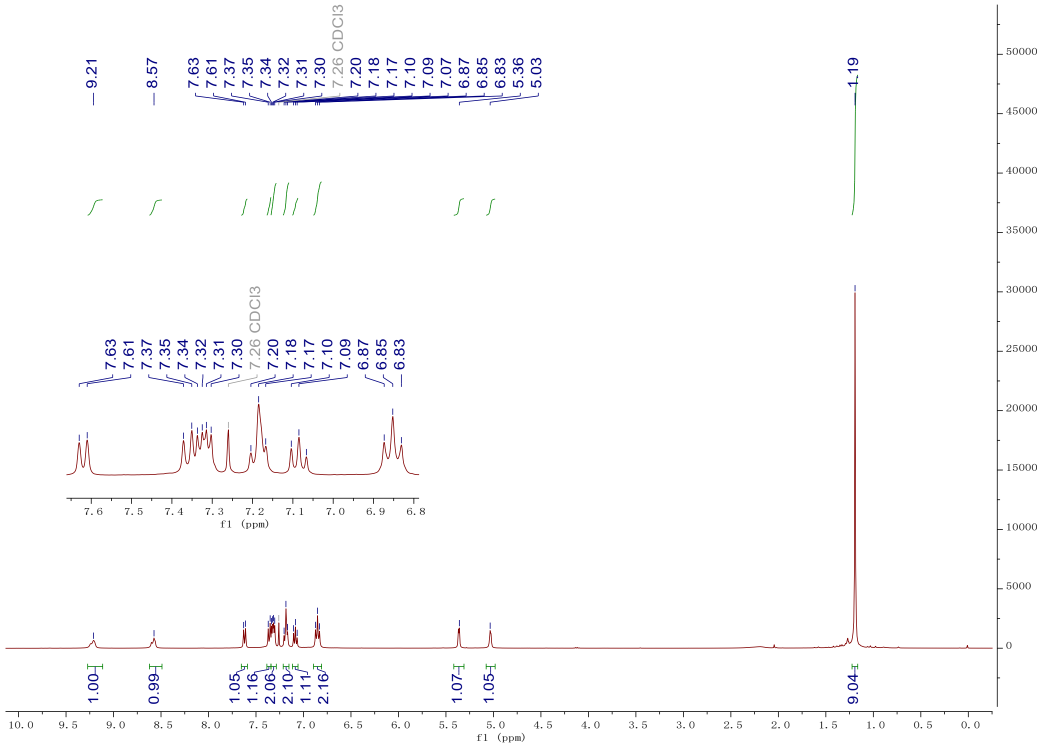
9.21
8.57
7.63
7.61
7.37
7.35
7.34
7.32
7.31
7.30
7.26 CDCl3
7.20
7.18
7.17
7.10
7.09
7.07
6.87
6.85
6.83
5.36
5.03
1.19
1.00
0.99
1.05
1.16
2.06
2.10
1.11
2.16
1.07
1.05
9.04
f1 (ppm)

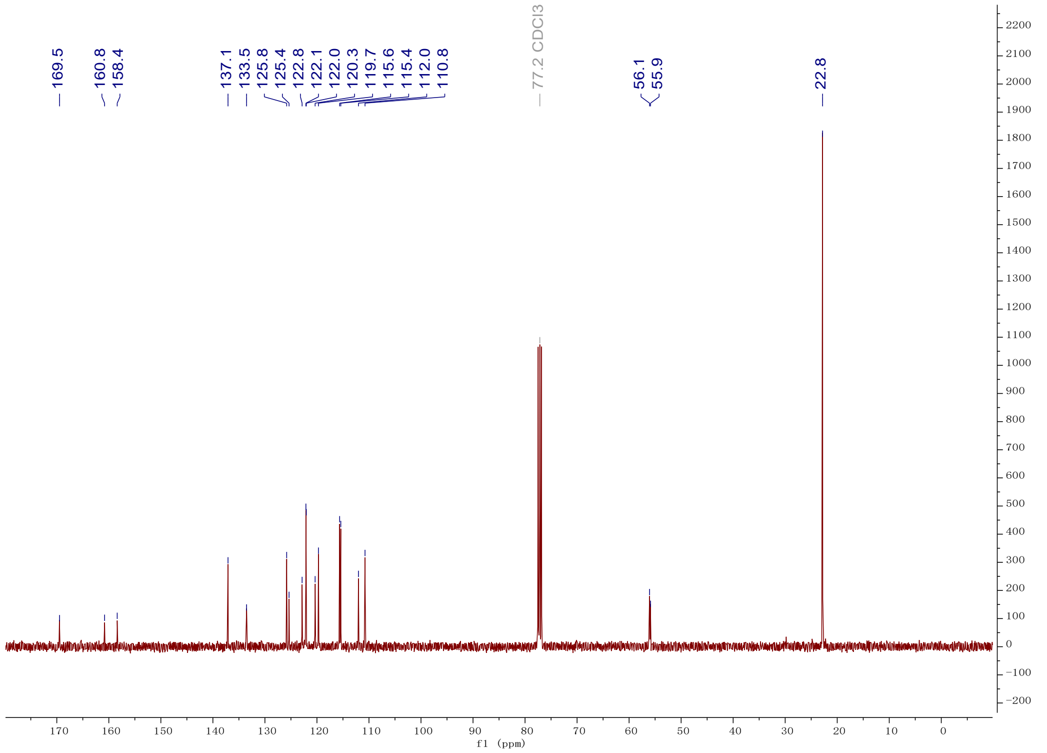
169.5
160.8
158.4
137.1
133.5
125.8
125.4
122.8
122.1
122.0
120.3
119.7
115.6
115.4
112.0
110.8
77.2 CDCl3
56.1
55.9
22.8
f1 (ppm)

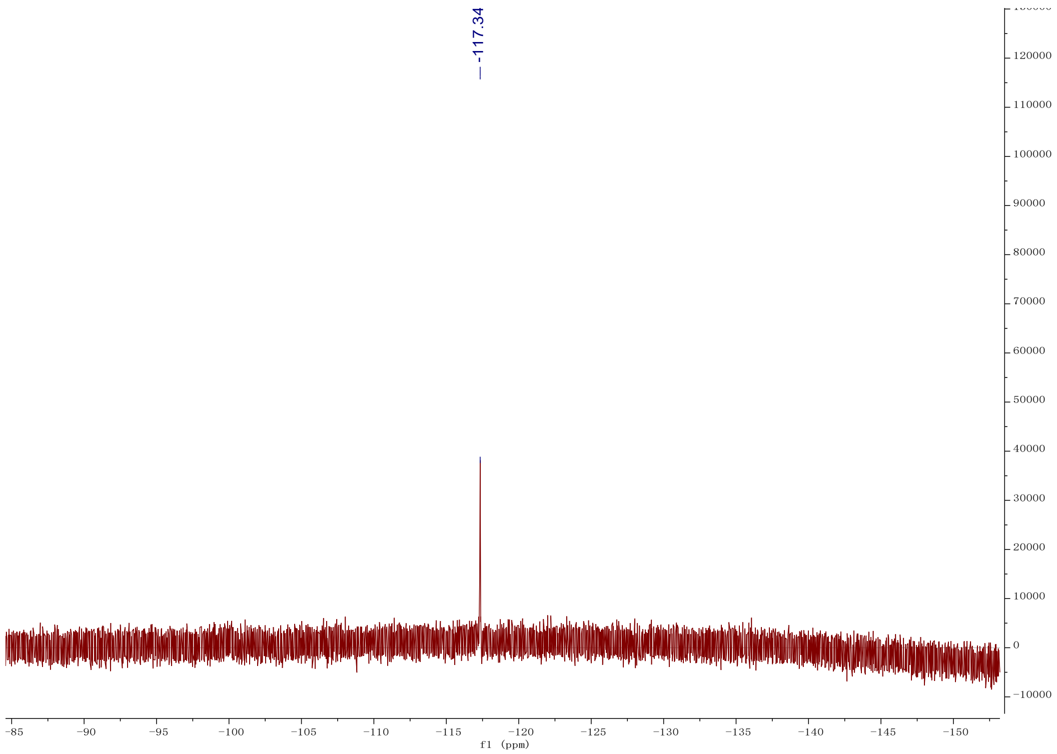
-117.34
120000
110000
100000
90000
80000
70000
60000
50000
40000
30000
20000
10000
0
-10000
-85
-90
-95
-100
-105
-110
-115
-120
-125
-130
-135
-140
-145
-150
f1 (ppm)

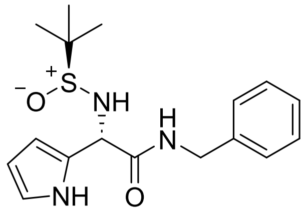

compound **27**

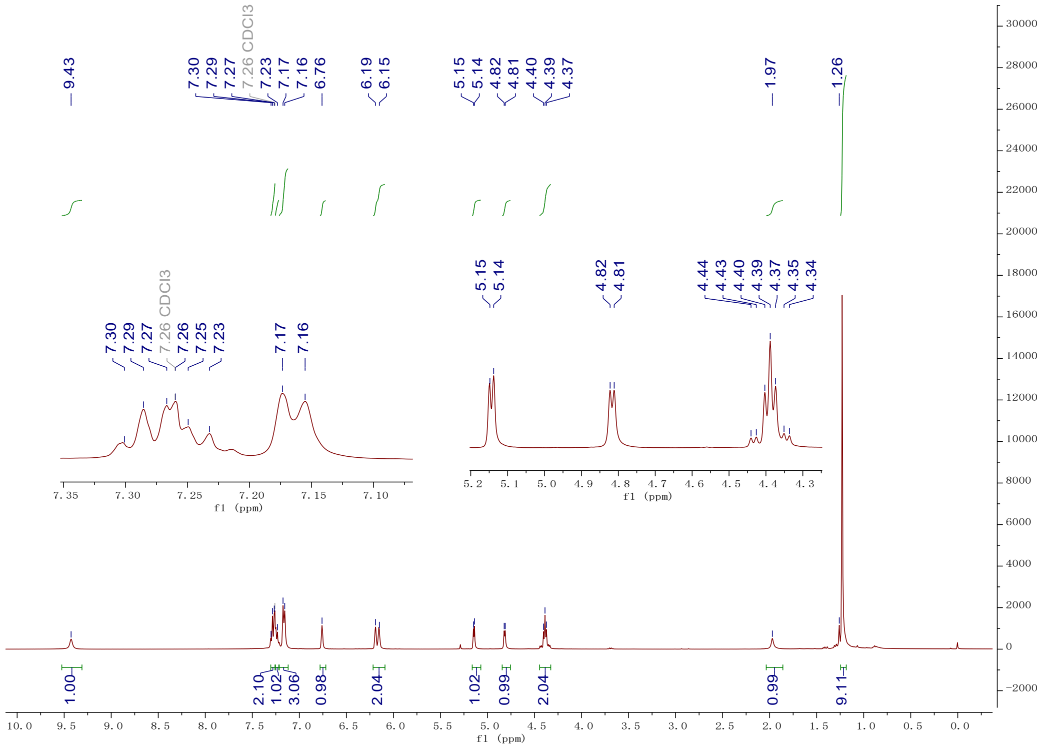

9.43
7.30
7.29
7.27
7.26 CDCl3
7.23
7.17
7.16
6.76
6.19
6.15
5.15
5.14
4.82
4.81
4.40
4.39
4.37
1.97
1.26
1.00
2.10
1.02
3.06
0.98
2.04
1.02
0.99
2.04
0.99
9.11
f1 (ppm)

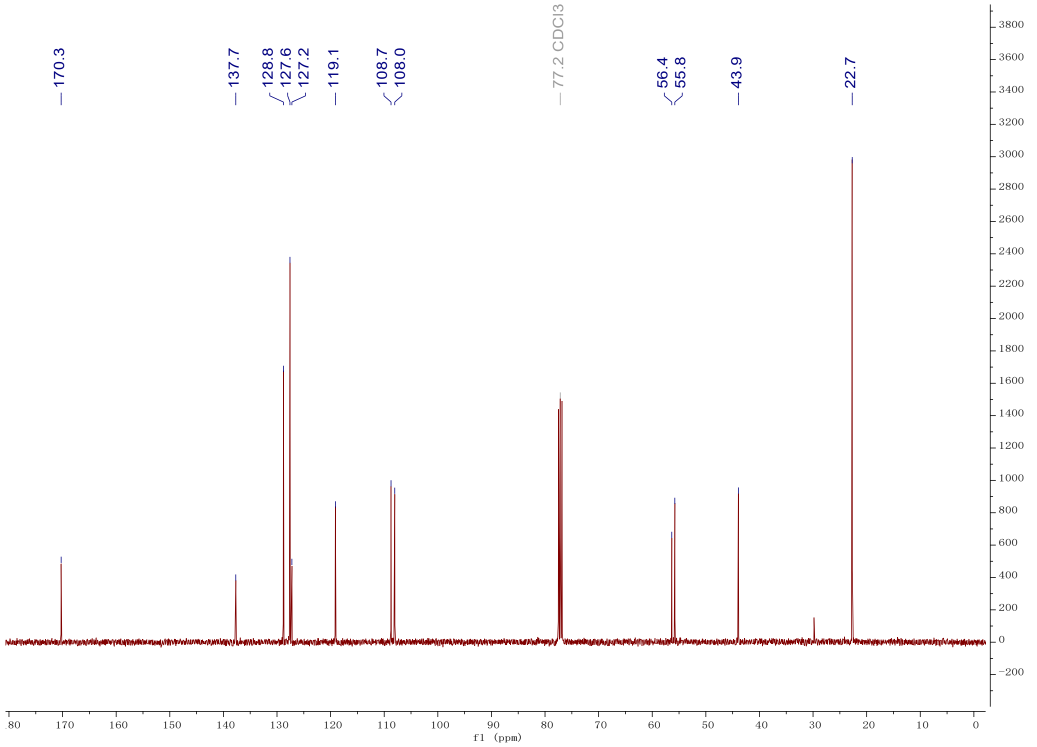

170.3
137.7
128.8
127.6
127.2
119.1
108.7
108.0
77.2 CDCl3
56.4
55.8
43.9
22.7
f1 (ppm)

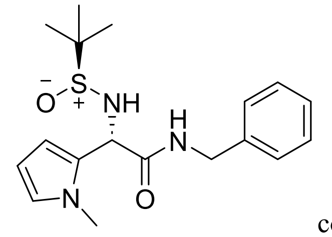
S
NH
O
H
N
N
O

compound **28**

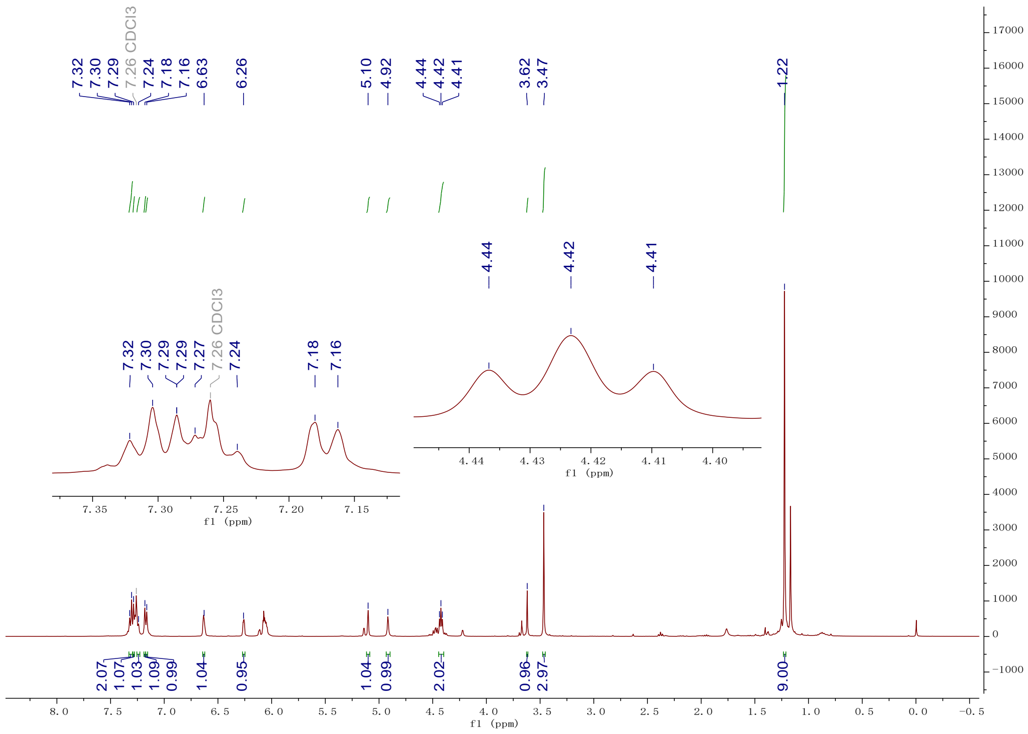
7.32
7.30
7.29
7.26 CDCl3
7.24
7.18
7.16
6.63
6.26
5.10
4.92
4.44
4.42
4.41
3.62
3.47
1.22
2.07
1.07
1.03
1.09
0.99
1.04
0.95
1.04
0.99
2.02
0.96
2.97
9.00
f1 (ppm)

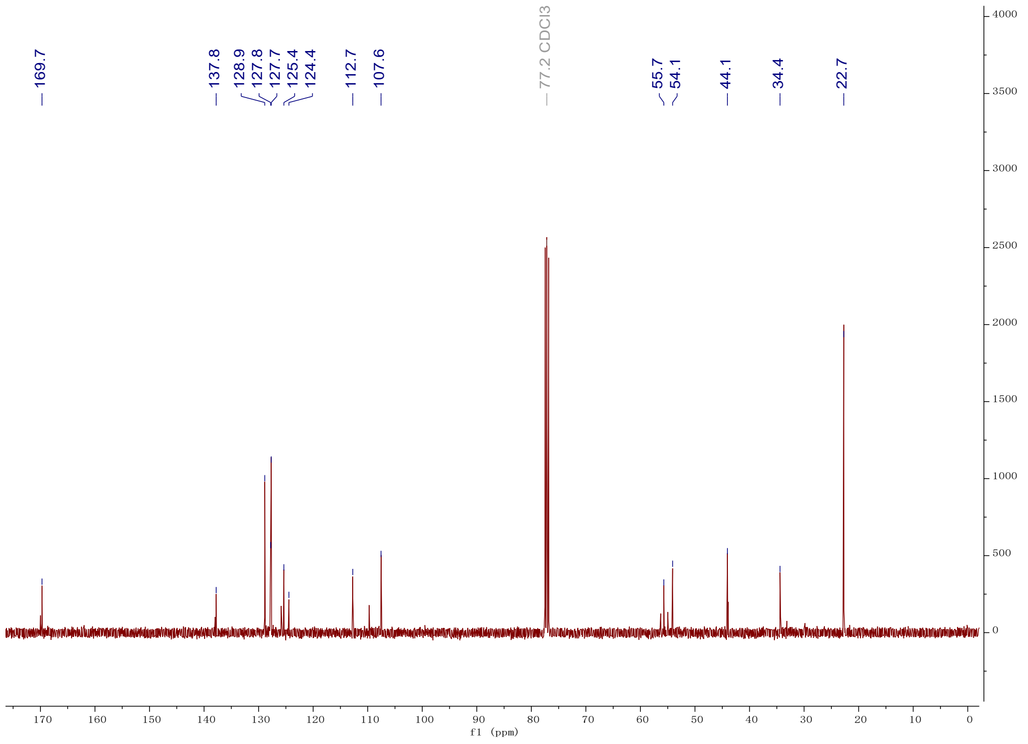
169.7
137.8
128.9
127.8
127.7
125.4
124.4
112.7
107.6
77.2 CDCl3
55.7
54.1
44.1
34.4
22.7
f1 (ppm)

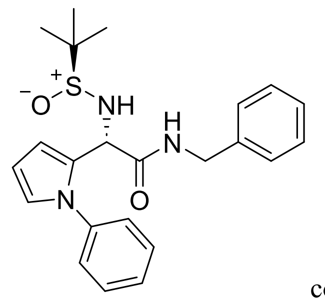
O
S
NH
H
N
N
O

compound **29**

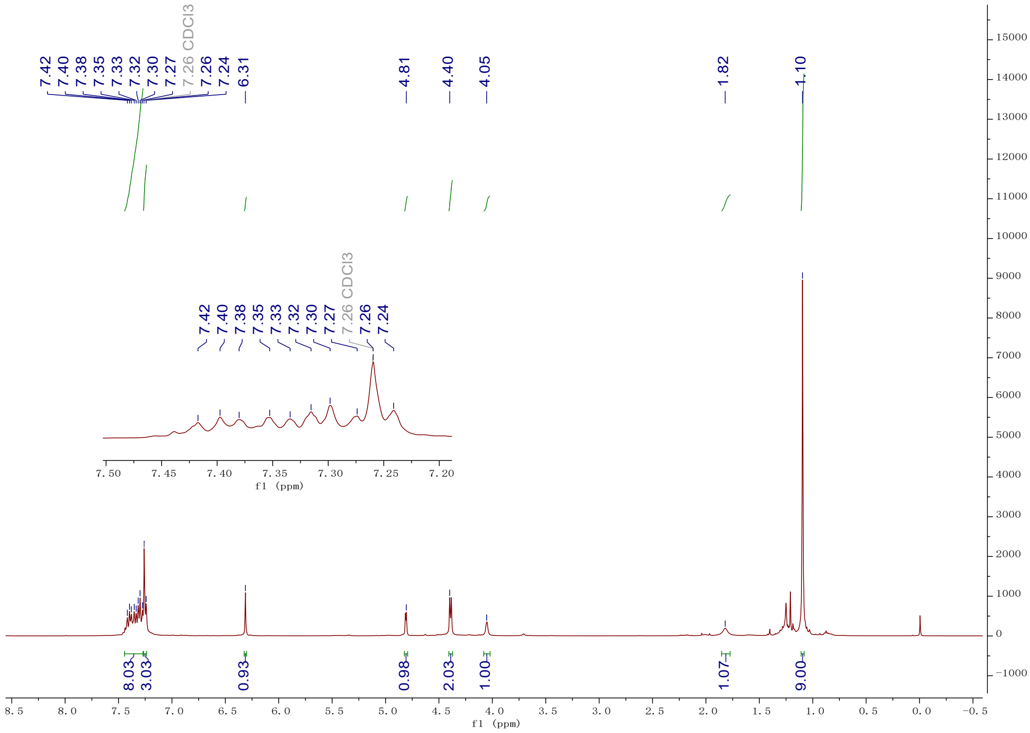
7.42
7.40
7.38
7.35
7.33
7.32
7.30
7.27
7.26 CDCl3
7.26
7.24
6.31
4.81
4.40
4.05
1.82
1.10
8.03
3.03
0.93
0.98
2.03
1.00
1.07
9.00
f1 (ppm)

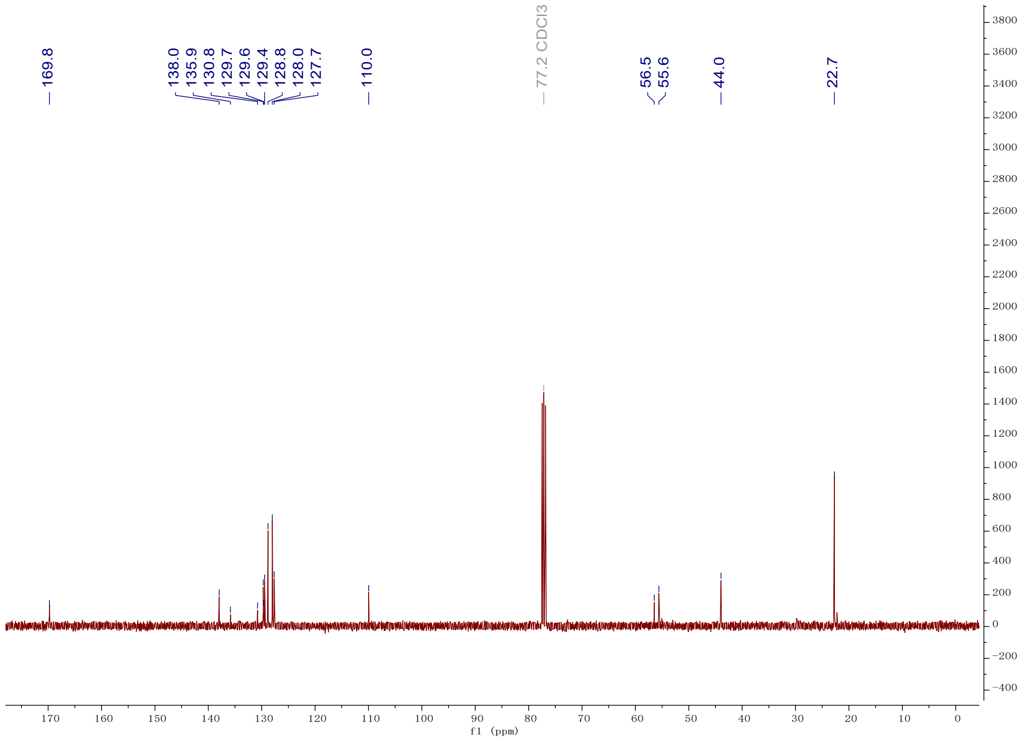
169.8
138.0
135.9
130.8
129.7
129.6
129.4
128.8
128.0
127.7
110.0
77.2 CDCl3
56.5
55.6
44.0
22.7
f1 (ppm)

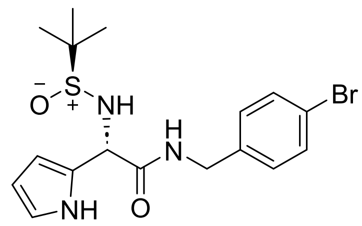

compound **31**

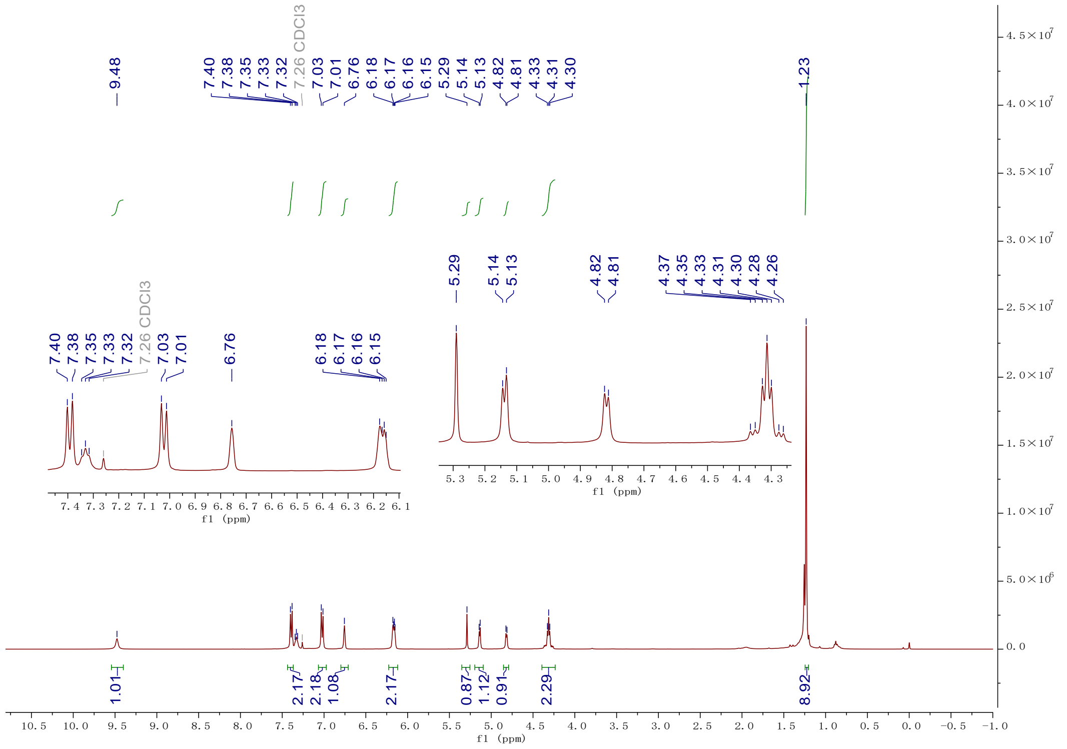

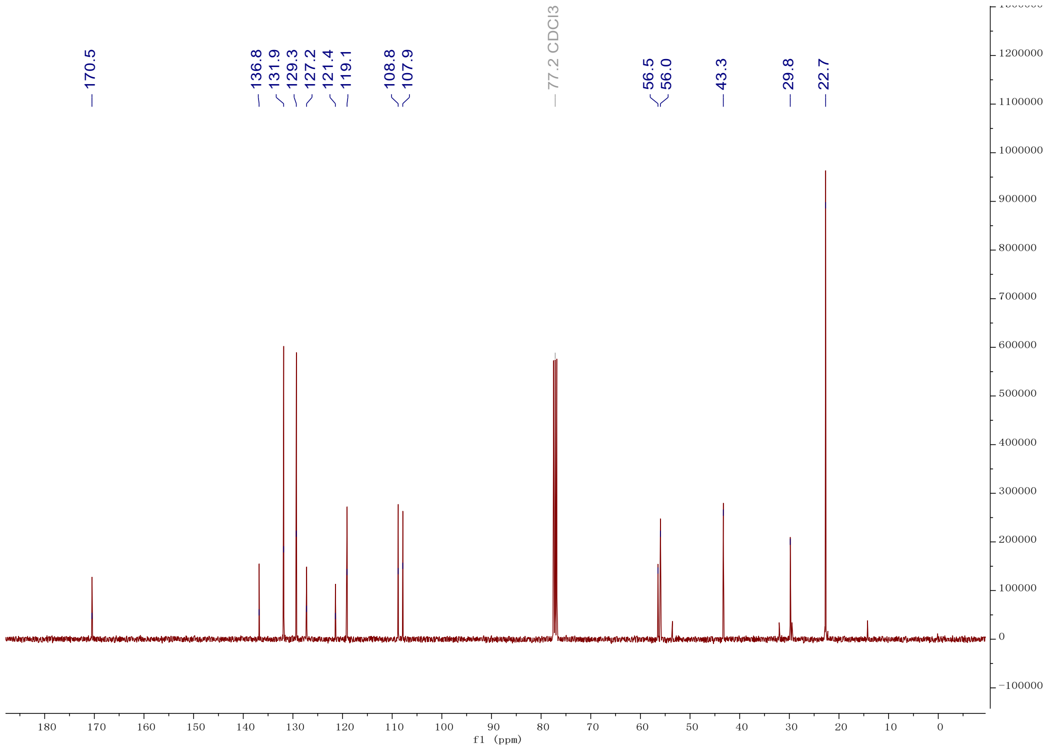

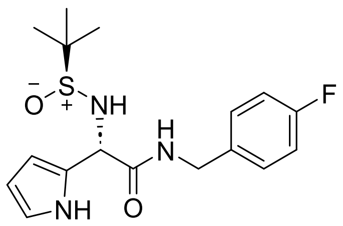
O
S
NH
H
N
F
NH
O

compound **32**

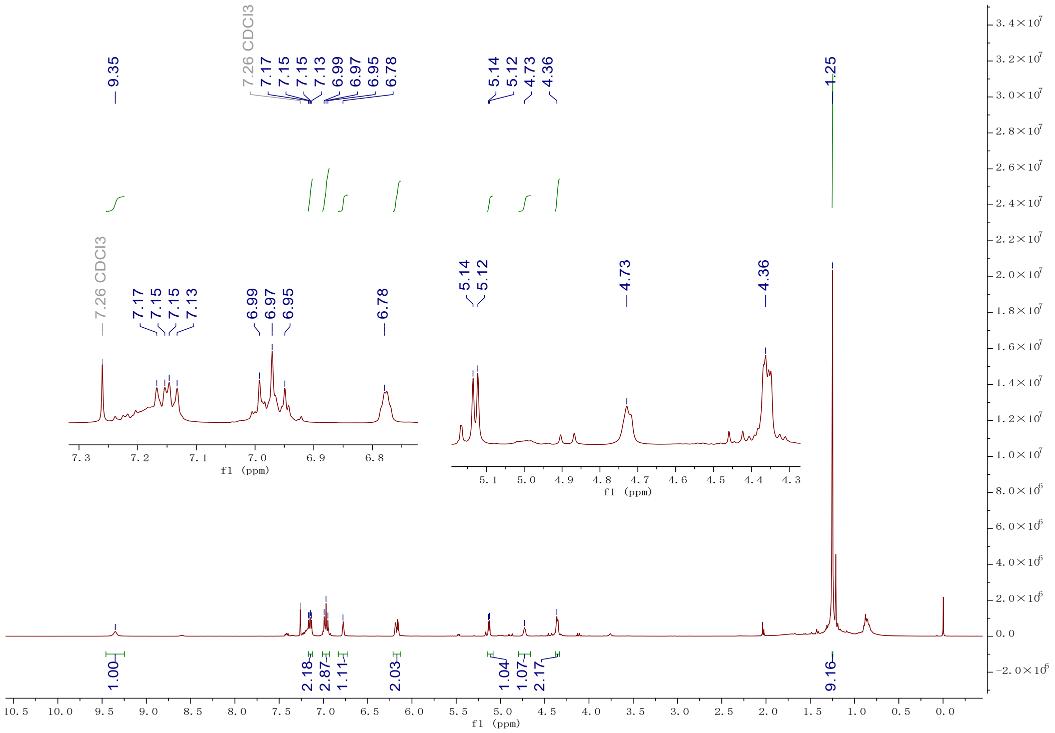
9.35
7.26 CDCl3
7.17
7.15
7.15
7.13
6.99
6.97
6.95
6.78
5.14
5.12
4.73
4.36
1.25
1.00
2.18
2.87
1.11
2.03
1.04
1.07
2.17
9.16
f1 (ppm)

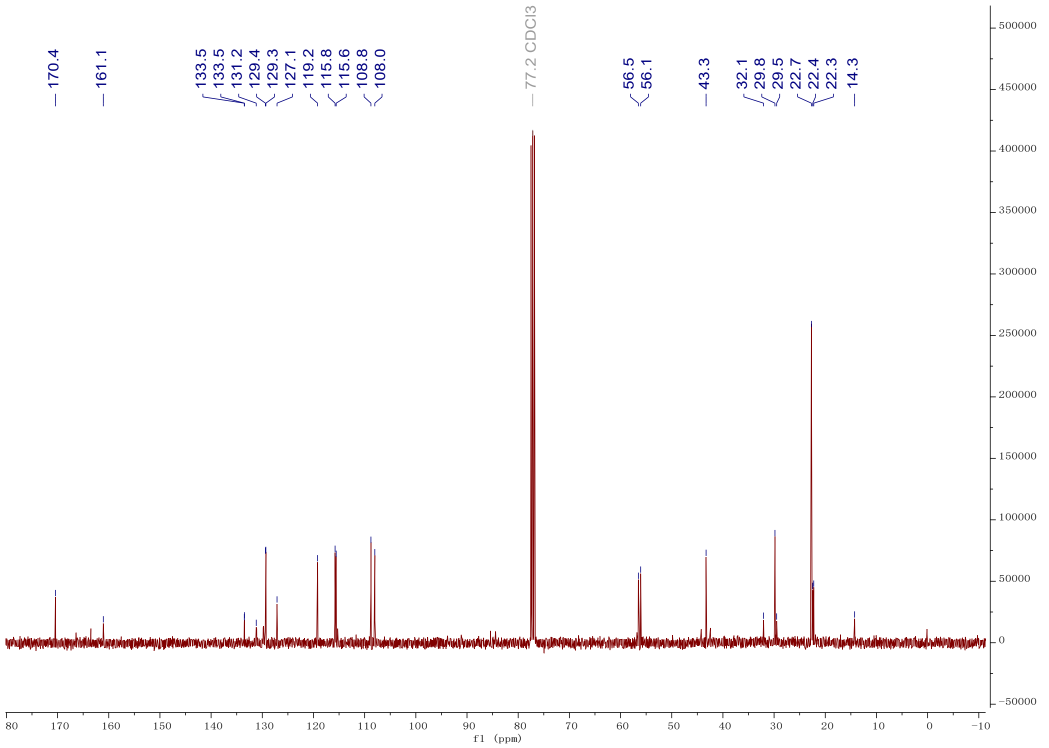
170.4
161.1
133.5
133.5
131.2
129.4
129.3
127.1
119.2
115.8
115.6
108.8
108.0
77.2 CDCl3
56.5
56.1
43.3
32.1
29.8
29.5
22.7
22.4
22.3
14.3
f1 (ppm)

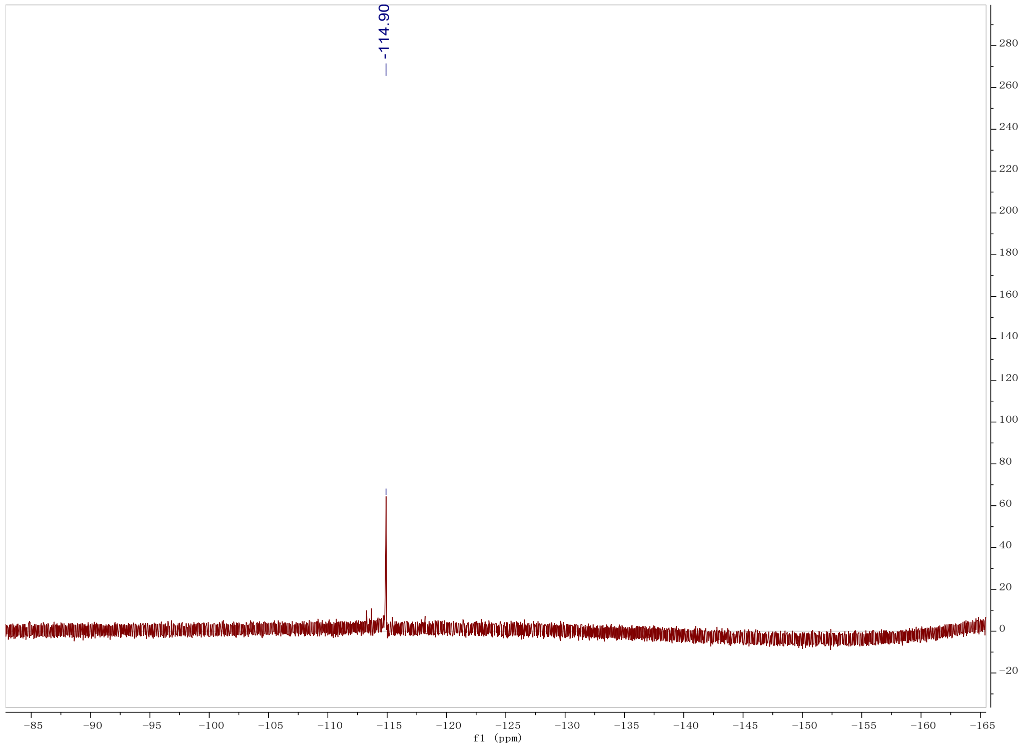
-114.90
f1 (ppm)

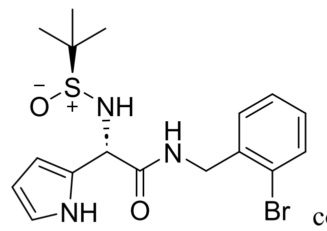
O
S
NH
H
N
NH
O
Br

compound **33**

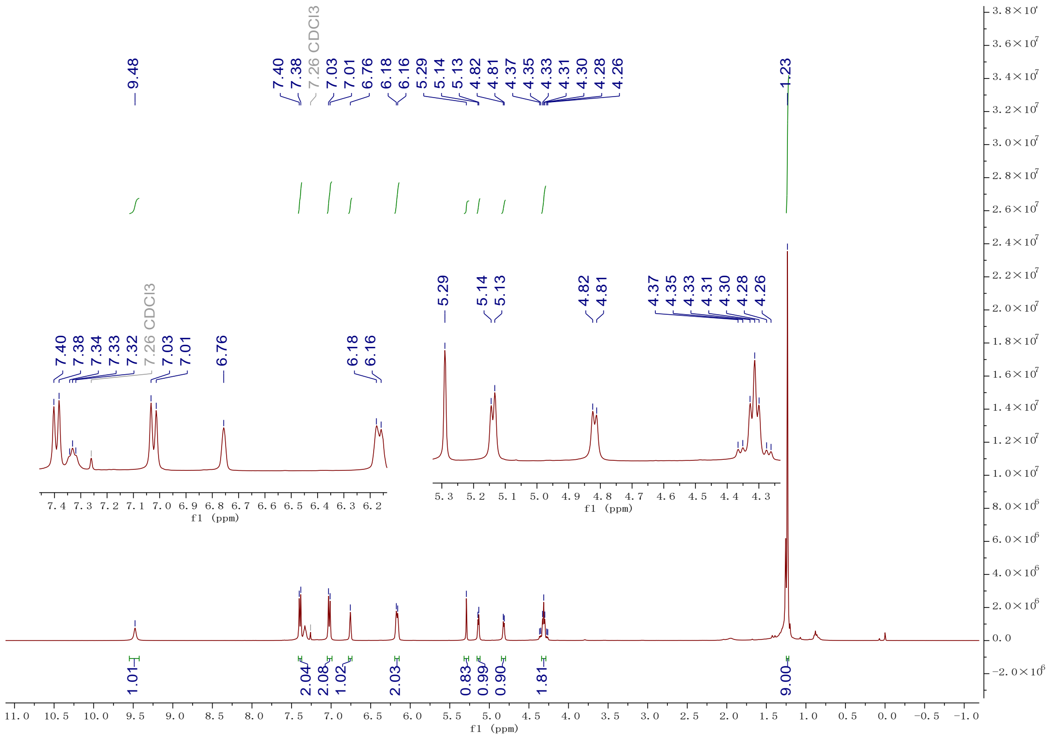
9.48
7.40
7.38
7.26 CDCl3
7.03
7.01
6.76
6.18
6.16
5.29
5.14
5.13
4.82
4.81
4.37
4.35
4.33
4.31
4.30
4.28
4.26
1.23
1.01
2.04
2.08
1.02
2.03
0.83
0.99
0.90
1.81
9.00
f1 (ppm)

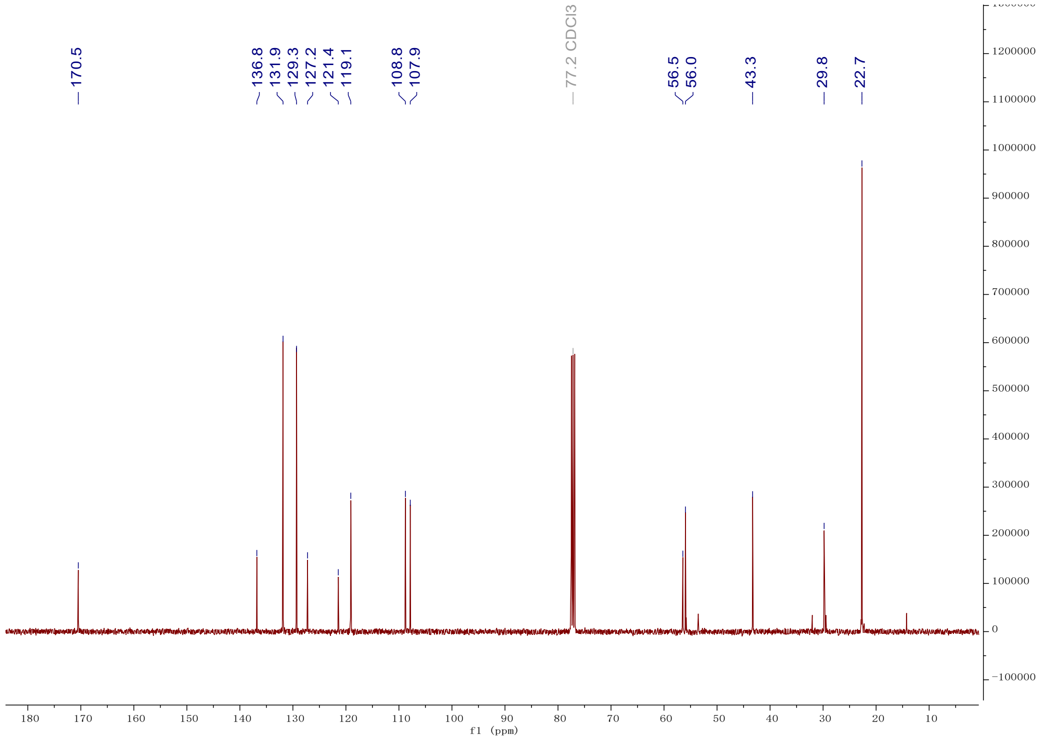
170.5
136.8
131.9
129.3
127.2
121.4
119.1
108.8
107.9
77.2 CDCl3
56.5
56.0
43.3
29.8
22.7
f1 (ppm)

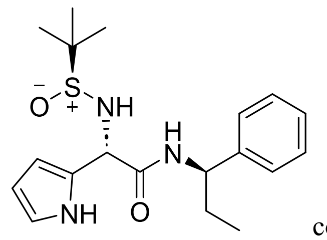

compound **34**

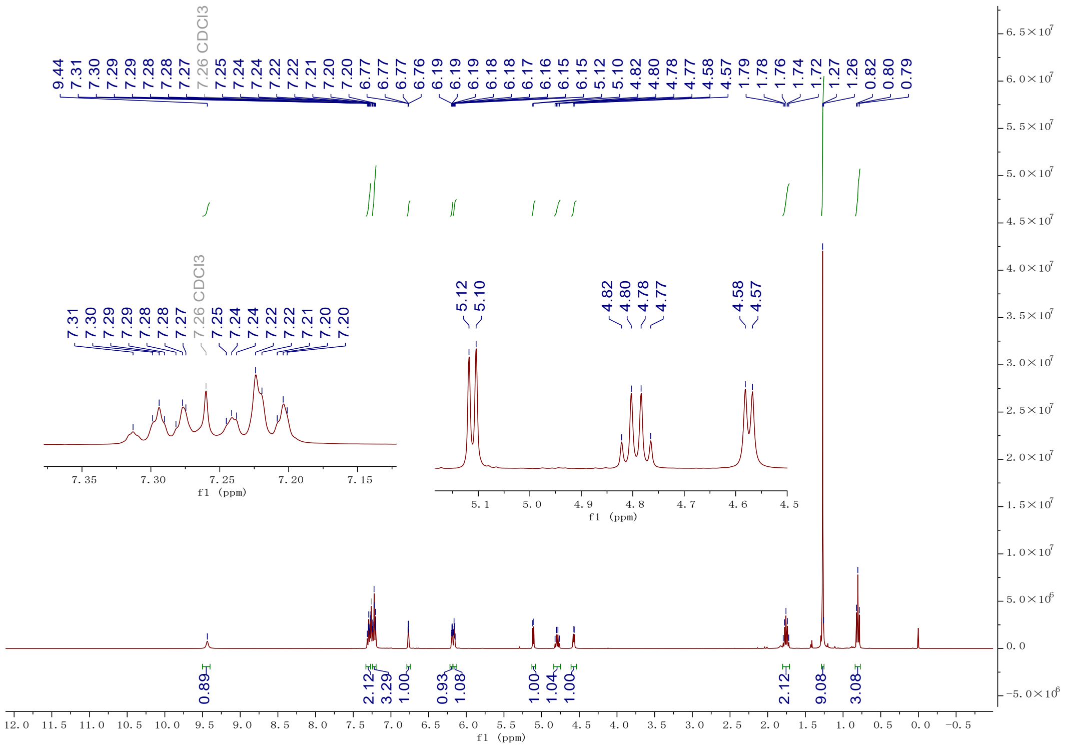

9.44 7.31 7.30 7.29 7.29 7.28 7.28 7.27 7.26 CDCl3 7.25 7.24 7.24 7.22 7.22 7.21 7.20 7.20 6.77 6.77 6.76 6.19 6.19 6.18 6.18 6.17 6.16 6.15 6.15 5.12 5.10 4.82 4.80 4.78 4.77 4.58 4.57 1.79 1.78 1.76 1.74 1.72 1.27 1.26 0.82 0.80 0.79
0.89 2.12 3.29 1.00 0.93 1.08 1.00 1.04 1.00 2.12 9.08 3.08
f1 (ppm)

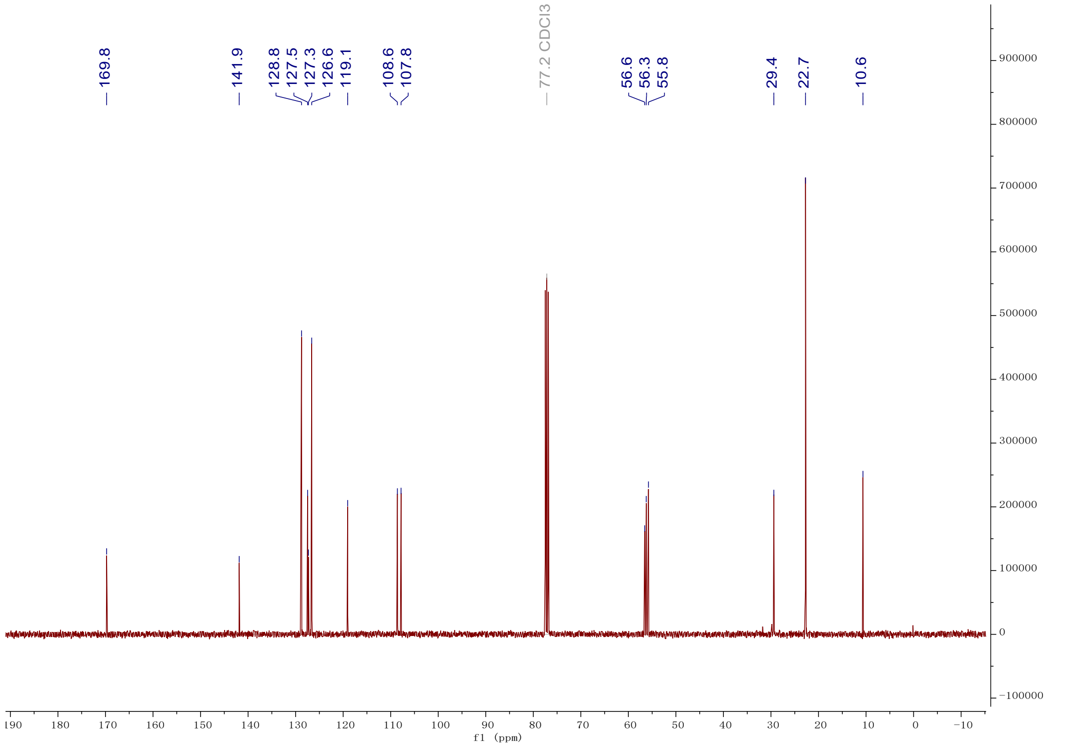

169.8 141.9 128.8 127.5 127.3 126.6 119.1 108.6 107.8 77.2 CDCl3 56.6 56.3 55.8 29.4 22.7 10.6
f1 (ppm)

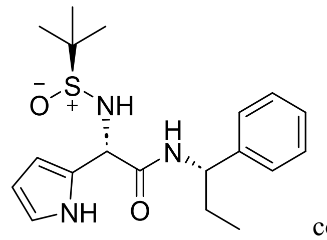
NH
H
N
NH
O
O
S

compound **35**

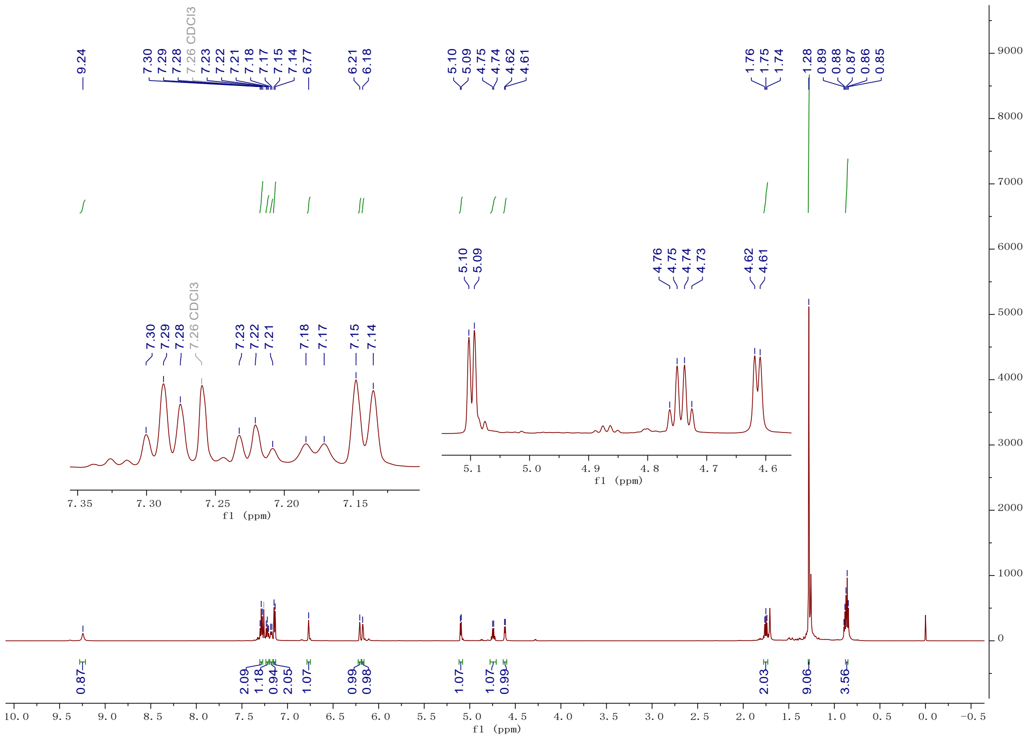
9.24
7.30 7.29 7.28 7.26 CDCl3 7.23 7.22 7.21 7.18 7.17 7.15 7.14
6.77
6.21 6.18
5.10 5.09 4.75 4.74 4.62 4.61
1.76 1.75 1.74
1.28 0.89 0.88 0.87 0.86 0.85
0.87 2.09 1.18 0.94 2.05 1.07 0.99 0.98 1.07 1.07 0.99 2.03 9.06 3.56
f1 (ppm)

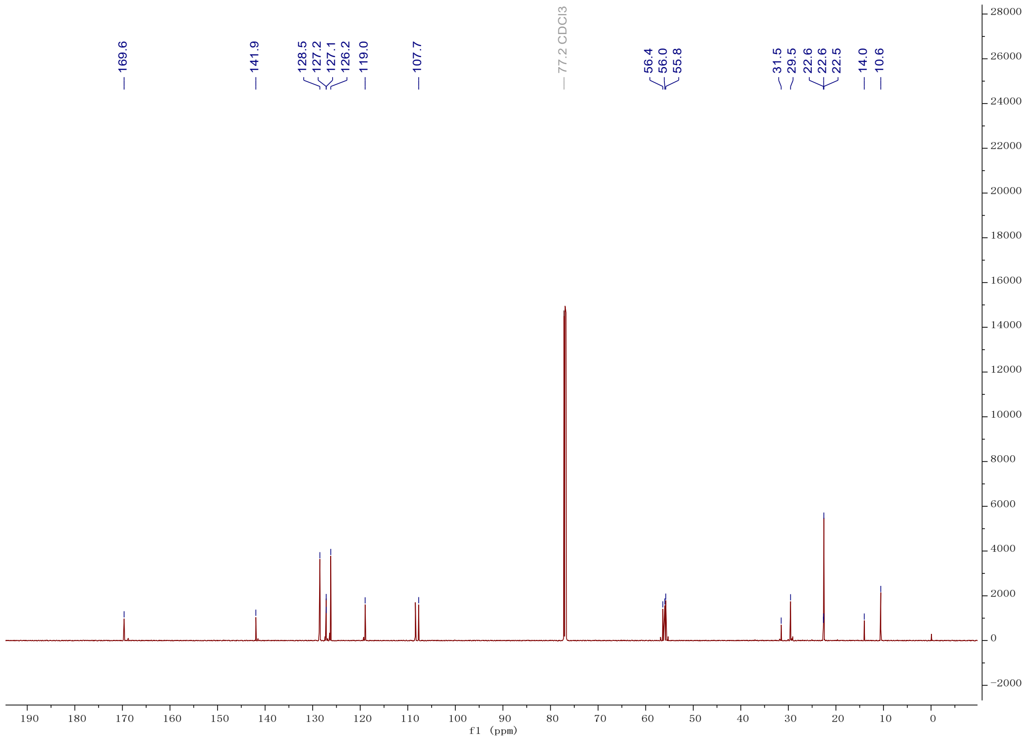
169.6
141.9
128.5 127.2 127.1 126.2 119.0
107.7
77.2 CDCl3
56.4 56.0 55.8
31.5 29.5 22.6 22.6 22.5 14.0 10.6
f1 (ppm)

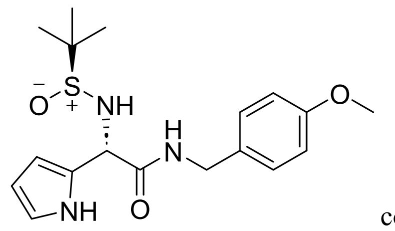
O
S
NH
H
N
O
NH
O

compound **36**

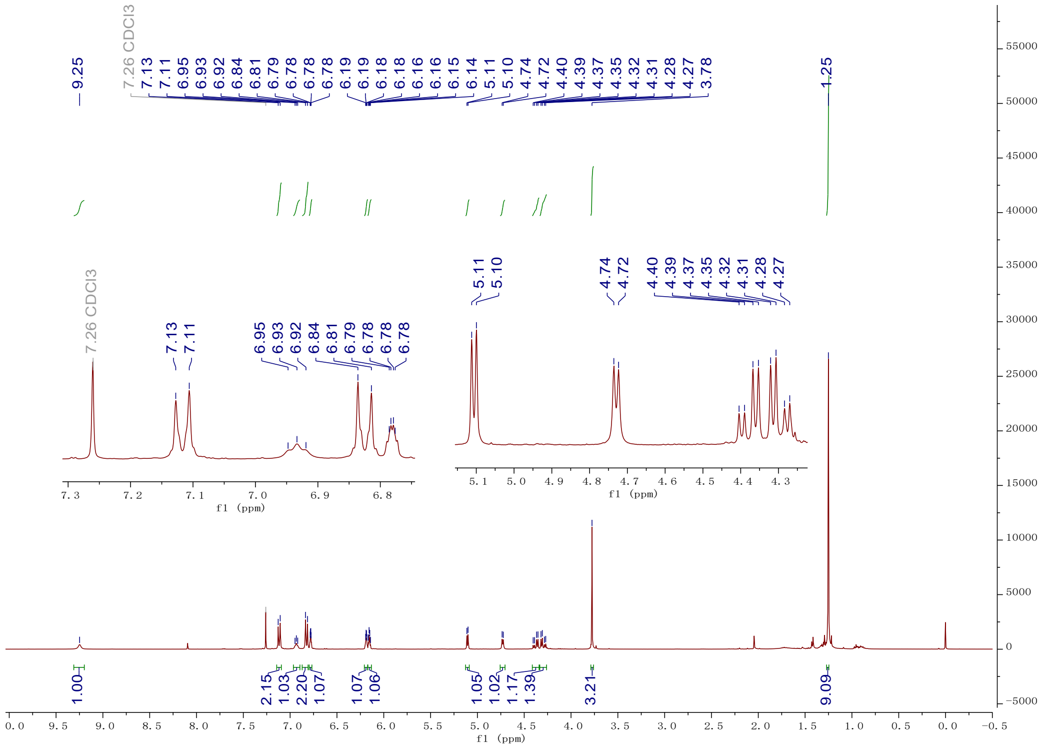
9.25
7.26 CDCl3
7.13
7.11
6.95
6.93
6.92
6.84
6.81
6.79
6.78
6.78
6.78
6.19
6.19
6.18
6.18
6.16
6.16
6.15
6.14
5.11
5.10
4.74
4.72
4.40
4.39
4.37
4.35
4.32
4.31
4.28
4.27
3.78
1.25
1.00
2.15
1.03
2.20
1.07
1.07
1.06
1.05
1.02
1.17
1.39
3.21
9.09
f1 (ppm)

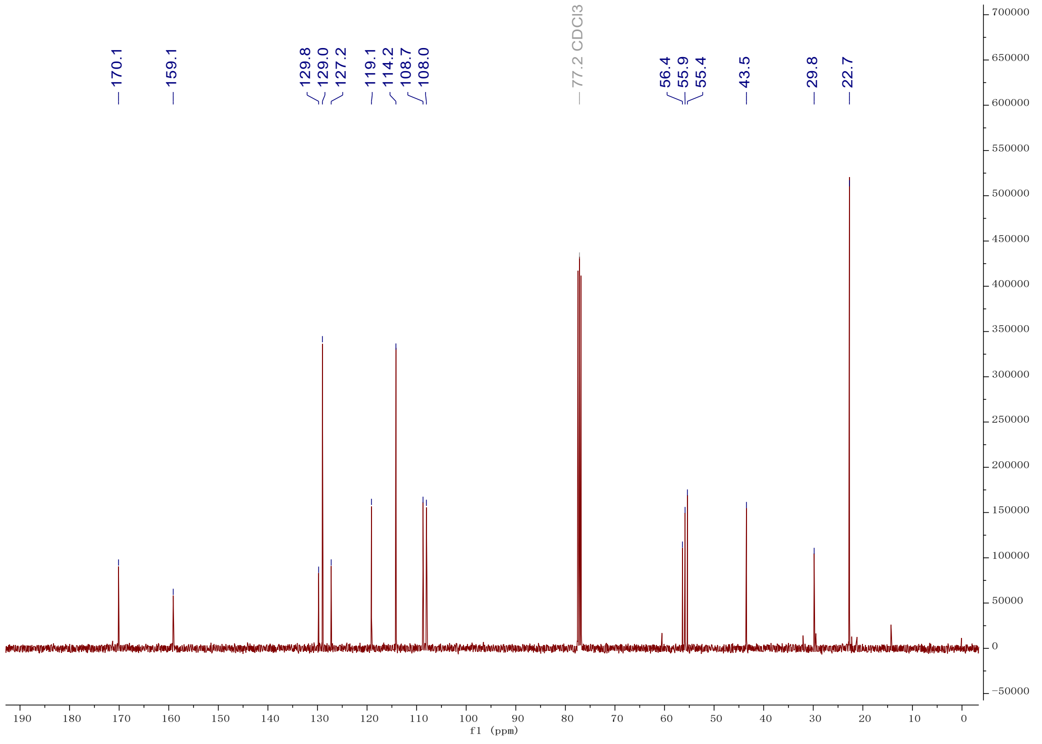
170.1
159.1
129.8
129.0
127.2
119.1
114.2
108.7
108.0
77.2 CDCl3
56.4
55.9
55.4
43.5
29.8
22.7
f1 (ppm)

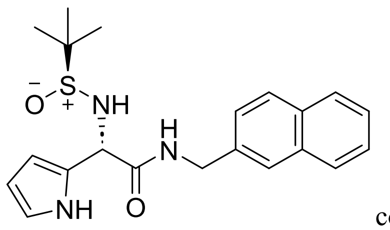
O
S
NH
H
N
NH
O

compound **37**

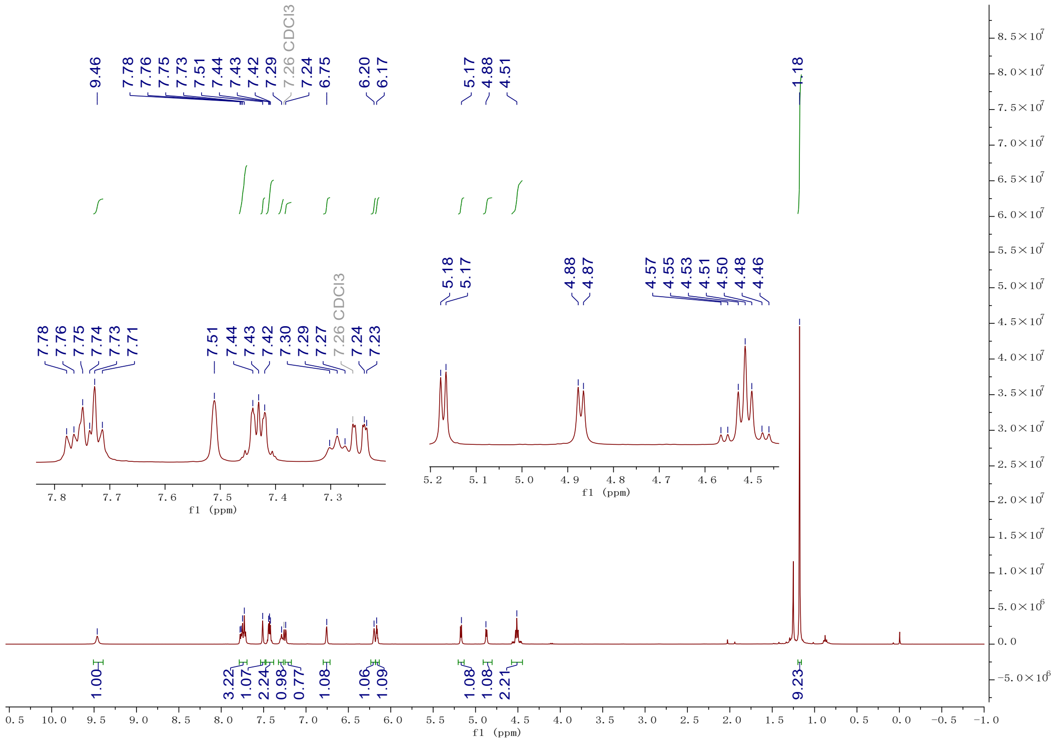
9.46
7.78
7.76
7.75
7.73
7.51
7.44
7.43
7.42
7.29
7.26 CDCl3
7.24
6.75
6.20
6.17
5.17
4.88
4.51
1.18
1.00
3.22
1.07
2.24
0.98
0.77
1.08
1.06
1.09
1.08
1.08
2.21
9.23
f1 (ppm)

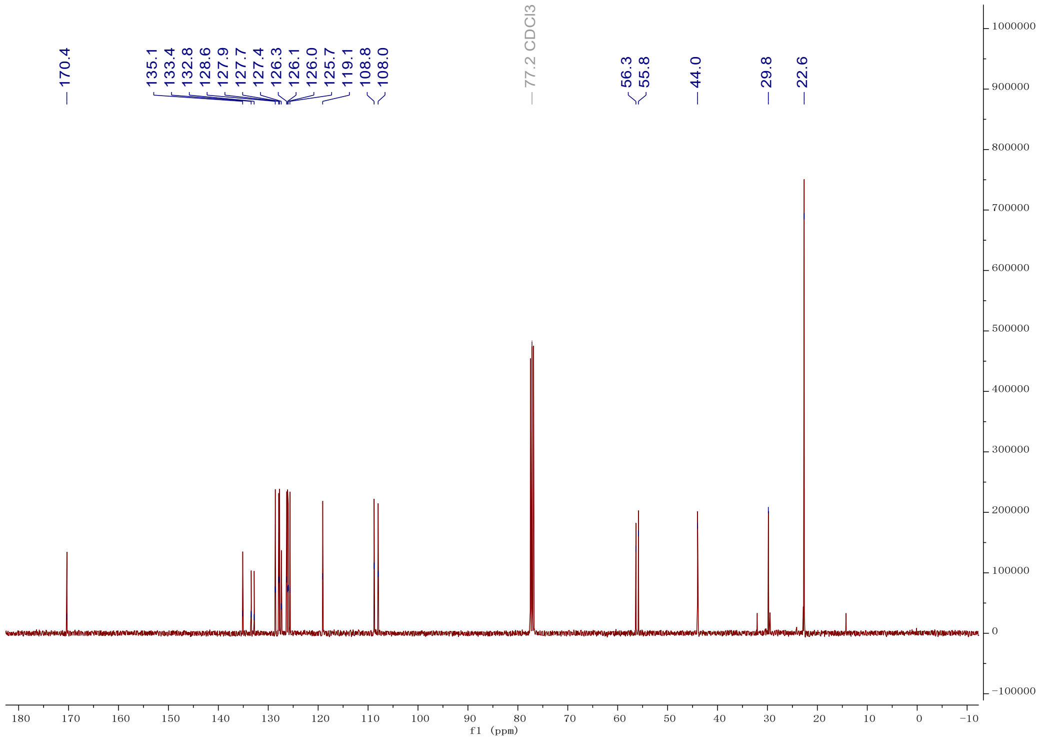
170.4
135.1
133.4
132.8
128.6
127.9
127.7
127.4
126.3
126.1
126.0
125.7
119.1
108.8
108.0
77.2 CDCl3
56.3
55.8
44.0
29.8
22.6
f1 (ppm)

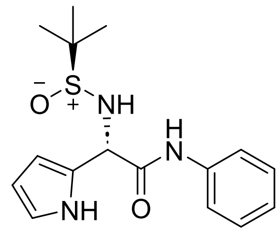

compound **38**

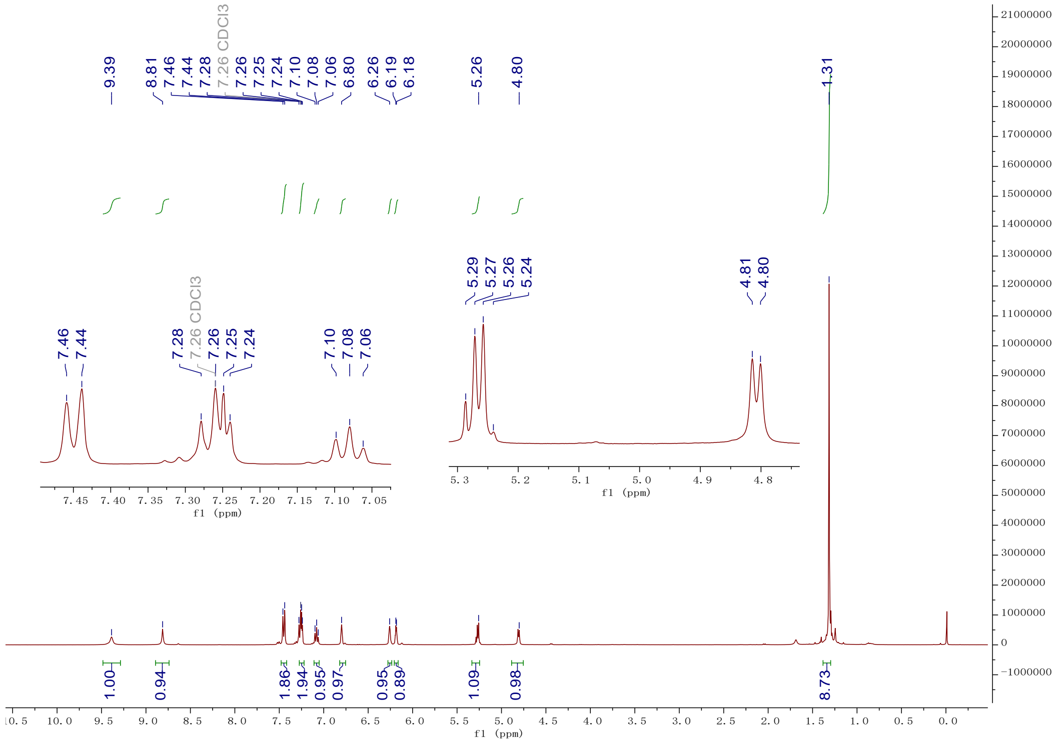

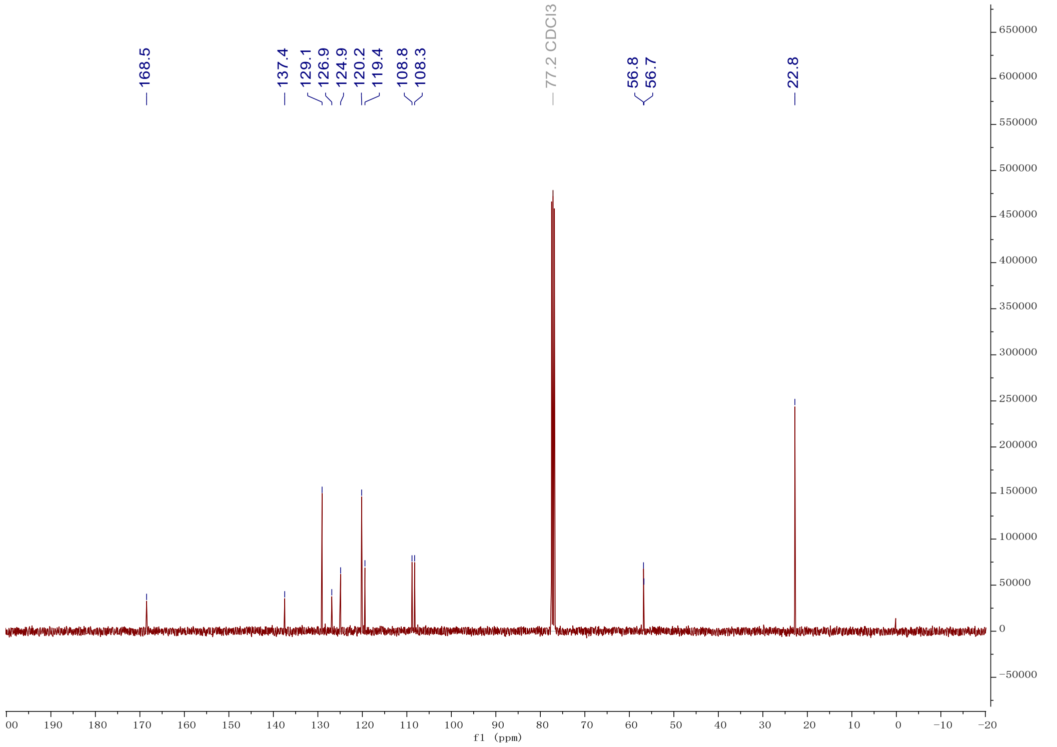

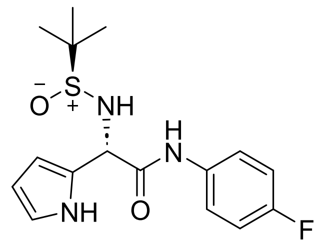
S
NH
H
N
NH
O
O
F

compound **39**

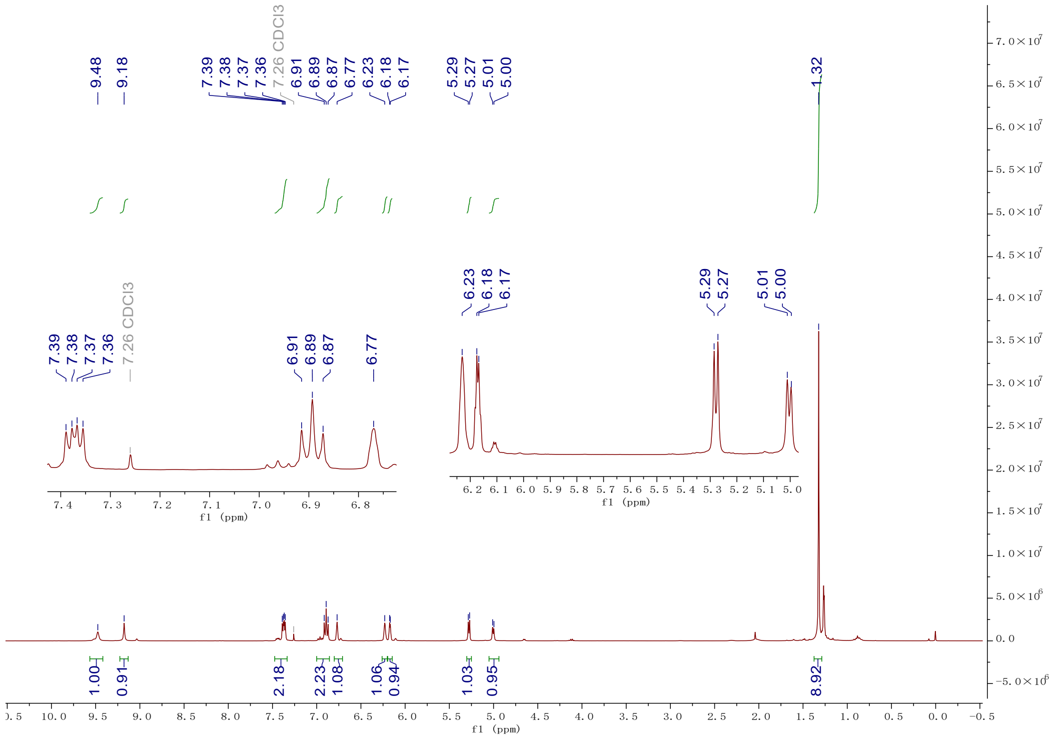
9.48
9.18
7.39
7.38
7.37
7.36
7.26 CDCl3
6.91
6.89
6.87
6.77
6.23
6.18
6.17
5.29
5.27
5.01
5.00
1.32
1.00
0.91
2.18
2.23
1.08
1.06
0.94
1.03
0.95
8.92
f1 (ppm)

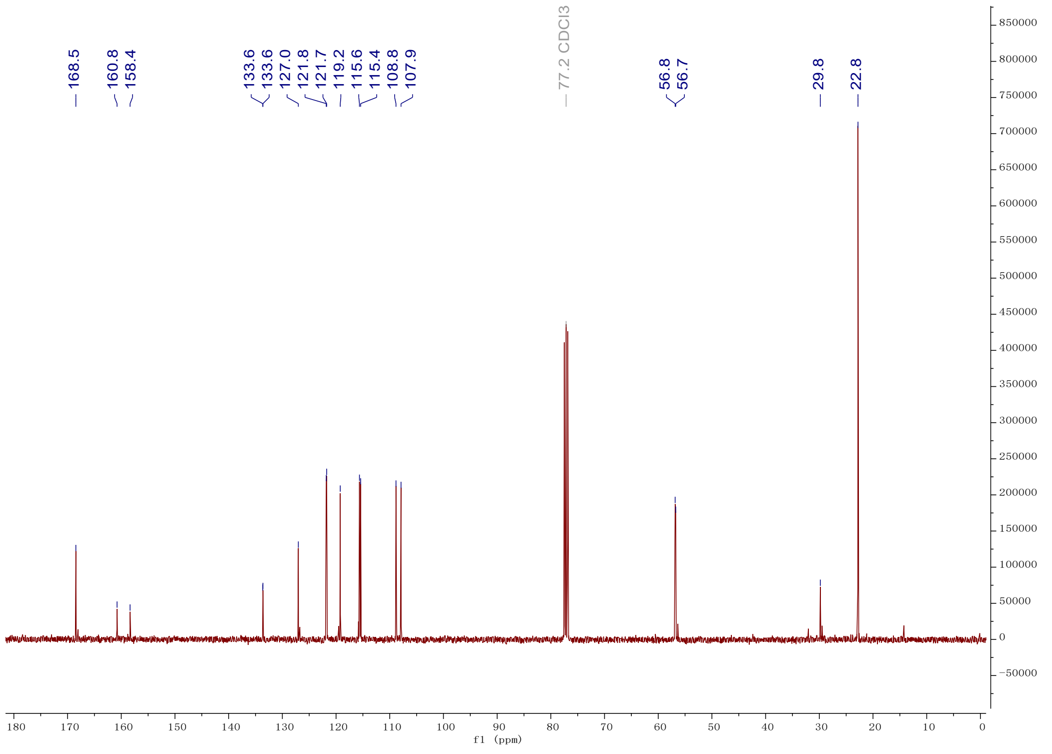
168.5
160.8
158.4
133.6
133.6
127.0
121.8
121.7
119.2
115.6
115.4
108.8
107.9
77.2 CDCl3
56.8
56.7
29.8
22.8
f1 (ppm)

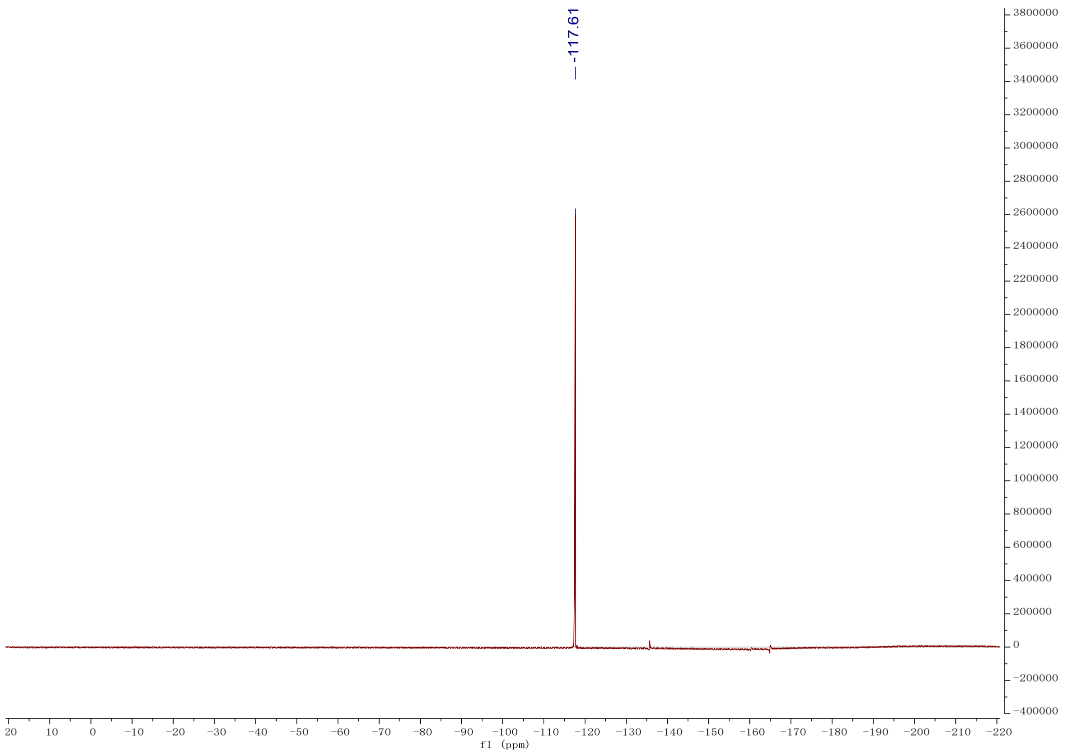
-117.61
f1 (ppm)

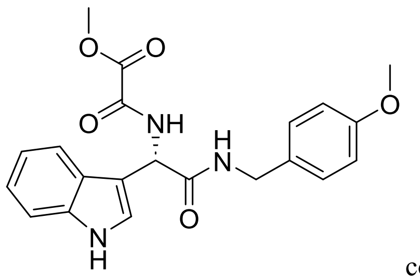

compound **40**

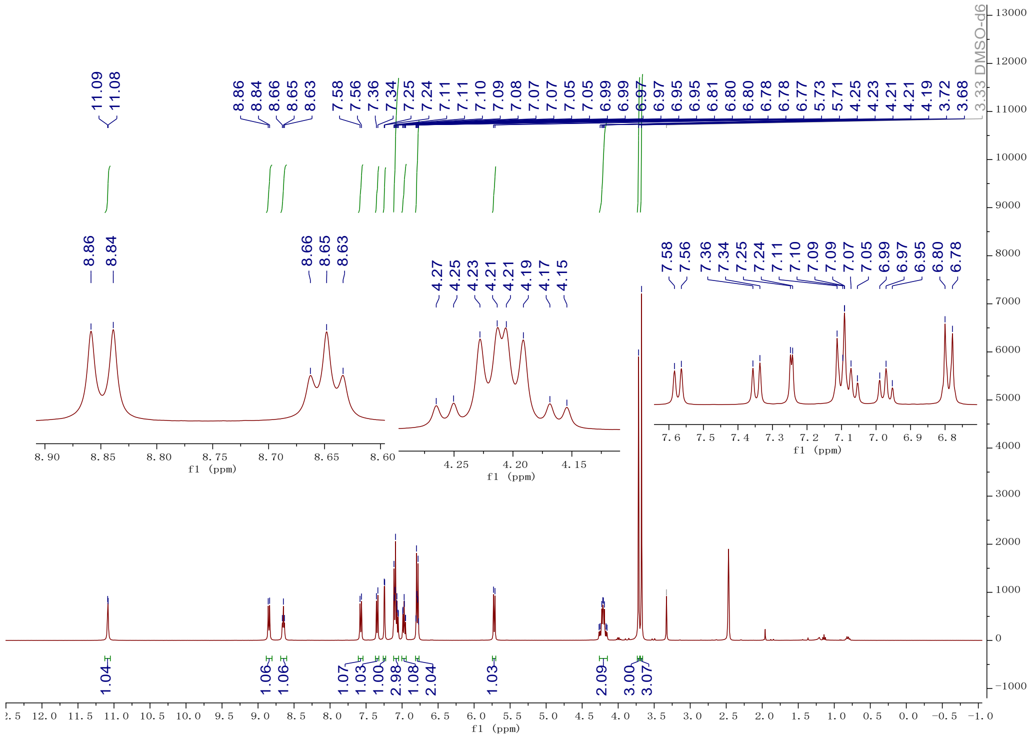

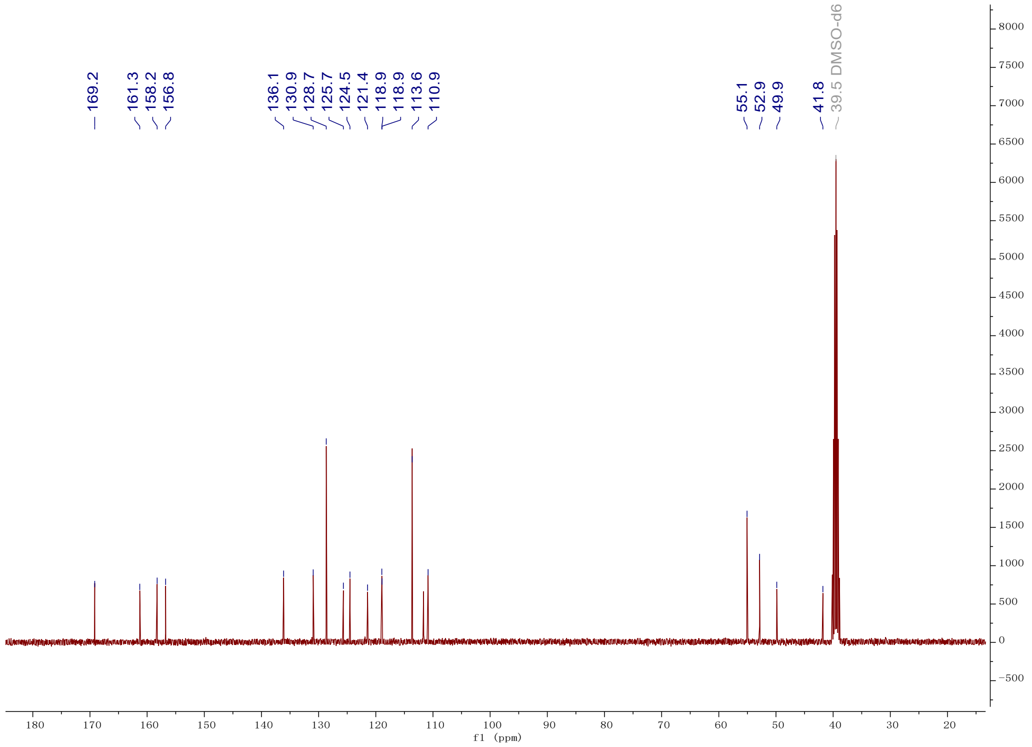

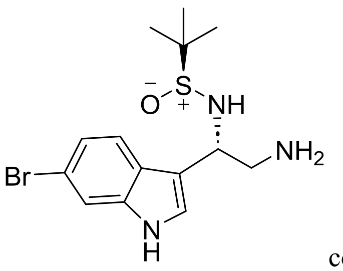

compound **41**

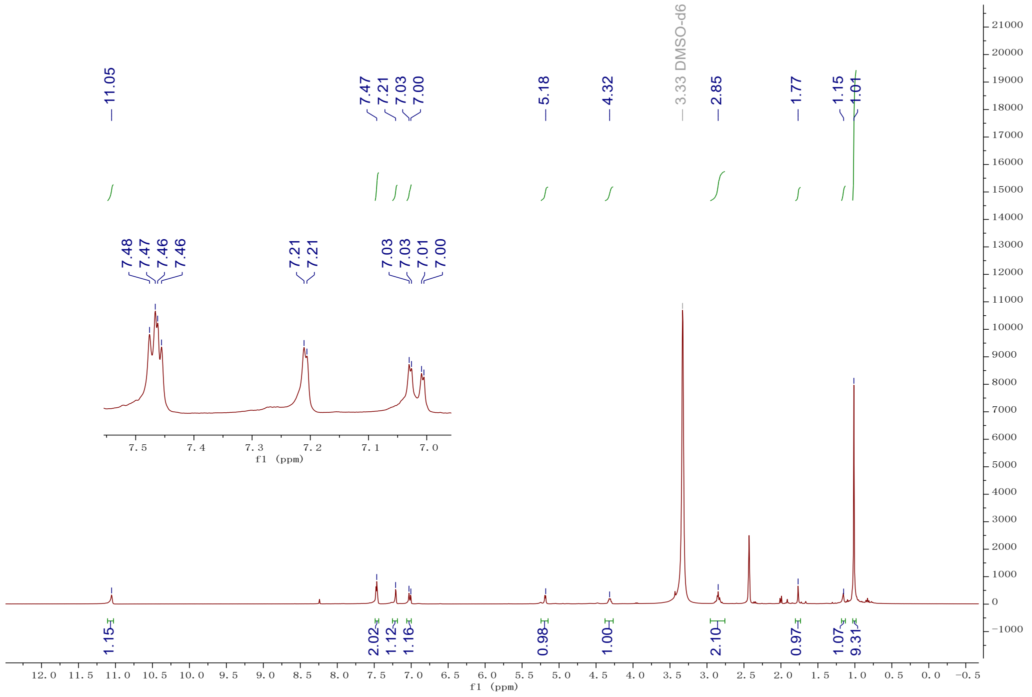

11.05
7.47
7.21
7.03
7.00
5.18
4.32
3.33 DMSO-d6
2.85
1.77
1.15
1.01
7.48
7.47
7.46
7.46
7.21
7.21
7.03
7.03
7.01
7.00
1.15
2.02
1.12
1.16
0.98
1.00
2.10
0.97
1.07
9.31
f1 (ppm)

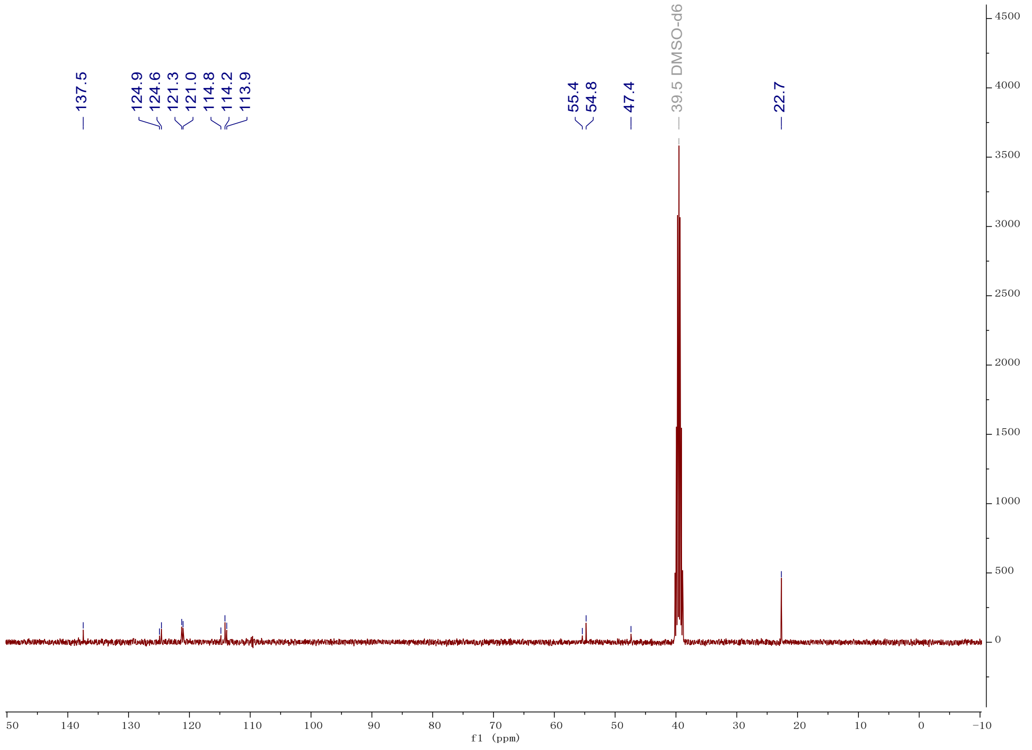

137.5
124.9
124.6
121.3
121.0
114.8
114.2
113.9
55.4
54.8
47.4
39.5 DMSO-d6
22.7
f1 (ppm)

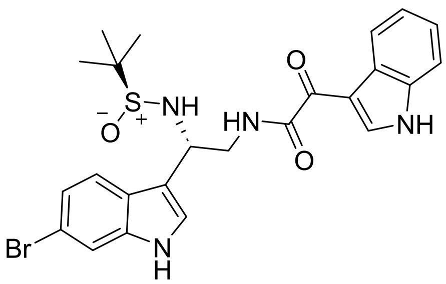

compound **42**

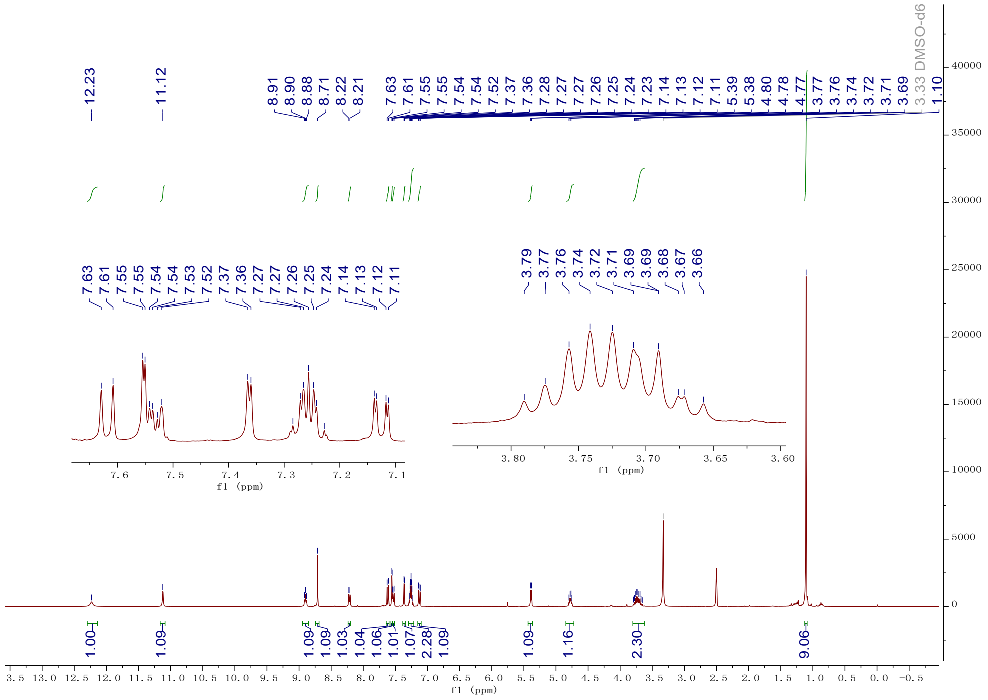

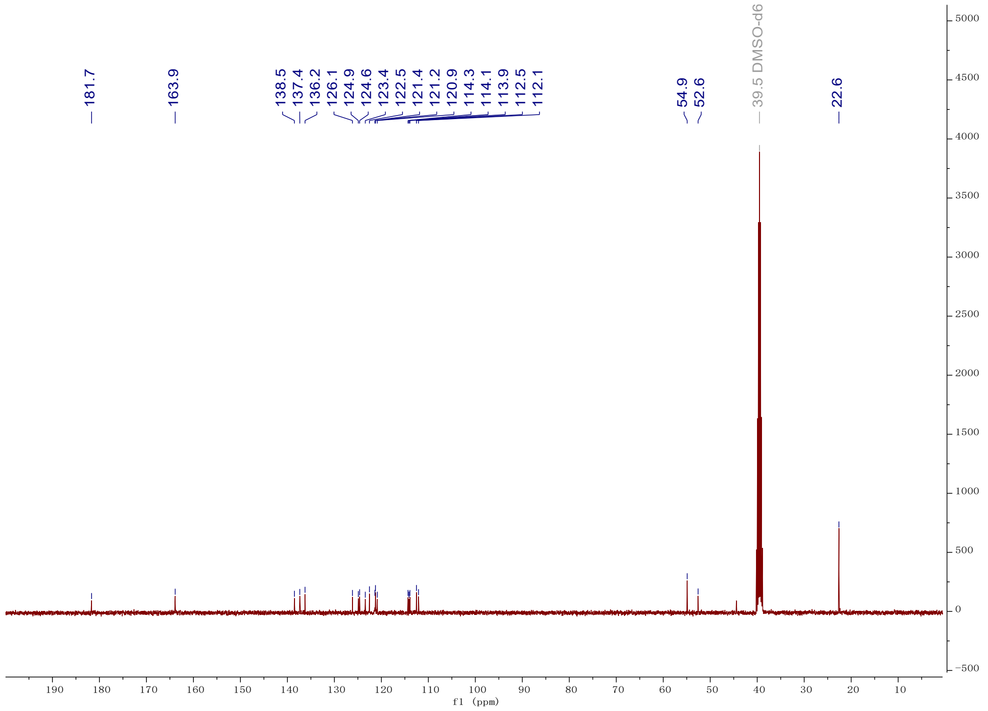

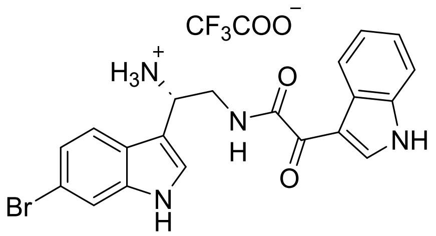

Calcicamide B

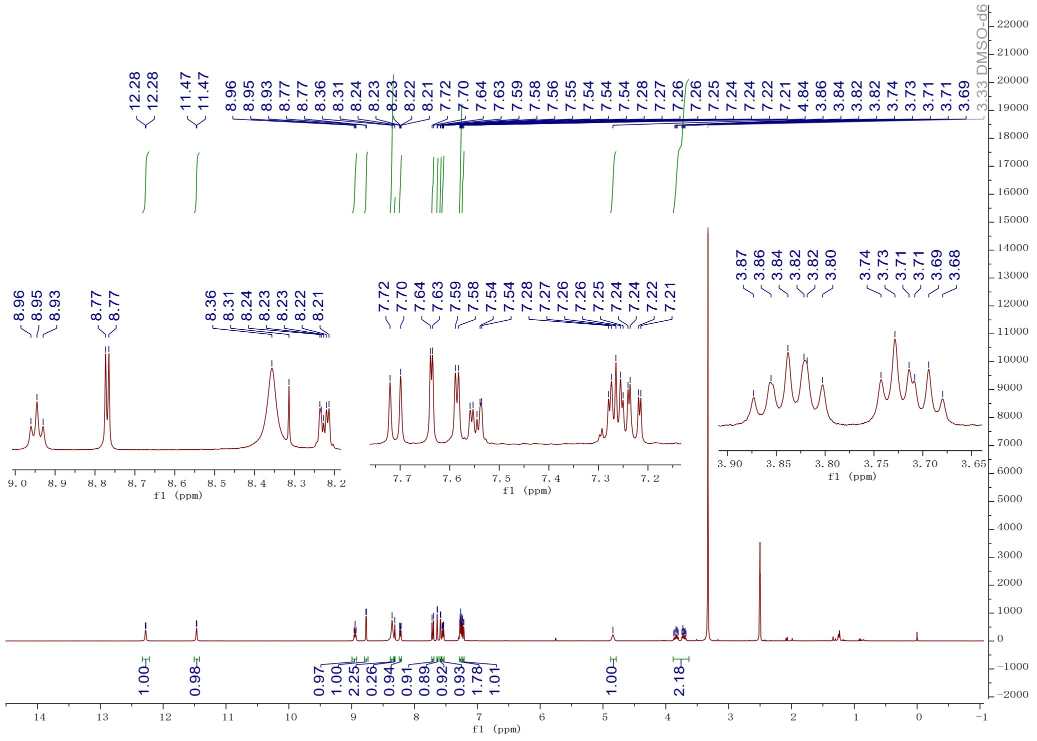

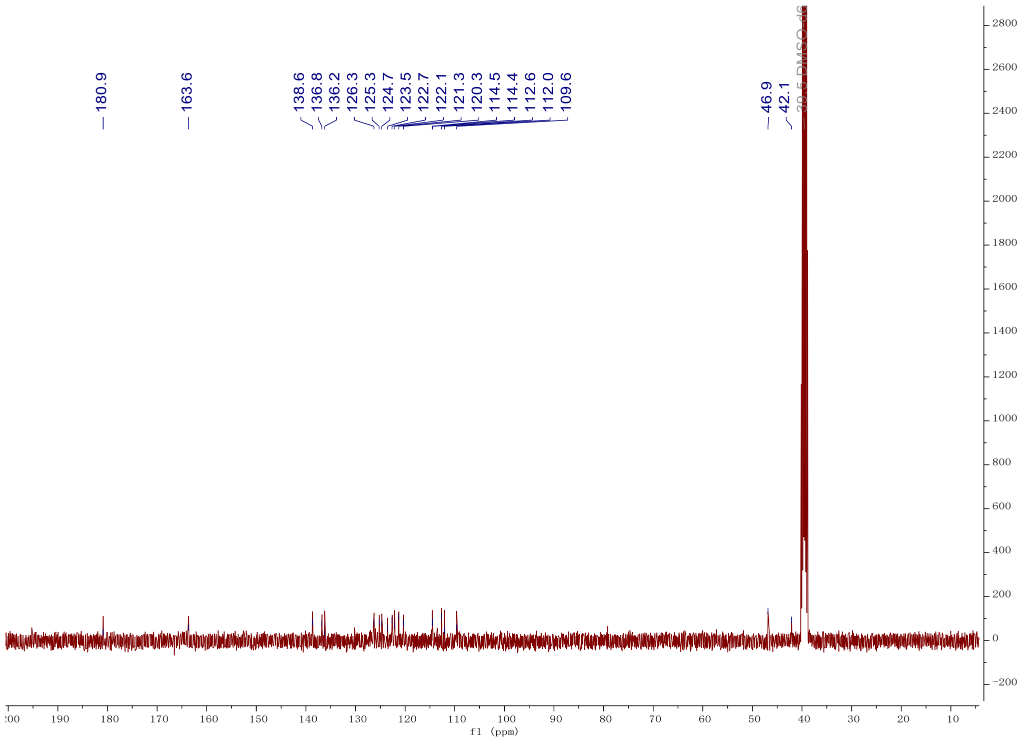

Supplement: RA-015-D5RA06138E-s001 [file RA-015-D5RA06138E-s001.pdf]
